# Quantitative magnetization transfer imaging in relapsing-remitting multiple sclerosis: a systematic review and meta-analysis

**DOI:** 10.1093/braincomms/fcac088

**Published:** 2022-04-04

**Authors:** Elizabeth N. York, Michael J. Thrippleton, Rozanna Meijboom, David P. J. Hunt, Adam D. Waldman

**Affiliations:** 1 Centre for Clinical Brain Sciences, University of Edinburgh, Edinburgh, UK; 2 UK Dementia Research Institute, University of Edinburgh, Edinburgh, UK; 3 Anne Rowling Regenerative Neurology Clinic, University of Edinburgh, Edinburgh, UK

**Keywords:** magnetization transfer, brain, multiple sclerosis, relapsing-remitting, systematic review

## Abstract

Myelin-sensitive MRI such as magnetization transfer imaging has been widely used in multiple sclerosis. The influence of methodology and differences in disease subtype on imaging findings is, however, not well established. Here, we systematically review magnetization transfer brain imaging findings in relapsing-remitting multiple sclerosis. We examine how methodological differences, disease effects and their interaction influence magnetization transfer imaging measures. Articles published before 06/01/2021 were retrieved from online databases (PubMed, EMBASE and Web of Science) with search terms including ‘magnetization transfer’ and ‘brain’ for systematic review, according to a pre-defined protocol. Only studies that used human *in vivo* quantitative magnetization transfer imaging in adults with relapsing-remitting multiple sclerosis (with or without healthy controls) were included. Additional data from relapsing-remitting multiple sclerosis subjects acquired in other studies comprising mixed disease subtypes were included in meta-analyses.

Data including sample size, MRI acquisition protocol parameters, treatments and clinical findings were extracted and qualitatively synthesized. Where possible, effect sizes were calculated for meta-analyses to determine magnetization transfer (i) differences between patients and healthy controls; (ii) longitudinal change and (iii) relationships with clinical disability in relapsing-remitting multiple sclerosis. Eighty-six studies met inclusion criteria. MRI acquisition parameters varied widely, and were also underreported. The majority of studies examined the magnetization transfer ratio in white matter, but magnetization transfer metrics, brain regions examined and results were heterogeneous. The analysis demonstrated a risk of bias due to selective reporting and small sample sizes. The pooled random-effects meta-analysis across all brain compartments revealed magnetization transfer ratio was 1.17 per cent units (95% CI −1.42 to −0.91) lower in relapsing-remitting multiple sclerosis than healthy controls (*z*-value: −8.99, *P* < 0.001, 46 studies). Linear mixed-model analysis did not show a significant longitudinal change in magnetization transfer ratio across all brain regions [*β* = 0.12 (−0.56 to 0.80), *t*-value = 0.35, *P* = 0.724, 14 studies] or normal-appearing white matter alone [*β* = 0.037 (−0.14 to 0.22), *t*-value = 0.41, *P* = 0.68, eight studies]. There was a significant negative association between the magnetization transfer ratio and clinical disability, as assessed by the Expanded Disability Status Scale [*r* = −0.32 (95% CI −0.46 to −0.17); *z*-value = −4.33, *P* < 0.001, 13 studies]. Evidence suggests that magnetization transfer imaging metrics are sensitive to pathological brain changes in relapsing-remitting multiple sclerosis, although effect sizes were small in comparison to inter-study variability. Recommendations include: better harmonized magnetization transfer acquisition protocols with detailed methodological reporting standards; larger, well-phenotyped cohorts, including healthy controls; and, further exploration of techniques such as magnetization transfer saturation or inhomogeneous magnetization transfer ratio.

## Teaser

York *et al*. systematically reviewed 86 studies of magnetization transfer brain imaging in relapsing-remitting multiple sclerosis. Meta-analyses showed that magnetization transfer was reduced in patients compared with controls, but results were heterogeneous, longitudinal change subtle and associations with clinical disability weak. Better harmonized study acquisition protocols in larger, well-phenotyped cohorts are warranted.

## Graphical Abstract

**Graphical Abstract:**
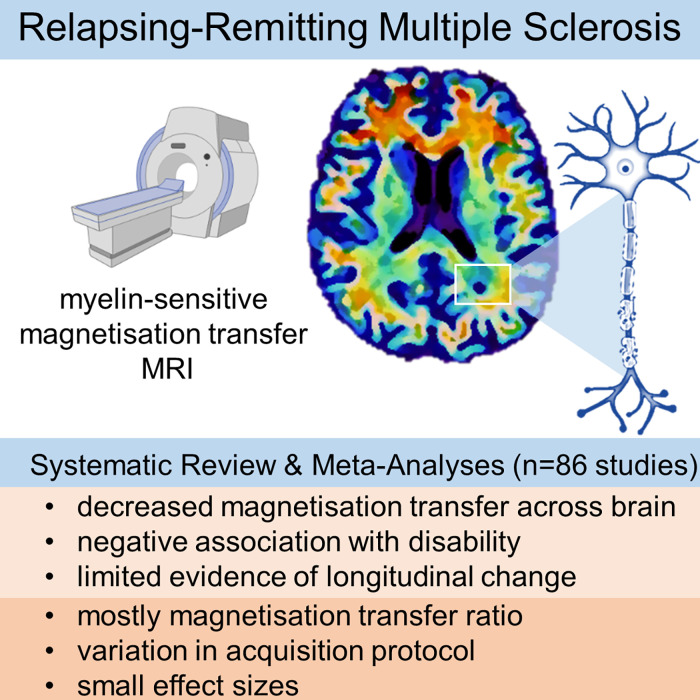

## Introduction

### Multiple sclerosis: a heterogeneous disease

Multiple sclerosis (MS) is an immune-mediated disease involving widespread focal injury (lesions) to myelin—the fatty sheath which insulates neuronal axons—and nerve fibres within the CNS, accompanied by neuroinflammation.^1^ This results in irreversible neurodegeneration.

Demyelination and neuronal damage manifest as heterogeneous clinical disability such as weakness, visual disturbances and cognitive impairment. Acute clinical episodes, or relapses, define the relapsing-remitting MS (RRMS) subtype and are often accompanied by new lesions on MRI. Although diverse in pathological appearance, lesions are indicative of inflammation and demyelination. In RRMS, relapses are interspersed with periods of stability or remission, although the clinical course varies and the choice of effective disease-modifying therapies (DMTs) is currently limited.

Reliable, non-invasive *in vivo* biomarkers are necessary to predict and track disease progression in individuals, and objectively assess the effectiveness of both current and emerging treatments.^2^ The relationship between clinical disability and conventional MRI measures of disease burden such as lesion load visible on T_2_-weighted (T2-w) imaging^3^ and atrophy^4^ is, however, weak. This reflects a need for validated quantitative MRI metrics which are more sensitive and specific to disease-related pathological microstructural change in RRMS.

### Magnetization transfer imaging

Magnetization transfer imaging (MTI) is sensitive to subtle pathological changes in tissue microstructure which cannot typically be quantified with conventional MRI.^5,6^ MT signal is indirectly derived from protons ‘bound’ to macromolecules.^7^

Considering a simple two-pool model for hydrogen nuclei in the brain,^8^ the so-called ‘free’ pool of water protons shows relatively unrestricted diffusion and contributes to the bulk source of conventional MRI signal. Hydrogen nuclei in the ‘bound’ pool, however, are closely coupled to macromolecules (including lipids such as myelin) and have hindered rotational and translational motion, resulting in T2 decays too rapid (∼10 µs) for the signal to be detectable at typical echo times (TEs).

MTI exploits the continuous exchange of magnetization between pools to obtain signal indirectly from this ‘bound’ pool. Since the frequency spectrum of the ‘bound’ pool is much broader than the ‘free’ water peak, an applied off-resonance radiofrequency pulse may selectively saturate ‘bound’ protons. Magnetization exchange between the two pools reduces longitudinal magnetization of the ‘free’ pool and hence it’s signal intensity. Among other factors, the magnitude of this effect depends on the size of the ‘bound’ pool, which hence provides a surrogate marker of myelin integrity. MTI has therefore been used to study white matter (WM) diseases, including MS.^6,9^

### Quantifying magnetization transfer

Magnetization transfer ratio (MTR), calculated as the percentage change in signal with and without a saturation pulse (Video 1), has been widely applied in clinical studies due to relatively brief acquisition and ease of calculation. MTR is, however, susceptible to field inhomogeneities and T1 relaxation effects, and varies widely depending upon specific acquisition parameters [e.g. repetition time (TR), excitation flip angle, sequence type, saturation pulse offset, power, shape and duration].^10^ Biological interpretation of MTR, as well as inter-site and inter-study comparisons, are therefore challenging, and present a barrier to clinical translation.

Magnetization transfer saturation (MTsat) inherently corrects for B1 inhomogeneities and T1 relaxation,^11^ by approximating the signal amplitude and T1 relaxation at low flip angles with an additional T_1_-weighted (T1-w) image.^11,12^ MTsat hence addresses some limitations of MTR, within clinically feasible acquisition times and specific absorption rate limits, and the resulting parametric maps have visibly better tissue contrast than MTR (Video 1).^11^

Inhomogeneous MTR (ihMTR) exploits observed asymmetry of the broadened spectral line of the bound pool, thought to be driven by dipolar coupling effects,^13^ and compares single frequency saturation at positive and negative frequency offsets with simultaneous saturation at two frequencies (±).^14,15^ While not yet fully understood, ihMTR^15^ is thought to be particularly sensitive to highly restricted protons in lipid chains and therefore more specific to the phospholipid bilayer of myelin than other MTI methods.

Fully quantitative MTI [quantitative magnetization transfer (qMT)] approaches using multi-compartmental models describe MT effects most rigorously by systematically varying the saturation offset and power. Important derived parameters include the fractional pool size ratio (*F* or PSR), the relative macromolecular content (MMC) and the macromolecular proton fraction (*f*) which provide indicators of myelin content. Calculation of either *F* or *f* requires estimation of the longitudinal relaxation rate, *R*_1_, for each pool.^16^ The MT exchange rate from the bound to the free pool (*k_f_*) may also help to gauge myelin status. qMT is time-consuming to acquire, requires complex analysis and tends not to provide whole-brain coverage. qMT application has therefore mostly been limited to small-scale methodological studies.

### Rationale

Previous reviews provide an overview of qMT, MTI^17^ and its specific application in MS.^9,18,19^ More recently, Weiskopf *et al*.^20^ have provided a technical review of the concepts, validation and modelling of quantitative MRI, including qMT. The biophysical models used to describe MT effects in tissue, experimental evidence in brain development, ageing and pathology have also been reviewed.^6^ Lazari and Lipp^21^ and van der Weijden *et al*.^22^ systematically reviewed myelin-sensitive MRI validation, reproducibility and correlation with histology in humans and animal populations. Campbell *et al*.^23^ and Mohammadi and Callaghan^24^ have addressed incorporation of MTI-derived *g*-ratio measures to determine relative myelin-to-axon thickness.

The emergence of methods such as MTsat and ihMTR, which provide more specific measures of tissue microstructure than MTR but can be acquired relatively rapidly across the whole brain, present an opportunity to reassess the use of clinical MTI.^11,15,25^ An evaluation of the body of evidence for MTI as a marker of disease from diverse studies would allow a better understanding of the effects of technique and other sources of bias across apparently contradictory results in the literature. Moreover, differences in clinical course,^26^ current therapeutic approaches^27–29^ and CSF biomarker profiles reflecting dominant pathophysiology^30^ justify specific examination of the different MS subtypes. We believe therefore that a systematic review of myelin-sensitive MTI in RRMS with meta-analyses is warranted.

### Purpose

The aim of the present study is thus to systematically review (i) MTI techniques used to assess pathological change in RRMS and (ii) sources of inter-study variability and bias. We then aim to apply meta-analyses to provide consensus on (iii) key cross-sectional and longitudinal pathological findings and (iv) the relationship between MTI and clinical disability in RRMS.

## Materials and methods

Approval from an ethics committee was not required for the present review.

### Registration and protocol

This review was not registered. The protocol was set *a priori* as described but not registered externally.

### Search strategy and eligibility criteria

This review adhered to PRISMA guidelines.^31,32^ The search terms were ‘magnetisation transfer’ or ‘magnetization transfer’ and ‘brain’ (with MeSH terms). The online databases searched were PubMed, Embase and Web of Science.

Search and eligibility criteria were in accordance with a protocol that had been defined *a priori*. For inclusion, studies had to be primary human research and had to include people with RRMS. Because the focus of the review was on MTI findings and their correlates in RRMS, studies that included people with other MS subtypes (e.g. primary progressive) or post-mortem imaging data, were excluded from the main analysis. Articles in any language were accepted, with a publishing cut-off date of 06/01/2021.

Exclusion criteria were: inclusion of subjects with non-MS pathology (e.g. brain tumours, traumatic brain injury) where RRMS was not the main focus; paediatric (i.e. <18 years of age) or paediatric-onset MS; solely inclusion of healthy participants (i.e. without MS patients); the full text was not retrievable; only phantom, *in vitro*, preclinical *in vivo* or *ex vivo* data; study published before 1980; an imaging technique other than MTI used; non-brain imaging only; non-quantitative methodology; theoretical or simulation-only papers; a clinical trial protocol, Phase I or Phase II clinical trial; conference proceedings; a review or opinion article; and, any study clearly irrelevant to the current review. Duplicated datasets were not excluded, as these could not be identified reliably from the study publications.

### Search procedure

Search results were imported into EndNote. Duplicate publications were automatically removed using the in-built de-duplicator tool, and the remaining duplicates were removed manually. Abstracts were checked by the author (E.N.Y.) and removed when exclusion criteria were met. Full texts were manually retrieved by the author (E.N.Y.) with online searches for article DOIs, PMID or title. If this failed, the abstract was excluded. Full-text articles were screened manually by the author (E.N.Y.) for exclusion criteria and rejected where necessary. The remaining selection was categorized according to the MS subtype. Articles without RRMS cohorts or comprising mixed subtypes were excluded from the main review. MTI data for RRMS patients in excluded studies comprising mixed MS subtypes were, however, included in meta-analyses, where it was possible to identify and analyse these separately.

### Data extraction

Data were extracted in detail including demographics, acquisition parameters, MT measure and brain region, statistical methodology, summarized clinical findings and study limitations. Where possible, correlation coefficients, MT mean and standard deviation were extracted to calculate effect sizes for meta-analyses.

### Statistical analysis

Descriptive statistics were calculated for demographic data, DMTs and steroid usage, and clinical disability measures. Key study findings and limitations were collated according to the MT technique used and the brain region.

When data were available from a sufficient number of studies, random-effects meta-analyses, with brain region as a nested factor, were performed to determine:

1. differences in MT metrics between patients with RRMS and healthy controls (HCs) (significance level, *α* = 0.05, *metafor* package in RStudio v1.3.1093).
2. putative relationships between clinical disability and MT metrics, in studies with reported correlation coefficients.

Where the number of studies, *k*, was >2 for a given brain region, follow-up sub-analyses were carried out to determine regional effect sizes, corrected for multiple comparisons [*α* = 0.05/(1 + *n* of sub-analyses)]. The Sidik–Jonkman method was used to assess between-study heterogeneity. Means were standardized (Hedges’ *g*, *R meta* package) for compartmental qMT metrics and T1 was converted to R1 to ensure consistent directionality.

To assess longitudinal evolution of MT metrics in RRMS, longitudinal data (>1 time-point) were submitted to a mixed-model linear regression with mean MT as the dependent variable, time-point and brain region as fixed effects, and study as a random effect with within-study subgrouping as a nested factor (e.g. active lesions versus reactivated lesions, placebo versus treatment groups; *α* = 0.05; *lmer*, RStudio). Marginal means for each brain region were estimated (*ggeffects* R package). Follow-up sub-analyses were performed when *k* ≥ 3 for a given brain region, with time-point as a fixed effect and study as a random effect, with subgrouping as a nested factor [*α* = 0.05/(1 + *n* of sub-analyses)]. Formal sensitivity analysis was not considered applicable to these data.

### Qualitative assessment

Longitudinal change in MT, the relationship between MT and treatment, its association with disability and the dependence on the MT metric used were qualitatively assessed.

### Risk of bias

Risk of bias was determined qualitatively with Joanna Briggs Institute (JBI) Critical Appraisal Checklists,^33,34^ stratified by study type (case–control, randomized controlled trial, cross-sectional, cohort, case report, case series, or closest match of listed study designs). An overall appraisal was given to each study based on checklist criteria. Funnel plots were used to quantify publication bias across studies included in meta-analyses. The observational nature of the data being examined limited formal evaluation of overall certainty of evidence.

### Data availability

Extracted data may be provided upon reasonable request to the corresponding author.

## Results

### Systematic online literature search results

**Figure 1:**
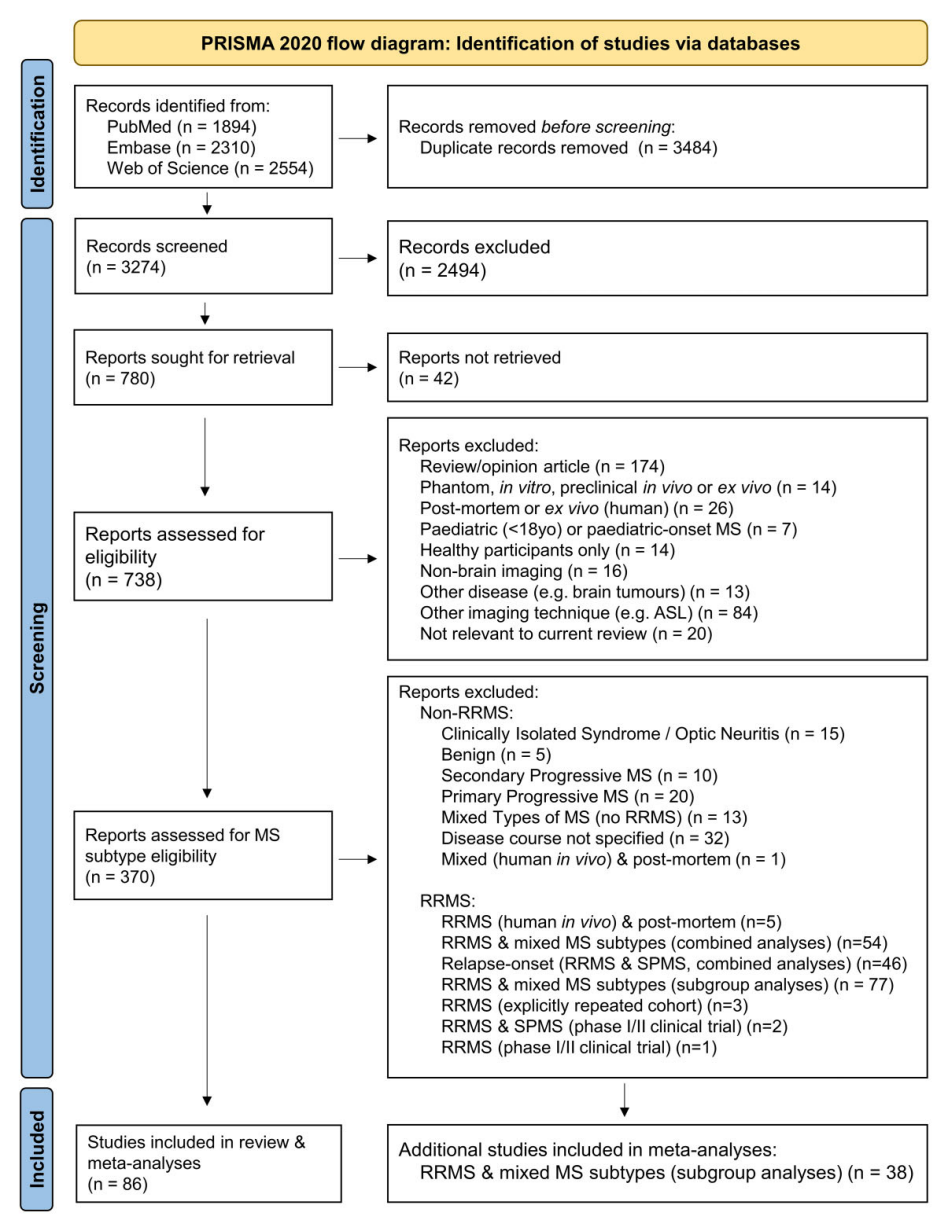
**PRISMA 2020 flow diagram for systematic review search process.** ASL, arterial spin labelling; MS, multiple sclerosis; PRISMA, Preferred Reporting Items for Systematic Reviews and Meta-Analyses; RRMS, relapsing-remitting MS; SPMS, secondary progressive MS. Adapted from: Page *et al.*^32^

Initial online database searches yielded 6758 results. Following the removal of duplicates, 3274 studies remained, which was reduced to 780 after abstract screening (Fig. 1). Full articles could not be retrieved for 42 studies and these were excluded. Of the remaining 738 articles, 368 studies met exclusion criteria (Fig. 1), leaving 370 articles for categorization by MS subtype.

As RRMS is the focus of this review, 96 studies that did not include patients with the relapsing-remitting MS subtype were excluded. The remaining selection (*k* = 274) was refined to 86 studies that only recruited participants with RRMS (and HCs, when included), and which form the foundations of this review. MTI data for RRMS patients from a further 38 studies, which had been excluded from the main review due to comprising mixed MS cohorts (as per the pre-defined study protocol) were additionally included in meta-analyses. An overview of excluded MS studies with mixed MS subtypes may be found in Supplementary Tables 1 and 2.

In adherence to our protocol, we did not include Phase I or II clinical trials. We nevertheless retrospectively examined these studies for potential inclusion in meta-analyses; however, these studies either did not include analysable MT data, or incorporated duplicate data from cohorts that had already been included in the existing analysis.

### Sample characteristics

An overview of sample size, sex ratio, age and study centre location is provided in Supplementary Table 3 for RRMS cohort studies (*k* = 86). Fifty-seven (44%) included a HC group. Disease duration and Expanded Disability Status Scale (EDSS) score for each study (when reported) is shown in Supplementary Table 4.

#### Sample size

The median number of patients with analysed MT data was 19 (range: 1–858, *k* = 86) compared with 14 HCs (range: 2–56, *k* = 57, Supplementary Table 3).

#### Sex

The median female-to-male ratio for analysed MT data was two for RRMS patients (*k* = 61) and 1.43 for HCs (*k* = 51, Supplementary Table 3).

#### Age

The mean age of people with RRMS was 37.15 years (5.63 SD, *k* = 77). Where mean age was only reported for recruited patients, this was still included; median age was not included. The mean age of HCs was 35.70 years (4.90 SD, *k* = 47) (Supplementary Table 3).

#### Location

The majority of studies were European (*k* = 41/86) or North American (*k* = 30), with a minority of Asian (*k* = 7, including Iran and Jordan) and international (*k* = 8) studies (or >3 test centres, Supplementary Table 3). The top three study locations were London (*k* = 8),^35–42^ Milan (*k* = 8)^43–50^ and Lausanne (*k* = 6).^51–56^

#### Disease duration

The mean disease duration across studies was 6.23 years (4.19 SD, range 0.2–20.8 years, *k* = 50/86 reported as mean, Supplementary Table 4).

#### Clinical disability

The majority of studies (*k* = 73/86) used EDSS as a measure of disability with median baseline score of 1.5 (*k* = 64, Supplementary Table 4).

Additional clinical correlates included the multiple sclerosis functional composite (MSFC, *k* = 11)^37–39,51,52,56–61^ or its subcomponents, i.e. the Paced Auditory Serial Addition Test (PASAT), nine-hole peg test (9HPT) or the Timed 25-Foot Walk (T25FW, *k* = 5),^53,62–65^ the Symbol-Digit Modalities Test (SDMT), Stroop test, Wechsler Abbreviated Scale of Intelligence, Adult Memory and Information Processing Battery, Hospital Anxiety and Depression Scale,^41^ Hamilton Depression and Anxiety Rating Scales, Mini-Mental State Examination and the Standard Raven Progressive Matrices.^65^

#### DMTs and steroid usage

Intra-study and inter-study heterogeneity were apparent in treatment with DMTs and steroids (Table 1 and Supplementary Table 5 for summaries; Supplementary Table 3 for detailed descriptions). Homogeneous DMTs were prescribed across the cohort in 11 studies (Supplementary Table 5); comprising fingolimod,^66^ dimethyl fumarate,^67,68^ subcutaneous interferon (IfN)-β1a,^58,69^ or IfN-β1b,^70–72^ intramuscular IfN-β1a^73,74^ and subcutaneous glatiramer acetate.^75^ Patients in four further studies were either untreated or received homogeneous DMTs which were IfN-α,^76^ IfN-β^38,39^ and glatiramer acetate.^77^

**Table 1:** Overview of use of DMTs for patients with relapsing-remitting MS in studies using MTI

DMTs	*k*	%	Citation
Dimethyl fumarate	4	4.7%	^67,68,89,90^
Dimethyl fumarate (delayed release)	2	2.3%	^91,92^
Fingolimod	10	11.6%	^49,51–56,66,89,90^
Natalizumab	5	5.8%	^35,49,89,90,93^
Glatiramer acetate	9	10.5%	^55,75,77,89,90,92–95^
Interferon-β (1a)	13	15.1%	^55,58,61,69,73,74,76,90,93,95–98^
Interferon-β (1b)/betaferon	5	5.8%	^55,70–72,93^
Interferon beta (unspecified)	8	9.3%	^38,39,51–54,56,94^
Pegylated interferon 1a	1	1.2%	^99^
Laquinomod	1	1.2%	^100^
Ocrelizumab	1	1.2%	^97^
Placebo	8	9.3%	^35,59,61,85–88^
**Steroids**	***k***	**%**	**Citation**
Methylprednisolone	2	2.3%	^71,72^
Unspecified	2	2.3%	^76,79^
None (for indicated time period)	26	30.2%	^35,40,41,43–46,48,50,53,54,57,62,65,66,68,73,81–85,94,101–103^
Data missing	56	65.1%	^11,36–39,42,47,49,51,52,55,56,58–61,63,64,67,69,70,74,75,77,78,80,86–93,95–100,104–119^

Studies may be duplicated where treatments were heterogeneous. Study-specific details are given in Supplementary Table 3. DMTs, disease-modifying therapies; *k*, number of studies.

Patients in five studies were treatment-naïve (and not receiving steroid treatment for a minimum of 14 days before imaging),^37,45,46,78,79^ and only the placebo arm of a clinical trial was included in one study.^80^ Eleven studies allowed steroid treatment for relapses or did not specify usage, but were otherwise treatment-naïve.^40,43,44,48,50,57,65,81–84^ Many studies did not report DMT or steroid usage (*k* = 28 and *k* = 56, Supplementary Table 5 and Table 1, respectively) or did not specify DMTs (*k* = 5).^59,85–88^ However, studies that reported steroid usage typically had a washout period of at least 10 days before MR imaging took place.

### MTI acquisition protocol parameters

**Figure 2:**
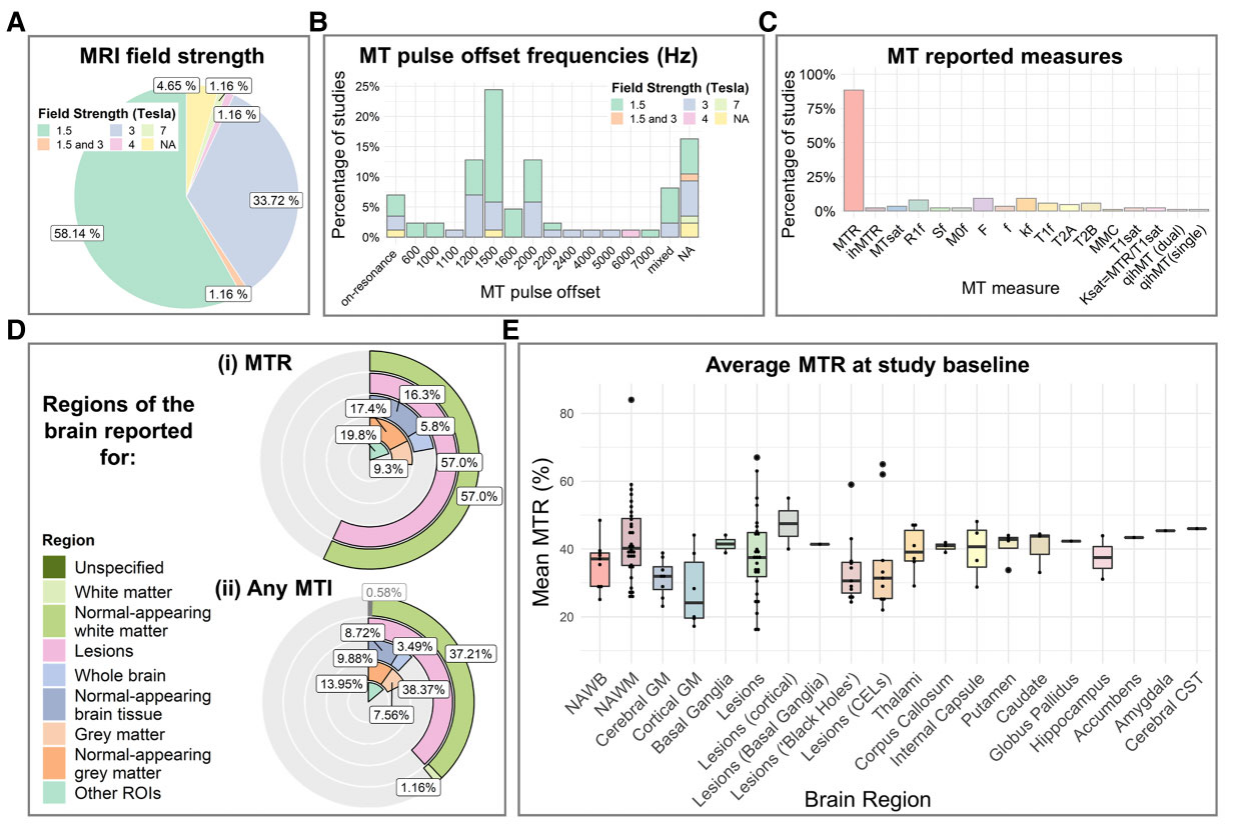
**MRI characteristics of studies which used MTI in relapsing-remitting MS (*k* = 86).** Plots summarise **A** field strength of the MR system, **B** pulse offset frequencies of the MT pulse, **C** MT metrics used across studies, **D** brain regions in which (i) MTR or (ii) any MTI metric was reported, and **E** the average MTR across brain regions at study baseline. CELs, contrast-enhancing lesions; CST, corticospinal tract; GM, grey matter; MMC, macromolecular content; MT, magnetization transfer; MTR, MT ratio; ihMTR, inhomogeneous MTR; MTsat, MT saturation; qihMT, quantitative inhomogeneous MT; NAWB, normal-appearing whole brain; NAWM, normal-appearing white matter; ROIs, regions of interest.

MTI protocols varied across studies (see Supplementary Results); there was heterogeneity in MR system field strength (Fig. 2A), acquisition sequence design, image contrast, image resolution and MT pulse design, including MT pulse offset frequency (Fig. 2B). Sequence parameter details were often, however, unreported.

### Quantitative measures of magnetization transfer

#### Metrics used

The most frequently used quantitative MT metric was MTR (*k* = 75, Fig. 2C and Supplementary Table 4).^35–63,65–76,78–95,97–103,107–110,112,113,115,117,119^ A small number of studies used MTsat (*k* = 3),^11,111,114^ ihMTR or quantitative ihMT (*k* = 2),^88,119^ or qMT (*k* = 16).^36,64,77,86,87,93,94,96,104–106,108,112,116,118,119^ qMT parameters included the R1_free_ (*k* = 7)^77,94,104–106,116,118^ or T1_free_ (*k* = 5)^36,86,87,96,112^ including under saturation (T1_sat_, *k* = 2),^86,108^ T2_free_ (*k* = 4)^77,94,116,118^ and T2_bound_ (*k* = 5),^36,77,94,116,118^ *k_f_* (*k* = 8)^64,77,87,96,105,106,112,116^ including under saturation (*k*_sat_, *k* = 2),^86,108^ the equilibrium magnetization of the ‘bound’ pool and the non-ideal inversion of the ‘free’ pool signal (M0f and Sf, respectively, *k* = 2),^105,106^ *f* (*k* = 3),^36,94,118^ and *F* (*k* = 2).^64,77,93,94,104–106,116^

#### MT values across the brain

Studies varied as to the brain tissues in which MT was evaluated (Fig. 2D and Supplementary Table 4). Metrics were most often investigated in WM (*k* = 55)^11,35–38,40,43,45,46,48,51–55,58,60,64,66–68,70–72,74,77–79,81–90,93,94,96–98,100,102,105,106,108,110,112,114,115,117–119^ and lesions (*k* = 58),^11,35,36,42,43,45,46,49–54,58,59,61,65–75,77,79,80,82–88,90,91,93–98,100–102,105–107,110,112,114–116,118,119^ followed by grey matter (*k* = 30),^11,36–38,40,44,48,51–55,57,60,64,68,70,74,82,85,89,97,100–102,105,106,109,116,118^ whole brain (*k* = 19)^11,43,47,50,59,61,65,69,74–76,80,82,91,92,99,100,102–104,113^ and specific regions of interest (ROIs) (*k* = 22).^35,39–41,43,51–53,56,62,63,72,85,88,97,101,105,106,111,116,118,119^ However, the definition of tissue categories varied. A distinction was often (but not always) made between ‘normal-appearing’ tissue and lesional tissue. Certain studies sub-divided tissue type into lobes (e.g. frontal WM) or ROIs (e.g. deep versus cortical grey matter).

#### MTR in RRMS and HCs

##### Meta-analysis

**Figure 3:**
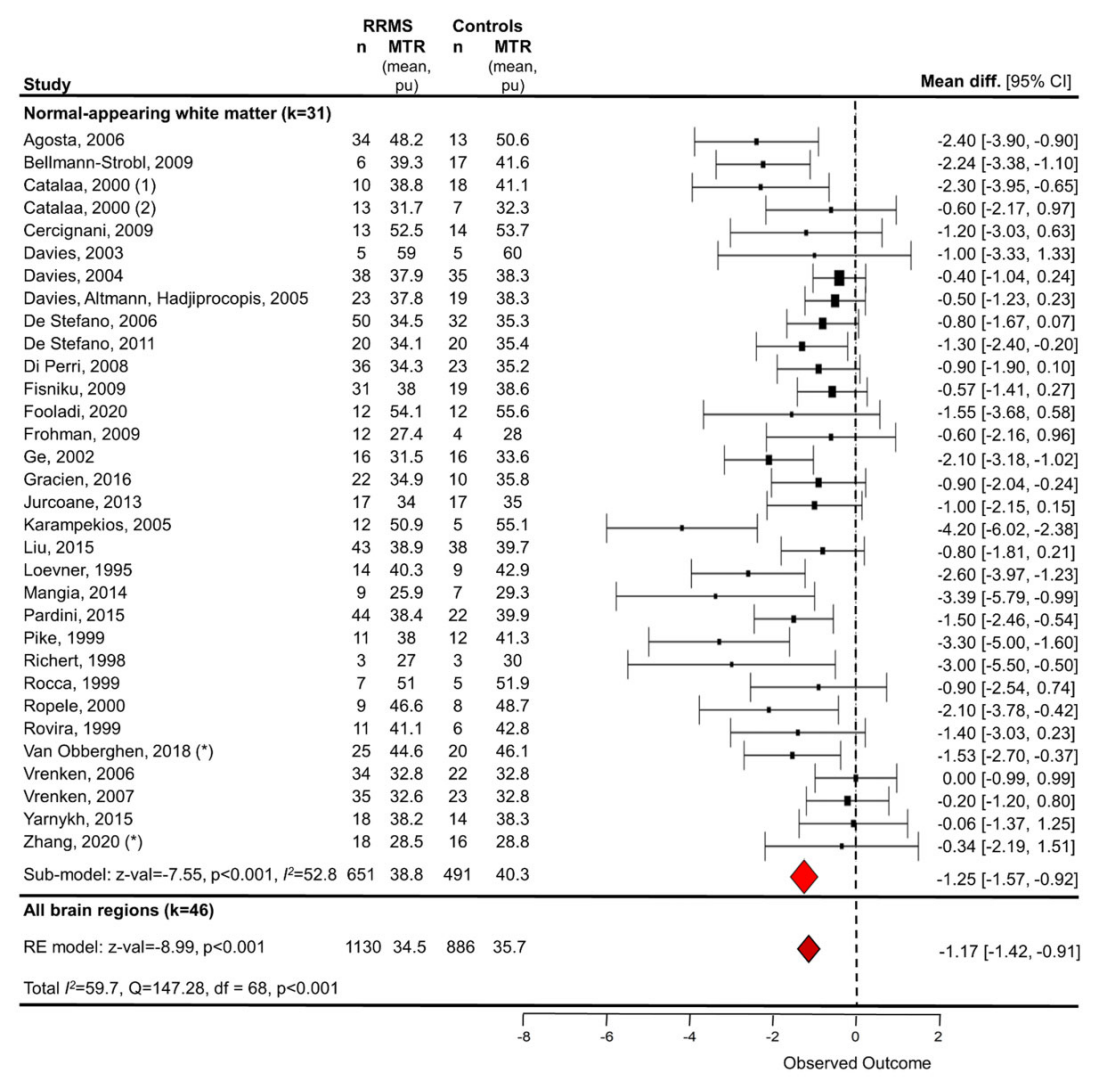
**Random-effects meta-analysis of the difference in mean MTR in between relapsing-remitting MS patients and control subjects in NAWM and all brain tissue types**. Study baseline data were used. One study (Catalaa^78^) was included twice as separate protocols and cohorts were used. A random-effects model with brain region as a nested factor showed that mean MTR was 1.17 per cent units [*z*-value = −8.99, *P* < 0.001, 46 studies (including grey matter and whole brain studies in Fig. 4), 1130 RRMS/886 HC] lower for people with RRMS than HCs across all brain tissue types. A random-effects model for NAWM alone showed that mean MTR was 1.25 per cent units (*z*-value = −7.55, *P* < 0.001, 31 studies/*n* = 32; 651 RRMS/491 HC) lower for people with RRMS than HCs. NAWM, normal-appearing white matter; RE, random-effects; RRMS, relapsing-remitting multiple sclerosis. *Averaged over sub-regions.

Studies that compared MTR cross-sectionally between RRMS patients and HCs (*k* = 46 with available data, *n* = 1130 RRMS patients/886 HC) were submitted to a random-effects meta-analysis, with brain region as a nested factor. Irrespective of brain region, MTR for RRMS patients was on average 1.17 per cent units [95% confidence interval (CI) −1.42 pu to −0.91 pu] lower than controls (*z*-value: −8.99, *P* < 0.001, Fig. 3). Between-study heterogeneity was high (total *I*^2^ = 59.7%).

##### Whole-brain MTR

**Figure 4:**
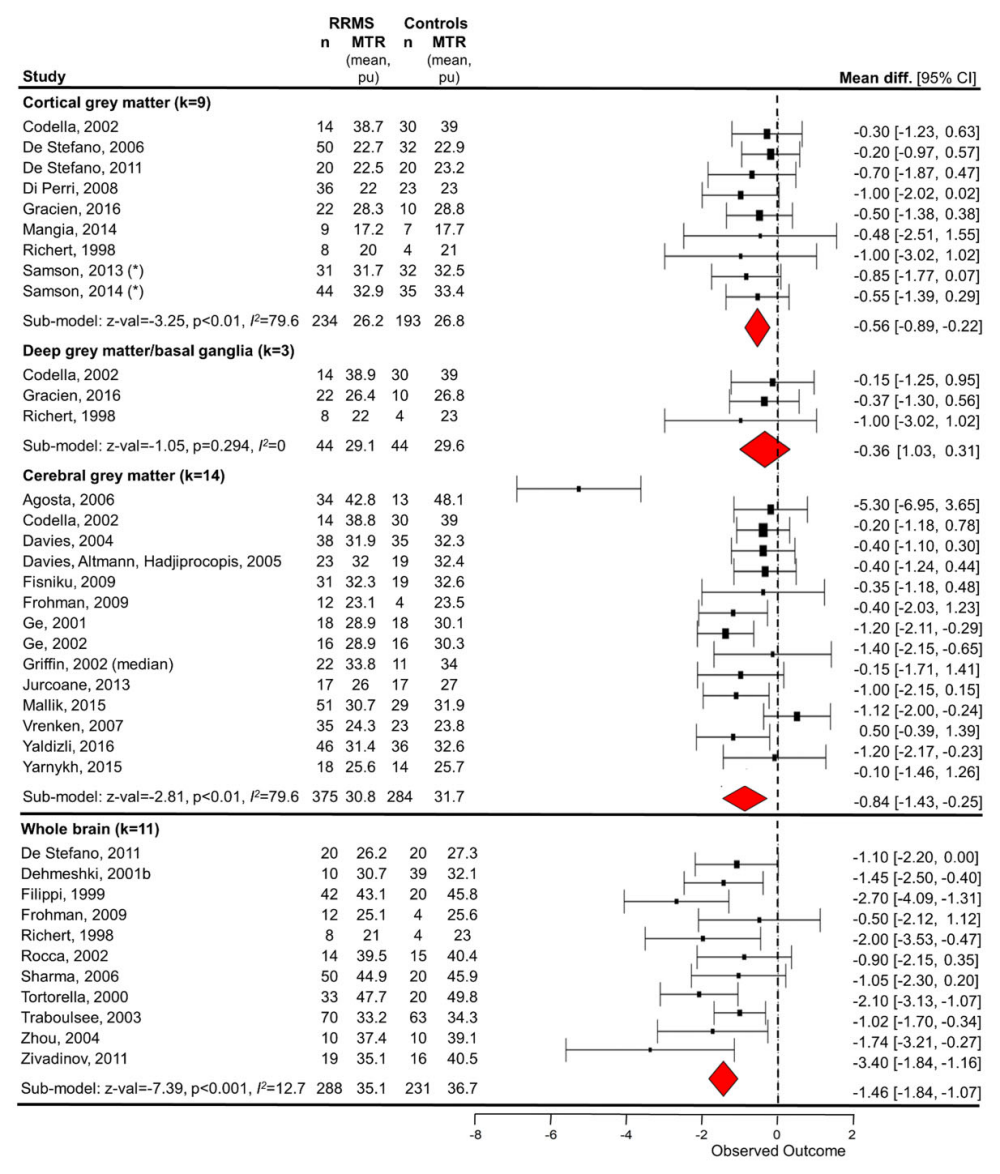
**Random-effects meta-analysis of the difference in mean MTR between relapsing-remitting MS patients and control subjects in grey matter and whole brain**. Random-effects models of study baseline data showed that mean MTR was lower for people with RRMS than HCs in whole brain (mean difference −1.46, *z* = −7.39, *P* < 0.001 uncorrected, 11 studies, 288 RRMS/231 HC), cortical grey matter (−0.56, *z*-value = −3.25, *P* = 0.001, nine studies, 234 RRMS/193 HC), and cerebral grey matter (−0.84, *z*-value = −2.81, *P*= 0.005, 14 studies, 375 RRMS/284 HC), but not deep grey matter/basal ganglia (−0.36, *z*-value = −1.05, *P* = 0.294, three studies, 44 RRMS/44 HC). See Fig. 3 for estimate across all brain tissue types, including NAWM. GM, grey matter; NAWM, normal-appearing white matter; RE, random-effects; RRMS, relapsing-remitting multiple sclerosis; WB, whole brain. *Averaged over sub-regions.

Whole-brain MTR was measured in 19 studies (Supplementary Table 4 and Fig. 2D).^43,47,50,59,61,65,69,74–76,80,82,91,92,99,100,102,103,113^ Average MTR in whole brain (*k* = 9) was 35.58%^47,50,59,65,74,75,80,82,91^ with wide inter-study variance (range: 25.1%^82^ to 48.44%,^59^ Fig. 2E). Subgroup meta-analysis showed that whole-brain MTR was significantly lower for patients than HCs with an absolute mean difference of −1.46 pu (95% CI −1.84 to −1.07 pu) (*P* < 0.001, *z*-value: −7.39, Fig. 4 subgroup, *k* = 11 with sufficient reported data, *n* = 288 RRMS/231 HC) with low between-study heterogeneity (*I*^2^ = 12.7%).

##### Normal-appearing WM MTR

MTR of WM was investigated in a large number of studies (*k* = 48/86, Fig. 2D and Supplementary Table 4).^35–38,40,42,43,45,46,48,51–55,58,60,66–68,70–72,74,78,79,81–90,94,96–98,100,102,108,110,112,115,117,119^ Typically, WM was defined as whole-brain normal-appearing WM (NAWM), with some exceptions such as ROIs of NAWM contra-lateral to lesions of similar size,^66,68,96^ ‘dirty-appearing’ WM^79,112^ and NAWM sub-regions^36,40,42,45,46,81,87,88,117,119^ (e.g. lobar WM,^51,52,67,115^ NAWM close to cortical grey matter,^43^ perilesional NAWM^35,96,110^). The mean NAWM MTR across studies was 69% (*k* = 32)^36–38,42,43,45,46,48,58,60,66–68,70–72,74,78,79,82,84–89,94,98,102,110,112,119^ (range: 25.95%^60^ to 84%,^67^ Fig. 2E).

Overall, NAWM MTR was lower in RRMS patients compared with HCs,^37,39,40,43,58,60,70,78,81,83,86–90,112^ although some studies found no difference.^36,51,53,54,82,84,94,119^ One study reported lower MTR in controls than patients.^97^ Random-effects subgroup meta-analysis (Fig. 3) showed MTR of NAWM in RRMS was significantly lower than controls, with an absolute mean difference of −1.25 pu (95% CI −1.57 to −0.92) (*z*-value −7.55, *P* < 0.001, *k* = 31 with sufficient data, *n* = 651 RRMS/491 HC) and considerable between-study heterogeneity (*I*^2^ = 52.8%).

##### Grey matter MTR

Twenty-three studies investigated grey matter MTR (Fig. 2D and Supplementary Table 4).^36–38,40,44,48,51,53–55,57,60,68,70,74,82,85,89,97,100–102,109^ Mean cerebral normal-appearing grey matter (NAGM) MTR was 31.5% (*k* = 9),^37,38,40,44,48,74,82,102,109^ and consistently lower than NAWM MTR^38,40,102^ with a wide range (Fig. 2E). Cortical NAGM MTR, for example, was 2.9 per cent units lower when using a balanced steady-state free precession sequence compared with a gradient echo sequence within the same cohort.^85^

Random-effects subgroup meta-analyses showed a significant difference for cerebral and cortical grey matter (Fig. 4, mean difference −0.84 and −0.56 pu, *z*-value −2.81 and −3.25, *k* = 14 and 9, *n* = 375/284 and 234/193 RRMS/HC, respectively, *P* < 0.01 for both) but not deep grey matter (mean difference −0.36, *z*-value: −1.05, *P* = 0.294, *k* = 3, *n* = 44 RRMS/44 HC). However, other studies (which did not report effect sizes) did not find between-group differences in MTR within cerebral^36,54^ or cortical NAGM,^51,53^ or within the basal ganglia.^51,53^ Moreover, sub-regional variation was reported. For example, grey matter MTR in the parieto-occipital lobes, but not other regions, was lower for patients than controls in one study,^40^ and voxelwise differences in the left posterior cingulate cortex, right orbitofrontal cortex, bilateral insula and lenticular nuclei were noted elsewhere between patients and controls.^57^

##### Lesion MTR

Forty-nine studies measured MTR in lesions (Fig. 2D and Supplementary Table 4).^35,36,42,43,45,46,49–54, 58,59,61,65–75,79,80,82–88,90,91,93,95,97,98,100–102,107,110,112,115,119^ MTR was nearly always lower in WM lesions than in NAWM (*k* = 23, Fig. 2E),^36,42,43,53,60,66,67,70–72,79,83–86,88,94,96–98,110,112,115^ ‘dirty-appearing’ WM^79^ and HC WM (*k* = 4).^53,58,84,119^ Cortical lesion MTR was also lower than cortical NAGM.^85^ However, there was some regional heterogeneity. WM lesion MTR (and ihMTR) was not significantly lower than NAWM in the corpus callosum^88^ nor when several NAWM ROIs were combined.^119^

There was clear variation in MTR across lesions (Fig. 2E), partially dependent on lesion characteristics,^53,107^ which varied across the literature. In particular, MTR in T1-w ‘black holes’ was lower than in T1-w-isointense, T2-w visible lesions^67,102^ although not always significantly.^42^ There was not typically a significant difference between MTR in contrast-enhancing lesions (CELs) such as nodular-enhancing CELs, and non-CELs,^107^ ‘pure T2-w lesions’ or T1 ‘black holes’.^67^ However, ring-enhancing CELs showed lower MTR than densely enhancing^87^ or nodular-enhancing CELs.^84^ In addition, interdependency between lesion volume and MTR was reported,^43,53^ although results are mixed.^80^

##### MTR in other sub-regions

Seventeen studies measured MTR in other sub-regions of the brain (Fig. 2D and Supplementary Table 4)^35,39–41,43,51–53,56,62,63,72,85,88,97,101,119^ including the thalami,^39–41,51,53,85,88,101,119^ putamen,^40,51,53,85,88,101^ caudate nuclei,^40,51,53,85,101^ corpus callosum,^40,63,88,119^ internal capsule,^40,43,88,119^ globus pallidus,^51,53,85,101^ cerebellum,^52,56^ hippocampi,^41,85^ cerebral corticospinal tract,^62^ accumbens,^85^ amygdala,^85^ cingulate cortex^41^ and parietal cortex.^41^

A random-effects meta-analysis with brain sub-region as a nested factor showed no significant difference in baseline MTR between patients and controls [absolute mean difference −3.31 pu (95% CI −8.65 to 2.03), *z*-value = −1.23, *P* = 0.215, *k* = 7, *n* = 161 RRMS/142 HC, Supplementary Fig. 1]. Although between-study variance was low (*I*^2^ = 0.07%), total model variance was high (*I*^2^ = 98.9%) due to high variation in brain region (Fig. 2E).

Since the number of studies examining MTR for most individual brain regions was low (*k* < 3), follow-up subgroup random-effects meta-analyses were only performed for the thalamus (*k* = 6) and putamen (*k* = 3). There was no significant difference in baseline thalamic MTR between RRMS patients and HCs [mean difference −3.97 pu (95% CI −10.07 to 2.12), *z*-value = −1.28, *P* = 0.202, *n* = 132 RRMS/113 HC, Supplementary Fig. 1] and high between-study variance (*I*^2^ = 99.2%). One additional study also found no difference in thalamic MTR between patients and controls (no effect size reported).^51^ Similarly, for the putamen, there was no difference between patients and controls [mean difference −5.77 pu (−17.10 to 5.56), *z*-value = −1.0, *P* = 0.318, *n* = 77 RRMS/61 HC] and heterogeneity was high (*I*^2^ = 99.6%). High between-study heterogeneity may be explained by differences in MT sequences used.^85^

#### Longitudinal MTR change and therapeutic response

Fourteen studies (*n* = 563 RRMS) assessed longitudinal change in mean MTR in one or more brain regions, with a maximum of 3 years follow-up. A linear mixed-model revealed that time did not have a significant effect on MTR when all brain regions were considered [*β* = 0.12 (−0.56 to 0.80), *t*-value = 0.35, *P* = 0.724, Supplementary Table 6 and Fig. 2].

##### Longitudinal change in whole-brain MTR

Ten studies examined the longitudinal evolution of whole-brain MTR^59,61,69,74–76,80,91,92,99,100^ of which five reported sufficient data to estimate longitudinal change in normal-appearing brain tissue (NABT) MTR.^59,74,75,80,91^ A linear mixed-model showed that time did not significantly predict NABT MTR [*β* = −0.117 (−0.21 to −0.02), *t*-value = −2.65, *P* = 0.019, *n* = 278 RRMS, Supplementary Table 7].

Nevertheless, individual studies reported small (e.g. <1% absolute change over 2 years^47^) but significant longitudinal decline in whole-brain MTR.^59,76^ A slower (non-significant) MTR decline (e.g. ∼0.02% every 2 months over 14 months^80^) and inter-subject variation were also reported. ^69,76^ Additionally, longitudinal stagnation or increase in MTR with treatment compared with longitudinal decreases in MTR in placebo arms was evident in large, placebo-controlled cohorts over 2 years,^91,100^ suggesting MTR as a putative therapeutic endpoint. However, one study reported no deterioration in whole-brain MTR with glatiramer acetate treatment but lacked validation against a placebo arm.^75^

##### Longitudinal change in NAWM MTR

Sixteen studies examined the longitudinal evolution of NAWM MTR.^38,45,46,53,54,58,66,71,74,78,83,84,96,98,100,120^ Eight studies (*n* = 100 RRMS) reported appropriate data for a linear mixed-model to assess longitudinal change; NAWM did not change significantly over time [*β* = 0.037 (−0.14 to 0.22), *t*-value = 0.41, *P* = 0.68, Supplementary Table 8].^45,46,58,66,74,84,96,120^

In studies that reported a significant change over time, and in line with a previous report,^98^ absolute change in NAWM MTR was small (<1.5% up to 36 months) with reported estimates of an annual decline of 0.1% in early RRMS, possibly preceding clinical onset by years.^38^ However, others found no change in NAWM MTR over 2 years in an early MS cohort with minimal disability, after controlling for age and gender.^53^ Alternatives to the arithmetic mean such as histogram peak location may, nevertheless, reveal changes over 12–32 months.^78^

##### Longitudinal change in grey matter MTR

A linear mixed-model of all brain regions suggests no effect of time on NAGM MTR but there were insufficient data for follow-up analyses (see ‘Longitudinal MTR change and therapeutic response’ section). In the literature, however, MTR in grey matter decreases gradually (∼0.18 pu annually, compared with 0.01 pu in controls),^38^ although perhaps faster than NAWM MTR in RRMS.^38^ However, over 2 years, such a gradual decline is not statistically significant.^53^ The longitudinal rate of grey matter change is unaffected by anti-phospholipid antibody (APLA) status,^74^ or treatment with IfN-β^38^ or laquinomod,^100^ although the latter may slow decline initially.

##### Longitudinal change in sub-regional MTR

There was no evidence of longitudinal change in MTR when all brain regions were considered (see ‘Longitudinal MTR change and therapeutic response’ section). Since there were few studies examining each brain sub-region (Supplementary Fig. 2), no further meta-analyses of longitudinal change in MTR within brain sub-regions were constructed. However, no significant longitudinal change in MTR has been found in the thalamus, putamen, pallidum or caudate over 2 years.^53^ Separately, despite a significant change in thalamic MTR (−0.13 pu/year) over 2 years, this was not significantly different from the rate of change in control thalamic MTR,^39^ and did not differ between those patients who were or were not treated with IfN-β.

##### Longitudinal change in lesion MTR

A linear mixed-model showed that lesion MTR did not change significantly longitudinally [*β* = 0.255 (−0.52 to 1.02), *t*-value = 0.67, *P* = 0.51, *k* = 11, *n* = 223 RRMS, Supplementary Table 9].^45,46,59,66,74,75,80,84,96,98,120^ However, MTR longitudinal evolution depends on lesion characteristics^53^ and may be subtle^69^ (Supplementary Figs 2 and 3). MTR of active CELs varies from month-to-month before and after enhancement,^45,46,71,83,93,96^ while MTR of GM lesions,^53^ ‘slowly expanding’ lesions,^49^ T1-w hypointense^75^ and T2-w hyperintense^75,80^ lesions may remain relatively stable over several years, irrespective of relapses.^80^

Increases in lesion MTR may also occur,^84^ such as within non-expanding lesions, although this may be accompanied by changes in T1^49^ and/or lesion load^61^. MTR increases may be seen with treatment (e.g. fingolimod^66^ over 2 years) although not always (e.g. laquinomod^100^). Steroids can increase CEL MTR^46,71^ although certain DMTs, including delayed-release dimethyl fumarate^91^ or IfN β-1b^71,73^ do not appear to alter CEL MTR. Furthermore, CELs do not tend to recover to NAWM MTR values,^46,72,98^ and their longitudinal evolution may be predicted by the change in MTR of the first-month post-enhancement.^46^ MTR in reactivated CELs also may deviate from NAWM MTR to a greater extent than new CELs.^96^

MTR fluctuations in lesions have been partially ascribed to low reproducibility, changes in interstitial water due to acute inflammation, or perhaps remyelination.^68^ Yet, when mixed lesion types are considered, a longitudinal global MTR decrease is typical.^53,54^

#### Clinical correlates of MTR

**Figure 5:**
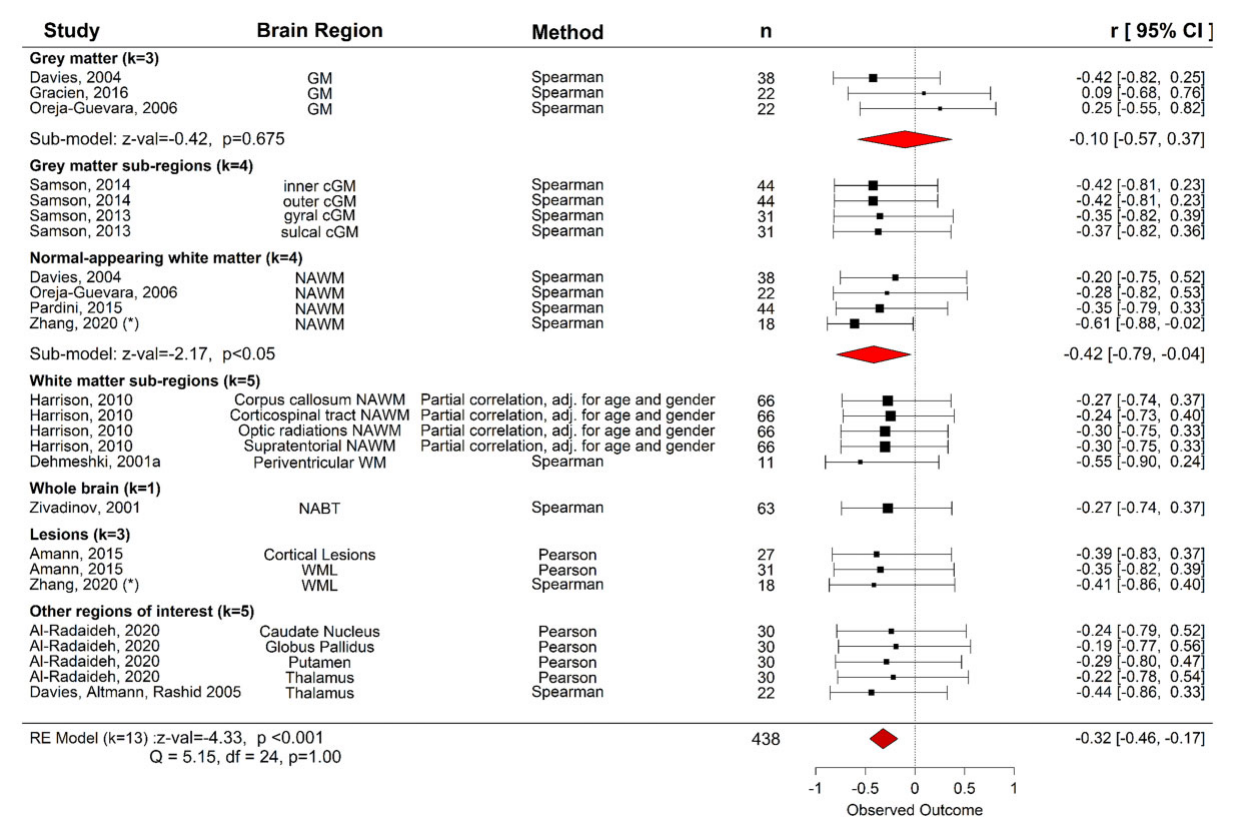
**Meta-analysis of association between MTR and clinical disability in relapsing-remitting MS.** Clinical disability was defined as EDSS score. A multi-level random-effects model with brain region as a nested factor within each study showed a significant negative association (*r* = −0.32, *z*-value = −4.33, *P* < 0.001, 13 studies, 438 RRMS) between MTR and EDSS across all brain regions. Studies which did not report a correlation coefficient were not included. Random-effects sub-analyses showed a significant correlation between EDSS and NAWM MTR (*r* = −0.42, *z*-value = −2.17, *P* = 0.030, four studies, 122 RRMS), and not grey matter (*r* = −0.10, *z*-value = −0.42, *P* = 0.675, three studies, 82 RRMS). Sub-analyses were not performed when the number of studies, *k* < 3. *MTR values were averaged over sub-regions of NAWM. GM, grey matter; NABT, normal-appearing brain tissue; NAWM, normal-appearing white matter; WML, white matter lesions; RE, random effects; CI, confidence interval.

Thirteen studies reported correlation coefficients between MTR and EDSS permitting a meta-analysis (with the brain region as a nested factor) to be performed. There was a significant negative association between EDSS and MTR across all brain regions; *r* = −0.32 [95% CI −0.46 to −0.17] (*z*-value = −4.33, *P* < 0.001, *k* = 13, *n* = 438, Fig. 5) and between-study heterogeneity was low (total *I*^2^ = 0%). Across individual studies, sub-regional results were mixed but in general, suggest that there is no association between EDSS and MTR.^85,88^

##### Whole-brain MTR and clinical correlates

In terms of whole-brain MTR clinical correlates, there is some evidence that NABT MTR correlates with EDSS^65^ (Fig. 5) but not retinal nerve fibre layer (RNFL) thickness or low letter contrast acuity.^82^ NABT MTR may predict longitudinal memory decline and, in combination with brain parenchymal fraction and 2-year change in ventricular fraction, information processing speed over 7 years.^59^ No such association was found between NABT MTR and verbal fluency.^59^ However, this study was limited by the lack of comparative longitudinal control data. Furthermore, longitudinal evolution of NABT MTR does not appear to depend on APLA status of patients.^74^

##### NAWM MTR and clinical correlates

Many studies examined the relationship between clinical disability and NAWM MTR (Supplementary Table 4), yet only three studies reported effect sizes. A subgroup meta-analysis for NAWM showed a negative association between EDSS and NAWM MTR [*P* < 0.05, *r* = −0.42 (95% CI −0.79 to −0.04), *n* = 122 RRMS, Fig. 5] with low between-study variance (*I*^2^ = 0%). However, the small number of studies (*k* = 4) limits the generalisability of this finding, particularly given under-reporting of non-significant effect sizes. Indeed, all studies (*k* = 10/86) which examined the association between NAWM MTR and EDSS found no association,^37,38,58,60,75,78,85,115,119^ although one study reported a significant correlation between baseline NAWM MTR and change in EDSS over 18 months (but not baseline EDSS).^48^

Evidence of relationships between NAWM MTR and other clinical measures was mixed. For example, NAWM MTR was associated with MSFC *z*-score at 24-month follow-up but not baseline^58^ while, separately, there was no relationship between MSFC *z*-scores and NAWM MTR^60^ or 2-year change in NAWM MTR.^38^ Associations may also be region- and model-dependent; for example, temporal lobe MTR was one of several significant predictors of MSFC and SDMT (an attention test) scores, independently, in regression models.^51^

In terms of other biomarker correlates, WM MTR was weakly associated with serum neurofilament—a marker of neuronal injury—in RRMS (although not in control subjects), adding to evidence validating MT imaging as a biomarker of myelin integrity.^55^ NAWM MTR does not however appear to be related to RNFL thickness or low contrast letter acuity.^82^

##### Grey matter MTR and disability

Eight studies examined the relationship between grey matter MTR and EDSS (Supplementary Table 4) with some demonstrating significant associations^37,109^ and others finding no such relationship.^38,57,60,85,89^ One study found an association between baseline grey matter MTR and change in EDSS, but not baseline EDSS.^48^ A follow-up subgroup random-effects meta-analysis showed no significant association between-study baseline (cortical or cerebral) grey matter MTR and EDSS [*P* = 0.675, *r* = −0.10 (95% CI −0.57 to 0.37), *n* = 82 RRMS, Fig. 5] and low between-study heterogeneity (*I*^2^ = 0%), but the number of studies was small (*k* = 3).

Four studies examined the relationship between grey matter MTR and the MSFC.^37,38,57,60^ MSFC *z*-score did not correlate with cerebral NAGM,^37^ cortical NAGM^60^ or voxels of NAGM for which the MTR differed from controls.^57^ Furthermore, neither change in MSFC nor its cognitive component correlated with change in MTR in NAGM over 2 years.^38^

Regarding other clinical variables, NAGM MTR was significantly correlated with age^85^ as well as RNFL thickness of eyes affected by optic neuritis.^82^ Female subjects may also have higher NAGM MTR^37^ although this was not a consistent finding.^85^ In addition, NAGM MTR correlates with T1 and myelin water fraction.^97^ On the other hand, grey matter MTR did not correlate with low contrast letter acuity,^82^ RNFL of eyes unaffected by optic neuritis,^82^ serum neurofilament levels,^55^ immune cell brain-derived neurotrophic factor (BDNF) secretion,^102^ APLA status,^74^ fatigue^44^ or disease duration.^37,57,85^ Change in NAGM MTR was not associated with relapse rate, baseline T2 lesion volume or change in T2 lesion volume over 2 years^38^ nor APLA status over 3 years.^74^

##### MTR in other sub-regions and disability

MTR within other sub-regions such as the internal capsule,^43,88^ cerebral corticospinal tract,^62^ caudate, pallidum, putamen, accumbens, hippocampus and amygdala^85^ and corpus callosum^88^ was not associated with EDSS. There was a negative association between thalamic MTR and EDSS averaged over 2 years,^39^ although 2-year change in thalamic MTR was not associated with EDSS at follow-up,^39^ possibly reflecting a lack of change in thalamic MTR over 2 years.^53^

Regarding other clinical correlates, no relationship was found between thalamic MTR or rate of change of MTR over 2 years and MSFC.^39^ Nevertheless, the walk component of the MSFC was negatively associated with thalamic MTR.^39^ In the cerebral corticospinal tract, MTR was associated with walk velocity and Two Minute Walk Test but not Pyramidal Functional Systems Score, gender or symptom duration, but perhaps slightly dependent on age.^62^ MTR of the corpus callosum was positively associated with PASAT (the cognitive component of the MSFC) score, although possibly mediated by lesion load.^63^ Cognitively impaired RRMS patients may also have marginally reduced MTR in the corpus callosum compared with unimpaired patients.^63^ There may be an influence of age on MTR in the basal ganglia, thalamus and hippocampus.^85^ Finally, MTR in an area of the cerebellum thought to be involved in movement trajectories was associated with performance on the MSFC arm component.^56^

##### Clinical and other imaging correlates of lesion MTR

In lesions, any relationship between clinical disability and MTR is at most weak.^85,119,35,51,58,85,101,115^ Only two studies reported a correlation coefficient (Fig. 5) for an association with EDSS and hence a meta-analysis was not performed for lesion MTR alone.

This relationship may depend on lesion type, characteristics^52^ and location.^85^ For example, cortical, but not WM, lesion MTR was related to EDSS, after adjusting for demographic factors.^85^ Furthermore, when lesions were grouped according to their inflammatory and neurodegenerative characteristics, lesions with low MTR were found to predict attention deficits (SDMT) and general disability (MSFC), when combined with age and depression score.^52^

The timescale of the study, disease duration^85^ and treatment of confounding variables may affect the strength of association. A longitudinal relationship between MTR in lesions and clinical disability developed with longer disease duration in one study when not present at baseline.^58^ Lesion MTR, when combined with T2-w lesion and NAWM measures, was also related to longitudinal change in deambulation (MSFC T25FW).^53^ However, baseline T2-w lesion MTR was not a significant predictor of change in memory, verbal fluency or information processing speed over 7 years.^59^

More generally, the association between MTR and clinical disability may depend on which clinical measure(s) are used. For example, lesion MTR was not significantly different between cognitively impaired and unimpaired patients, when assessed by an extensive battery of neuropsychological tests.^65^ Similarly, MTR within (mixed-type) lesions did not correlate with motor tasks (finger tapping rate or 9HPT),^50^ and was not a significant predictor in regression models to predict general clinical disability (MSFC), attention (SDMT) or fatigue (Fatigue Scale for Motor and Cognitive functions).^51^

Some studies indicate associations between MTR as a measure of myelin integrity and other imaging markers of disease in MS. Weak evidence suggests that the uptake of radiotracer ^18^F-PBR111, which binds to the 18-kD translocator protein, is greater in around 60% of T2-w fluid-attenuated inversion recovery (FLAIR) hyperintense regions compared with non-lesional regions with high MTR.^35^ Higher uptake of ^18^F-PBR111 is suggestive of a pathological increase in macrophages and microglia. Single-subject MR spectroscopy has shown elevated choline and lactate/lipids suggestive of demyelination and injury to cell membranes, alongside decreases in N-acetyl compounds, creatine and myoinositol indicating axonal loss and increased glial cell infiltration, and decreased MTR compared with NAWM in a tumefactive CEL.^72^ MTR in lesions is strongly associated with other imaging metrics such as MMC,^93^ and *k_f_*^87,93,112^ and, to a lesser extent, quantitative T1^93,97,112^ and myelin water fraction.^97^ Lesion MTR is negatively correlated with relative activation on functional MRI in motor areas suggestive of functional adaptations to loss of myelin integrity, although perhaps confounded by lesion volume.^50^ MTR correlates weakly with diffusion-weighted imaging metrics including fractional anisotropy^110^ in large T2-w lesions and mean diffusivity^115^ in chronic lesions, but not significantly with susceptibility-weighted phase imaging values, despite a negative trend.^115^ Additionally, T2-w and T1-w ‘black hole’ lesion volume, as well as 2-year change in T2-w lesion volume may predict lesion MTR 13 years later, although uncorrected for baseline lesion MTR.^61^

Nevertheless, as a general trend across the RRMS literature, MTR within lesions does not tend to correlate with other disease biomarkers. T2-w lesion MTR is not significantly associated with age,^85,115^ time since diagnosis,^101^ visual contrast acuity or RNFL thickness,^82^ immune cell BDNF secretion,^102^ or APLA status (±).^74^ MTR in CELs was not associated with anti-CD3 plus anti-CD28 stimulated BDNF secretion, despite a negative trend.^102^ MTR in T1-w ‘black holes’ is not associated with RNFL thickness or visual contrast acuity.^82^ There is some evidence that APLA+ patients show greater reduction in MTR in T1 ‘black holes’ compared with APLA-patients over 3 years, but this may be driven by lesion volume changes.^74^ Evidence for associations between lesion MTR and disease duration or gender is mixed, and may depend upon acquisition parameters and lesion type.^85,115^

#### Magnetization transfer saturation

Three studies used MTsat (Fig. 2C),^11,111,114^ beginning with Helms *et al*.^11^ who showed that, on a whole-brain histogram, the WM MTsat mode appeared visually reduced in a RRMS patient compared with controls. Furthermore, compared with NAWM, MTsat in a CEL and non-enhancing lesions was visually lower on a parametric map.^11^

Saccenti *et al*.^114^ confirmed that MTsat was significantly lower in WM ‘plaques’ and periplaques than NAWM. Yet, MTsat did not correlate with EDSS or disease duration in plaque, periplaque or NAWM ROIs.^114^ MTsat may additionally correlate with radial diffusivity, T1w/T2w ratio and synthetic MR-derived myelin volume fraction, although this was stronger in plaques than NAWM.^114^

Finally, Kamagata *et al*.^111^ used MTsat as a surrogate for myelin volume fraction to calculate the tract-averaged MR *g*-ratio within WM in a small RRMS cohort.^111^ The *g*-ratio was increased (indicating myelin degradation and/or axonal loss) compared with HCs, in motor somatosensory, visual and limbic regions. Subnetwork *g*-ratio strongly negatively correlated with WM lesion volume, but not with disease duration or EDSS, although the latter was correlated with *g*-ratio connectome nodal strength mainly in motor, visual and limbic regions.

#### Inhomogeneous MTR

Two studies employed ihMTR as a measure of myelin status in RRMS.^88,119^ ihMTR was reduced in lesions and NAWM compared with control WM, and reduced in lesions compared with NAWM.^119^ Within sub-regions, single-slice ihMTR was lower for patients in the thalamus, frontal, temporal and occipital lobes compared with controls, but not different in the corpus callosum, internal capsule or putamen.^88^ ihMTR varied across WM tracts, but was highest in the internal and external capsule and lowest in the genu of the corpus callosum.^88,119^ ihMTR in WM lesions, but not NAWM, was negatively associated with EDSS.^119^ However, when sub-regions were considered, EDSS was significantly associated with ihMTR (but not MTR) in frontal and temporal NAWM, the corpus callosum, internal capsule and the thalami.^88^

#### Quantitative magnetization transfer

qMT metrics examined varied across studies (see ‘Quantitative measures of magnetization transfer: metrics used’ section). Sled and Pike^116^ first modelled the compartmental MT signal in RRMS in two lesions on a single-slice proton density-weighted image for a RRMS patient. Compared with frontal WM, lesions had reduced *k_f_*, *F*, R1_free_ and T2_bound_ and increased T2_free_. Parameter estimates were higher for the newer lesion compared with the older lesion for *k_f_*, *F* and R1_free_, but lower for T2_free_ and T2_bound_. Indeed, other studies also show lower *k_f_* and *k*_sat_ lesions than NAWM and HC WM, while T1_free_ and T1_sat_ present the inverse pattern.^86,87,112^ Up to 4 months before the appearance of new or reactivating CELs, *k_f_* may even decrease while T1_free_ increases.^96^ However, changes are subtle, and month-by-month change may be less predictable for reactivating CELs.

Increasing lesion severity coincides with decreasing *k_f_*^87,96,112,116^ and *k*_sat,_^86^ while conversely T1_free_^87,112^ and T1_sat_^86^ are elevated in acute, compared with mild, lesions. However, dense CELs have higher *k_f_* but lower T1_free_ values than ring CELs.^87^ *F*^106^, *f*^36,118^, R1_free_^106,94^ and T_2bound,_^36,94^ are also reduced in lesions compared with NAWM and control WM, with reduced *F* and R_1free_ in T2 hyperintense lesions visible on selective inversion recovery-derived parametric maps.^104,105^ Finally, MMC is reduced in CELs but may recover post-enhancement.^93^ The relationship between pathology and qMT-derived metrics is evidently complex, but may still differentiate between lesions with similar MTR, particularly when lesions are T1-w isointense.^112^

**Figure 6:**
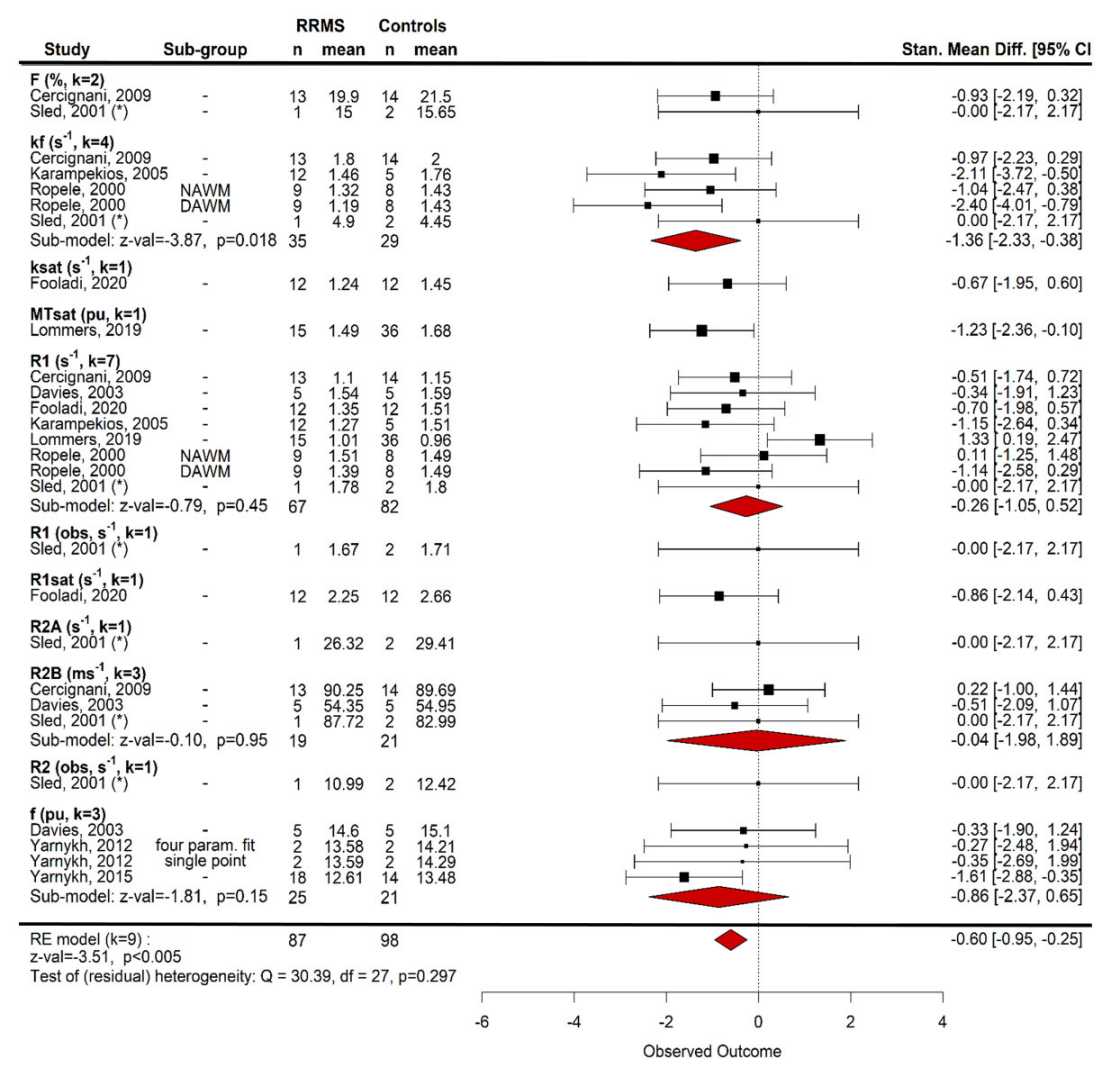
**Random-effects meta-analysis of magnetization transfer compartmental model parameters in WM**. Metric was a nested factor within study and subgroup (e.g. DAWM versus NAWM) was nested within metric. T1 and T2 were converted to R1 and R2, respectively, for comparability. For people with RRMS, compartmental model metrics were significantly lower than HCs (standardized mean difference −0.60, *z*-value = −3.51, *P* = 0.002, nine studies, 87 RRMS/98 HC). Random-effects models for individuals metrics were not significant after correction for multiple comparisons, despite a trend for the forward exchange rate, *k_f_* (standardized mean difference −1.36, *z*-value = −3.87, *P* = 0.018, four studies). R1 (−0.26, *z*-value = −0.79, *P* = 0.45, seven studies), R2B (−0.04, *z*-value = −0.10, *P* = 0.95, three studies) and *f* (−0.86, *z*-value = 1.81, *P* = 0.15, three studies) did not differ between patients and HCs. DAWM, dirty-appearing white matter; NAWM, normal-appearing white matter; Stand Mean Diff, standardized mean difference. (*) frontal white matter; *α* = 0.05 for omnibus test and *α* = 0.05/4 = 0.0125 for subgroups.

Differences between NAWM and control WM qMT are, however, subtle. Some studies report differences for qihMT,^119^ T1_free_,^112^ *F*^94^ and *k_f_*,^87,94,112^ while others show no differences for *k_f_*,^64,116^ *F*,^64^ *f*,^36^ T2_bound_,^36^ T1_free_,^87^ R1_free_^94^ or qMT.^119^ Nine studies were submitted to a random-effects meta-analysis to compare qMT in NAWM and WM.^36,86,87,94,112,116,118^ There was a significant difference between patients and controls across all qMT metrics [standardized mean difference −0.60 (95% CI −0.95 to −0.25), *z*-value: −3.51, *P* < 0.005, *n* = 87 RRMS/98 HCs, Fig. 6]. Additional follow-up models for metrics where *k* ≥ 3, however, showed no significant difference for R1_free_, R2_bound_, *f* and *k_f_* (*α* = 0.0125, Fig. 6) despite a trend for *k_f_*. Other brain regions were not assessed due to limited data.

In cortical grey matter, *k_f_*, *F*, R1_free_ and T2_bound_ appear lower and T2_free_ higher than in lesions and frontal WM.^116^ RRMS patients have lower *k_f_* than controls in cortical grey matter but *F* does not differ, except for patients with high disability.^64^ No differences between patients and controls were found in cerebral or cerebellar grey matter for *f*, T1_free_ or T2_bound_.^36^ In deep grey matter, *f* was lower for patients than controls.^118^ However, differences in methodology can results in over- or underestimation of *f* in certain ROIs (e.g. thalami).^118^

Few studies have examined the relationship between qMT and clinical disability in RRMS. Cortical grey matter *k_f_* may be negatively associated with EDSS and Choice Reaction Time, but not SDMT or PASAT.^64^ Associations between EDSS and both qMT and qihMT in lesions, but not NAWM have also been reported.^119^ Combining qMT parameters, and including covariates such as lesion load and age may improve models^94^ but collinearity (e.g. between *f* and T2_bound_ or *k_f_* and T1_free_) may be problematic if used in the same model.^36,112^

### Risk of bias

Seven studies (8.1%) were given an ‘*excellent*’ rating based on JBI Critical Appraisal Checklist criteria (Supplementary Table 10). The majority of studies rated ‘*good*’ or ‘*ok*’ (*k* = 33, 38.4% each) and 13 studies (15.1%) were given a ‘*poor*’ rating. The latter result, however, was partly driven by methodological ‘proof of principle’ studies for which there was no specific checklist.

Overall, the main sources of bias, where relevant, were inadequate examination of confounding factors, poor standardization and reliability of MTI outcomes, inappropriate statistical analyses, particularly concerning no correction for multiple comparisons, poor matching of cases and controls, and a lack of detail regarding setting/site description. Funnel plots also suggest that case–control studies with high precision are lacking, particularly for analyses of grey matter (Supplementary Fig. 4). Similarly, there appears to be a bias towards small, less powerful studies which examined the relationship between clinical disability and MTI in WM (Supplementary Fig. 5). In contrast, studies that used compartmental models had relatively high precision, particularly R1 and MTsat (Supplementary Fig. 6).

## Discussion

Our search demonstrated a broad literature of MS-specific MTI studies, a considerable number of which were excluded due to the lack of distinctions between MS subtypes or grouped subtypes in analyses and results. Eighty-six studies used MTI to investigate cerebral RRMS pathology, the vast majority (87%) of which used MTR. We also incorporated in meta-analyses additional RRMS data from a further 38 studies which included mixed MS subtypes.

### Common findings

Lesion MT was found to be lower than in NAWM. MT was also generally reduced in non-lesional brain for patients compared with HCs, indicative of subtle loss in microstructural integrity. Conversely, smaller sub-regions (e.g. thalamus, putamen) did not show such differences. The absolute sensitivity of MT metrics to pathological changes in the brain of people with MS is modest; the difference in MTR between patients with RRMS and HCs is estimated to be small (∼0.5–2%) compared with inter-study variability. Meta-analyses did not support a significant annual longitudinal decline in MT in RRMS despite qualitative evidence to the contrary and a trend in NABT. In lesions, MT is inclined to fluctuate over time.

Although associations between MT measures and clinical disability in RRMS were apparent, relationships were weak, and confounded by factors such as age. This association may be limited by the lack of longitudinal data over sufficient time periods for divergence in disability to become apparent.

Studies examining longitudinal change and clinical correlates were limited to MTR; we did not identify any such studies using other techniques, such as MTsat, ihMTR or qMT.

#### Sample characteristics

Overall, patient sample sizes across the RRMS MTI literature were small, with a median of <20 subjects, and many studies were statistically underpowered. Research with a technical or proof-of-concept focus tended to include a single subject or handful of participants (e.g.^11,42,105,106,116,118^). Conversely, international clinical trials recruited much larger cohorts (e.g.^91,92^), but at the expense of standardized, well-documented MTI protocols.

Comparisons between MS and (typically) age-matched HC subjects featured in a number of studies, albeit often with smaller control than patient groups. Such well-matched control data are important to account for confounding variables such as age,^85^ and may additionally provide reference measures to help improve comparability of MT metrics across studies and centres.

Treatment effects are a further potential confound of MT microstructure measures, and inter- and intra-study heterogeneity was apparent in DMT and steroid usage which is an additional source of variability. Although some studies control for treatment effects, greater consistency is required in studies whose primary focus is imaging biomarker validation.

#### Imaging acquisition protocols

Systematic comparison of MTI in RRMS demonstrates substantial heterogeneity of MTI acquisition protocols. There was wide variation in magnetic field strength, pulse sequence, image weighting, excitation flip angle, TR and TE. With the rapid evolution of MRI hardware and techniques, such sources of variation are inevitable and well-recognized in the quantitative MRI literature. The nature of MT acquisition, however, makes MT measurements particularly sensitive to these factors. For example, simulations suggest that the difference between grey and WM MTR at 3 T at an offset frequency of 1.5 kHz is around 43% larger than at 1.5 T.^117^ Use of proprietary hardware and pulse sequences allows broader access of MTI to research groups with limited MRI pulse programming expertise, but typically fixes, restricts and even conceals important pulse sequence parameters.

MT measurements are especially sensitive to characteristics of the MT pulse. Quantification typically assumes selective saturation of the ‘bound’ pool with minimal direct saturation of the ‘free’ water pool. The extent to which this is achieved *in vivo* and the resulting tissue-type contrast, however, depends on the complex relationship between tissue properties, hardware, sequence parameters and MT pulse design features including the offset frequency, power, pulse duration and shape.^98^ In particular, our finding of the wide variance in NAWM MTR in RRMS cohorts is suggestive of sequence parameter dependence. Early experiments with relatively low offsets (e.g.^110,113^) are likely to have a greater direct saturation effect. Improved harmonization and standardization of MT protocols between centres would help to minimize these sources of variability.

The majority of large-scale MT studies in RRMS to date have used MTR, which is relatively easy to acquire and analyse. Importantly, however, MTR signal is markedly dependent on T1 and B1 effects in addition to magnetization transfer processes, which limits its specificity as a microstructural imaging marker of myelin integrity.

qMT provides the most accurate modelling of MT processes and is helpful for probing microstructure in healthy and pathological tissue; however, prolonged acquisition is needed at multiple pulse powers and offset frequencies with adequate spatial resolution. Whole-brain coverage is therefore not currently feasible for clinical imaging in patients.

Emerging MT methods such as MTsat and ihMTR provide potentially more robust and specific measures of myelin integrity than MTR within clinically feasible acquisition times.^11,121^ Histological validation in felines has shown that MTsat is sensitive to demyelination,^122^ and, in mice, ihMTR signal is more specific to myelin than MTR.^121^ Both techniques, however, require further validation with histology and study in larger patient and HC cohorts.

#### Tissue types and definitions

The substantial variation observed in MTR values for different tissue types is likely due not only to varying acquisition parameters discussed above, but also how tissue type is defined, and variations in methods by which the regions are segmented from structural imaging. For example, individual studies examine different combinations of WM, NAWM, cortical and deep grey matter structures, atlas-based ROIs, and whole-brain analyses. Moreover, a number of different ‘lesion types’ are recognized in RRMS, as defined by their signal characteristics; for example, T2-w or FLAIR hyperintensities, T1-w hypointense lesions or ‘black holes’, and contrast-enhancing lesions. A clear definition of lesion subtypes is therefore important for the interpretation of their MT characteristics.

### Sources of bias and limitations

Study quality, including assessment ratings of application of methods to minimize bias, was variable; the large majority of studies classified as ‘good’ or ‘ok’, and those rated ‘poor’ were largely associated with small methodologically focused papers.

Bias was apparent towards small sample sizes, and also towards studies using MTR compared with other techniques. Overall, high precision case–control studies were lacking and bias was apparent towards small, less well-powered studies correlating clinical disability with MTI measures. Overall, the small number of studies that used compartmental MTI models showed relatively high precision compared with MTR. Inadequate examination of confounding factors, poor standardization and reliability of acquisition methods, flawed statistical analyses, poor matching of cases and controls and lack of detail regarding the research setting were also identified in a significant number of studies.

Across studies, there was a near-universal bias towards European and North American populations, which is likely to reflect the geographical prevalence of MS, the attention given to the disease within healthcare systems, and access to MRI and research protocols. Importantly, analysis of the location of study centres highlights possible bias due to data duplication from multiple or overlapping analyses of cohorts. This is rarely overtly reported, but may influence the calculation of effect sizes.

With regard to the review process, the literature search procedure was carried out by a single reviewer which may have led to bias in study selection, and influence overall certainty of evidence. Meta-analyses were limited by large inter-study protocol heterogeneity and missing data, and also did not take into account patient or control group demographics. The scope of the present review is also limited to results in RRMS patients. Data from progressive MS subtypes were excluded, but may still provide insights on how MT metrics reflect microstructural damage in MS.

### Implications for future studies using MT in RRMS

The findings of this review indicate the potential for MT measures of microstructure as useful disease markers in MS, but equally highlight large variability in quantitative findings compared with modest effect sizes.

Major sources of systematic differences and variance in MTR measured across studies are technical variation in acquisition protocols, and confounding magnetic field homogeneity (B1) and magnetization relaxation processes (notably T1); relaxation processes, in particular, may lead to bidirectional longitudinal fluctuations in MTR. These effects, combined with variability in cohort characteristics and experimental design, contribute to weak association with clinical measures of disease.

Harmonizing MTR acquisition protocols across participating centres will go some way to mitigate this variability, although will not address the confounds of B1 and T1 effects. Signal from more quantitative, clinically applicable MT methods such as MTsat and ihMT is less confounded by these technical features and other tissue characteristics, and hence provide more specific biomarkers of myelin status. These methods, however, require further evaluation, with rigorous validation against tissue reference data, and other biomarkers of MS disease activity and neurodegeneration.

Cohorts which are adequately powered to detect predicted effect sizes are likely to require large multicentre studies of highly characterized patients with defined MS disease subtypes. Further optimization, harmonization and cross-site validation of MTI protocols across multiple MRI platforms, will allow assessment of inter-site variance and potential systematic differences in measures across centres.

Adoption of more consistent definitions and methods for segmenting tissues of interest will also facilitate comparability across sites and studies.

We, therefore, expect that moving towards more quantifiable, harmonized MT protocols in large well-defined and annotated cohorts will provide a more reliable indication of the relationships between MT and clinical features in MS, and hence their potential utility in patient stratification and clinical trial platforms.

Moreover, we suggest that in order for MTI to evolve as a useful imaging tool in MS and other diseases, there is a need to establish consensus standards for image acquisition, analysis and reporting from an international group of experts working across centres, as has been successfully achieved with other quantitative MRI methods such as diffusion and perfusion imaging.^123–125^

## Conclusion

This systematic review demonstrates a substantial literature on MTR applied to RRMS. The evidence evaluated suggests that MT imaging can detect subtle disease-related differences. There is, however, large measurement variability due to differences in technique; this dominates over small effect sizes which, in turn, limit clinical and biological interpretation. The implementation of more robust emerging quantitative techniques, and consensus regarding optimized, harmonized protocols in large well-characterized patient cohorts will be required to establish the value of MTI as a useful microstructural marker in RRMS, for translation into wider clinical use.

## Supplementary Material

fcac088_Supplementary_Data

## Abbreviations

9HPT =: nine-hole peg test
APLA =: anti-phospholipid antibody
BDNF =: brain-derived neurotrophic factor
CELs =: contrast-enhancing lesions
CI =: confidence interval
DMTs =: disease-modifying therapies
EDSS =: Expanded Disability Status Scale
FLAIR =: fluid-attenuated inversion recovery
IfN-α/β =: interferon-alpha/beta
ihMTR =: inhomogeneous magnetization transfer ratio
MS =: multiple sclerosis
MTI =: magnetization transfer imaging
MSFC =: multiple sclerosis functional composite
MTR =: magnetization transfer ratio
MTsat =: magnetization transfer saturation
NABT =: normal-appearing brain tissue
NAGM =: normal-appearing grey matter
NAWM =: normal-appearing white matter
PASAT =: Paced Auditory Serial Addition Test
PPMS =: primary progressive multiple sclerosis
qMT =: quantitative magnetization transfer
RNFL =: retinal nerve fibre layer
ROI =: region of interest
RRMS =: relapsing-remitting multiple sclerosis
SDMT =: Symbol-Digit Modalities Test
SE =: spin echo
SPMS =: secondary progressive multiple sclerosis
T1-w =: T_1_-weighted
T2-w =: T_2_-weighted
T25FW =: Timed 25-Foot Walk
TE =: echo time
TR =: repetition time
WM =: white matter

## References

[1] National Multiple Sclerosis Society. Accessed 21 June 2019. https://www.nationalmssociety.org/What-is-MS/Definition-of-MS.

[2] Harris VK, Tuddenham JF, Sadiq SA. Biomarkers of multiple sclerosis: Current findings. Degener Neurol Neuromuscul Dis. 2017;7:19–29. 30050375 10.2147/DNND.S98936 PMC6053099

[3] Barkhof F. MRI in multiple sclerosis: Correlation with expanded disability status scale (EDSS). Mult Scler. 1999;5(4):283–286. 10467389 10.1177/135245859900500415

[4] Li DK, Held U, Petkau J, et al MRI T2 lesion burden in multiple sclerosis: A plateauing relationship with clinical disability. Neurology. 2006;66(9):1384–1389. 16682671 10.1212/01.wnl.0000210506.00078.5c

[5] Agosta F, Rovaris M, Pagani E, Sormani MP, Comi G, Filippi M. Magnetization transfer MRI metrics predict the accumulation of disability 8 years later in patients with multiple sclerosis. Brain. 2006;129(Pt 10):2620–2627. 16951409 10.1093/brain/awl208

[6] Sled JG. Modelling and interpretation of magnetization transfer imaging in the brain. Neuroimage. 2018;182:128–135. 29208570 10.1016/j.neuroimage.2017.11.065

[7] Wolff SD, Balaban RS. Magnetization transfer contrast (MTC) and tissue water proton relaxation in vivo. Magn Reson Med. 1989;10(1):135–144. 2547135 10.1002/mrm.1910100113

[8] Henkelman RM, Huang X, Xiang Q-S, Stanisz GJ, Swanson SD, Bronskill MJ. Quantitative interpretation of magnetization transfer. Magn Reson Med. 1993;29(6):759–766. 8350718 10.1002/mrm.1910290607

[9] Horsfield MA. Magnetization transfer imaging in multiple sclerosis. J Neuroimaging. 2005;15(4 Suppl):58S–67S. 16385019 10.1177/1051228405282242

[10] Samson RS, Wheeler-Kingshott CAM, Symms MR, Tozer DJ, Tofts PS. A simple correction for B1 field errors in magnetization transfer ratio measurements. Magn Reson Imaging. 2006;24(3):255–263. 16563954 10.1016/j.mri.2005.10.025

[11] Helms G, Dathe H, Kallenberg K, Dechent P. High-resolution maps of magnetization transfer with inherent correction for RF inhomogeneity and *T*_1_ relaxation obtained from 3D FLASH MRI. Magn Reson Med. 2008;60(6):1396–1407. 19025906 10.1002/mrm.21732

[12] Helms G, Dathe H, Dechent P. Quantitative FLASH MRI at 3 T using a rational approximation of the Ernst equation. Magn Reson Med. 2008;59(3):667–672. 18306368 10.1002/mrm.21542

[13] Manning AP, Chang KL, MacKay AL, Michal CA. The physical mechanism of “inhomogeneous” magnetization transfer MRI. J Magn Reson. 2017;274:125–136. 27918896 10.1016/j.jmr.2016.11.013

[14] Varma G, Girard OM, Prevost VH, Grant AK, Duhamel G, Alsop DC. Interpretation of magnetization transfer from inhomogeneously broadened lines (ihMT) in tissues as a dipolar order effect within motion restricted molecules. J Magn Reson. 2015;260:67–76. 26408956 10.1016/j.jmr.2015.08.024

[15] Varma G, Duhamel G, de Bazelaire C, Alsop DC. Magnetization transfer from inhomogeneously broadened lines: A potential marker for myelin. Magn Reson Med. 2015;73(2):614–622. 24604578 10.1002/mrm.25174 PMC4378005

[16] Ramani A, Dalton C, Miller DH, Tofts PS, Barker GJ. Precise estimate of fundamental in-vivo MT parameters in human brain in clinically feasible times. Magn Reson Imaging. 2002;20(10):721–731. 12591568 10.1016/s0730-725x(02)00598-2

[17] Henkelman RM, Stanisz GJ, Graham SJ. Magnetization transfer in MRI: A review. NMR Biomed. 2001;14(2):57–64. 11320533 10.1002/nbm.683

[18] Filippi M, Agosta F. Magnetization transfer MRI in multiple sclerosis. J Neuroimaging. 2007;17(Suppl 1):22S–26S. 17425730 10.1111/j.1552-6569.2007.00132.x

[19] Pike GB. Magnetization transfer imaging of multiple sclerosis. Ital J Neurol Sci. 1997;18(6):359–365. 9494868 10.1007/BF02048239

[20] Weiskopf N, Edwards LJ, Helms G, Mohammadi S, Kirilina E. Quantitative magnetic resonance imaging of brain anatomy and in vivo histology. Nat Rev Phys. 2021;3:570–588.

[21] Lazari A, Lipp I. Can MRI measure myelin? Systematic review, qualitative assessment, and meta-analysis of studies validating microstructural imaging with myelin histology. NeuroImage. 2021;230:117744. 33524576 10.1016/j.neuroimage.2021.117744 PMC8063174

[22] van der Weijden CWJ, Garcia DV, Borra RJH, et al Myelin quantification with MRI: A systematic review of accuracy and reproducibility. Neuroimage. 2021;226:117561. 33189927 10.1016/j.neuroimage.2020.117561

[23] Campbell JSW, Leppert IR, Narayanan S, et al Promise and pitfalls of g-ratio estimation with MRI. Neuroimage. 2018;182:80–96. 28822750 10.1016/j.neuroimage.2017.08.038

[24] Mohammadi S, Callaghan MF. Towards in vivo g-ratio mapping using MRI: Unifying myelin and diffusion imaging. J Neurosci Methods. 2021;348:108990. 33129894 10.1016/j.jneumeth.2020.108990 PMC7840525

[25] Cooper G, Hirsch S, Scheel M, et al Quantitative multi-parameter mapping optimized for the clinical routine. Front Neurosci. 2020;14:611194. 33364921 10.3389/fnins.2020.611194 PMC7750476

[26] Weinshenker BG, Bass B, Rice GP, et al The natural history of multiple sclerosis: A geographically based study. I. Clinical course and disability. Brain. 1989;112(Pt 1):133–146. 2917275 10.1093/brain/112.1.133

[27] Bross M, Hackett M, Bernitsas E. Approved and emerging disease modifying therapies on neurodegeneration in multiple sclerosis. Int J Mol Sci. 2020;21(12):4312. 10.3390/ijms21124312 PMC7348940 32560364

[28] Hauser SL, Cree BAC. Treatment of multiple sclerosis: A review. Am J Med. 2020;133(12):1380–1390.e2. 32682869 10.1016/j.amjmed.2020.05.049 PMC7704606

[29] McGinley MP, Goldschmidt CH, Rae-Grant AD. Diagnosis and treatment of multiple sclerosis: A review. JAMA. 2021;325(8):765–779. 33620411 10.1001/jama.2020.26858

[30] Martin S-J, McGlasson S, Hunt D, Overell J. Cerebrospinal fluid neurofilament light chain in multiple sclerosis and its subtypes: A meta-analysis of case–control studies. J Neurol Neurosurg Psychiatry. 2019;90(9):1059–1067. 31123141 10.1136/jnnp-2018-319190 PMC6820150

[31] Moher D, Liberati A, Tetzlaff J, Altman DG, PRISMA Group. Preferred reporting items for systematic reviews and meta-analyses: The PRISMA statement. PLoS Med. 2009;6(7):e1000097. 19621072 10.1371/journal.pmed.1000097 PMC2707599

[32] Page MJ, McKenzie JE, Bossuyt PM, et al The PRISMA 2020 statement: An updated guideline for reporting systematic reviews. BMJ. 2021;372:n71. 33782057 10.1136/bmj.n71 PMC8005924

[33] Moola S, Munn Z, Tufanaru C, et al Systematic reviews of etiology and risk. In: Aromataris E, Munn Z, eds. JBI manual for evidence synthesis. JBI; 2020.

[34] Tufanaru C, Munn Z, Aromataris E, Campbell J, Hopp L. Systematic reviews of effectiveness. In: Aromataris E, Munn Z, eds. JBI manual for evidence synthesis. JBI; 2020.

[35] Colasanti A, Guo Q, Muhlert N, et al In vivo assessment of brain white matter inflammation in multiple sclerosis with _18_F-PBR111 PET. J Nucl Med. 2014;55(7):1112–1118. 24904112 10.2967/jnumed.113.135129

[36] Davies GR, Ramani A, Dalton CM, et al Preliminary magnetic resonance study of the macromolecular proton fraction in white matter: A potential marker of myelin? Multi Scler. 2003;9(3):246–249. 10.1191/1352458503ms911oa 12814170

[37] Davies GR, Ramió-Torrentà L, Hadjiprocopis A, et al Evidence for grey matter MTR abnormality in minimally disabled patients with early relapsing-remitting multiple sclerosis. J Neurol Neurosurg Psychiatry. 2004;75(7):998–1002. 15201359 10.1136/jnnp.2003.021915 PMC1739100

[38] Davies GR, Altmann DR, Hadjiprocopis A, et al Increasing normal–appearing grey and white matter magnetisation transfer ratio abnormality in early relapsing-remitting multiple sclerosis. J Neurol. 2005;252(9):1037–1044. 15834645 10.1007/s00415-005-0808-x

[39] Davies GR, Altmann DR, Rashid W, et al Emergence of thalamic magnetization transfer ratio abnormality in early relapsing-remitting multiple sclerosis. Mult Scler. 2005;11(3):276–281. 15957507 10.1191/1352458505ms1166oa

[40] Griffin CM, Chard DT, Parker GJM, Barker GJ, Thompson AJ, Miller DH. The relationship between lesion and normal appearing brain tissue abnormalities in early relapsing remitting multiple sclerosis. J Neurol. 2002;249(2):193–199. 11985386 10.1007/pl00007864

[41] Muhlert N, Atzori M, De Vita E, et al Memory in multiple sclerosis is linked to glutamate concentration in grey matter regions. J Neurol Neurosurg Psychiatry. 2014;85(8):833–839. 24431465 10.1136/jnnp-2013-306662 PMC4112488

[42] Yiannakas MC, Tozer DJ, Schmierer K, et al ADvanced IMage Algebra (ADIMA): A novel method for depicting multiple sclerosis lesion heterogeneity, as demonstrated by quantitative MRI. Mult Scler. 2013;19(6):732–741. 23037551 10.1177/1352458512462074 PMC4103823

[43] Cercignani M, Iannucci G, Rocca MA, Comi G, Horsfield MA, Filippi M. Pathologic damage in MS assessed by diffusion-weighted and magnetization transfer MRI. Neurology. 2000;54:1139–1144. 10720288 10.1212/wnl.54.5.1139

[44] Codella M, Rocca MA, Colombo B, Martinelli-Boneschi F, Comi G, Filippi M. Cerebral grey matter pathology and fatigue in patients with multiple sclerosis: A preliminary study. J Neurol Sci. 2002;194(1):71–74. 11809169 10.1016/s0022-510x(01)00682-7

[45] Filippi M, Rocca MA, Rizzo G, et al Magnetization transfer ratios in multiple sclerosis lesions enhancing after different doses of gadolinium. Neurology. 1998;50(5):1289–1293. 9595976 10.1212/wnl.50.5.1289

[46] Filippi M, Rocca MA, Sormani MP, Pereira C, Comi G. Short-term evolution of individual enhancing MS lesions studied with magnetization transfer imaging. Magn Reson Imaging. 1999;17(7):979–984. 10463647 10.1016/s0730-725x(99)00049-1

[47] Iannucci G, Rovaris M, Giacomotti L, Comi G, Filippi M. Correlation of multiple sclerosis measures derived from T2-weighted, T1-weighted, magnetization transfer, and diffusion tensor MR imaging. Am J Neuroradiol. 2001;22(8):1462–1467. 11559491 PMC7974567

[48] Oreja-Guevara C, Charil A, Caputo D, Cavarretta R, Sormani MP, Filippi M. Magnetization transfer magnetic resonance imaging and clinical changes in patients with relapsing-remitting multiple sclerosis. Arch Neurol. 2006;63(5):736–740. 16682543 10.1001/archneur.63.5.736

[49] Preziosa P, Pagani E, Moiola L, Rodegher M, Filippi M, Rocca MA. Occurrence and microstructural features of slowly expanding lesions on fingolimod or natalizumab treatment in multiple sclerosis. Mult Scler. 2021;27:1520–1532. 33183125 10.1177/1352458520969105

[50] Rocca MA, Falini A, Colombo B, Scotti G, Comi G, Filippi M. Adaptive functional changes in the cerebral cortex of patients with nondisabling multiple sclerosis correlate with the extent of brain structural damage. Ann Neurol. 2002;51(3):330–339. 11891828 10.1002/ana.10120

[51] Bonnier G, Roche A, Romascano D, et al Advanced MRI unravels the nature of tissue alterations in early multiple sclerosis. Ann Clin Transl Neurol. 2014;1(6):423–432. 25356412 10.1002/acn3.68 PMC4184670

[52] Bonnier G, Roche A, Romascano D, et al Multicontrast MRI quantification of focal inflammation and degeneration in multiple sclerosis. Biomed Res Int. 2015;2015:569123. 26295042 10.1155/2015/569123 PMC4532805

[53] Bonnier G, Maréchal B, Fartaria MJ, et al The combined quantification and interpretation of multiple quantitative magnetic resonance imaging metrics enlightens longitudinal changes compatible with brain repair in relapsing-remitting multiple sclerosis patients. Front Neurol. 2017;8:506. 29021778 10.3389/fneur.2017.00506 PMC5623825

[54] Bonnier G, Fischi-Gomez E, Roche A, et al Personalized pathology maps to quantify diffuse and focal brain damage. NeuroImage Clin. 2019;21:101607. 30502080 10.1016/j.nicl.2018.11.017 PMC6413479

[55] Kuhle J, Barro C, Disanto G, et al Serum neurofilament light chain in early relapsing remitting MS is increased and correlates with CSF levels and with MRI measures of disease severity. Mult Scler. 2016;22(12):1550–1559. 26754800 10.1177/1352458515623365

[56] Romascano D, Meskaldji D-E, Bonnier G, et al Multicontrast connectometry: A new tool to assess cerebellum alterations in early relapsing-remitting multiple sclerosis. Hum Brain Mapp. 2015;36(4):1609–1619. 25421928 10.1002/hbm.22698 PMC6869568

[57] Audoin B, Davies G, Rashid W, Fisniku L, Thompson AJ, Miller DH. Voxel-based analysis of grey matter magnetization transfer ratio maps in early relapsing remitting multiple sclerosis. Mult Scler. 2007;13(4):483–489. 17463071 10.1177/1352458506070450

[58] Bellmann-Strobl J, Stiepani H, Wuerfel J, et al MR spectroscopy (MRS) and magnetisation transfer imaging (MTI), lesion load and clinical scores in early relapsing remitting multiple sclerosis: A combined cross-sectional and longitudinal study. Eur Radiol. 2009;19(8):2066–2074. 19308417 10.1007/s00330-009-1364-z

[59] Deloire MSA, Ruet A, Hamel D, Bonnet M, Dousset V, Brochet B. MRI predictors of cognitive outcome in early multiple sclerosis. Neurology. 2011;76(13):1161–1167. 21444901 10.1212/WNL.0b013e318212a8be PMC3312081

[60] Mangia S, Carpenter AF, Tyan AE, Eberly LE, Garwood M, Michaeli S. Magnetization transfer and adiabatic T1ρ MRI reveal abnormalities in normal-appearing white matter of subjects with multiple sclerosis. Mult Scler. 2014;20(8):1066–1073. 24336350 10.1177/1352458513515084 PMC4205209

[61] Rudick RA, Lee JC, Simon J, Fisher E. Significance of T2 lesions in multiple sclerosis: A 13-year longitudinal study. Ann Neurol. 2006;60(2):236–242. 16786526 10.1002/ana.20883

[62] Fritz NE, Keller J, Calabresi PA, Zackowski KM. Quantitative measures of walking and strength provide insight into brain corticospinal tract pathology in multiple sclerosis. Neuroimage Clin. 2017;14:490–498. 28289599 10.1016/j.nicl.2017.02.006 PMC5338912

[63] Lin X, Tench CR, Morgan PS, Constantinescu CS. Use of combined conventional and quantitative MRI to quantify pathology related to cognitive impairment in multiple sclerosis. J Neurol Neurosurg Psychiatry. 2008;79(4):437–441. 17673493 10.1136/jnnp.2006.112177

[64] McKeithan LJ, Lyttle BD, Box BA, et al 7 T quantitative magnetization transfer (qMT) of cortical gray matter in multiple sclerosis correlates with cognitive impairment. Neuroimage. 2019;203:116190. 31525497 10.1016/j.neuroimage.2019.116190 PMC7549401

[65] Zivadinov R, De Masi R, Nasuelli D, et al MRI techniques and cognitive impairment in the early phase of relapsing-remitting multiple sclerosis. Neuroradiology. 2001;43(4):272–278. 11338408 10.1007/s002340000500

[66] Bernitsas E, Kopinsky H, Lichtman-Mikol S, et al Multimodal MRI response to fingolimod in multiple sclerosis: A nonrandomized, single arm, observational study. J Neuroimaging. 2021;31:379–387. 33368776 10.1111/jon.12824

[67] Thaler C, Faizy TD, Sedlacik J, et al The use of multiparametric quantitative magnetic resonance imaging for evaluating visually assigned lesion groups in patients with multiple sclerosis. J Neurol. 2018;265(1):127–133. 10.1007/s00415-017-8683-9 29159467

[68] Schwartz DL, Tagge I, Powers K, et al Multisite reliability and repeatability of an advanced brain MRI protocol. J Magn Reson Imaging. 2019;50(3):878–888. 30652391 10.1002/jmri.26652 PMC6636359

[69] Zivadinov R, Dwyer MG, Markovic-Plese S, et al Effect of treatment with interferon beta-1a on changes in voxel-wise magnetization transfer ratio in normal appearing brain tissue and lesions of patients with relapsing–remitting multiple sclerosis: A 24-week, controlled pilot study. PLoS One. 2014;9(3):e91098. 24625687 10.1371/journal.pone.0091098 PMC3953325

[70] Richert ND, Ostuni JL, Bash CN, Duyn JH, McFarland HF, Frank JA. Serial whole-brain magnetization transfer imaging in patients with relapsing-remitting multiple sclerosis at baseline and during treatment with interferon beta-1b. Am J Neuroradiol. 1998;19(9):1705–1713. 9802494 PMC8337498

[71] Richert ND, Ostuni JL, Bash CN, Leist TP, McFarland HF, Frank JA. Interferon beta-1b and intravenous methylprednisolone promote lesion recovery in multiple sclerosis. Mult Scler. 2001;7(1):49–58. 11321194 10.1177/135245850100700109

[72] Ernst T, Chang L, Walot I, Huff K. Physiologic MRI of a tumefactive multiple sclerosis lesion. Neurology. 1998;51(5):1486–1488. 9818892 10.1212/wnl.51.5.1486

[73] Kita M, Goodkin DE, Bacchetti P, Waubant E, Nelson SJ, Majumdar S. Magnetization transfer ratio in new MS lesions before and during therapy with IFNbeta-1a. Neurology. 2000;54(9):1741–1745. 10802778 10.1212/wnl.54.9.1741

[74] Zivadinov R, Ramanathan M, Ambrus J, et al Anti-phospholipid antibodies are associated with response to interferon-beta1a treatment in MS: Results from a 3-year longitudinal study. Neurol Res. 2012;34(8):761–769. 22971466 10.1179/1743132812Y.0000000076

[75] Zivadinov R, Hussein S, Stosic M, et al Glatiramer acetate recovers microscopic tissue damage in patients with multiple sclerosis. A case–control diffusion imaging study. Pathophysiology. 2011;18:61–68. 20510590 10.1016/j.pathophys.2010.04.007

[76] Patel UJ, Grossman RI, Phillips MD, et al Serial analysis of magnetization-transfer histograms and Expanded Disability Status Scale scores in patients with relapsing-remitting multiple sclerosis. Am J Neuroradiol. 1999;20(10):1946–1950. 10588123 PMC7657806

[77] Levesque IR, Giacomini PS, Narayanan S, et al Quantitative magnetization transfer and myelin water imaging of the evolution of acute multiple sclerosis lesions. Magn Reson Med. 2010;63(3):633–640. 20146232 10.1002/mrm.22244

[78] Catalaa I, Grossman RI, Kolson DL, et al Multiple sclerosis: Magnetization transfer histogram analysis of segmented normal-appearing white matter. Radiology. 2000;216(2):351–355. 10924552 10.1148/radiology.216.2.r00au16351

[79] Ge Y, Grossman RI, Babb JS, He J, Mannon LJ. Dirty-appearing white matter in multiple sclerosis: Volumetric MR imaging and magnetization transfer ratio histogram analysis. Am J Neuroradiol. 2003;24(10):1935–1940. 14625213 PMC8148922

[80] Mesaros S, Rocca M, Sormani M, et al Bimonthly assessment of magnetization transfer magnetic resonance imaging parameters in multiple sclerosis: A 14-month, multicentre, follow-up study. Mult Scler. 2010;16(3):325–331. 20086023 10.1177/1352458509358713

[81] De Stefano N, Narayanan S, Francis SJ, et al Diffuse axonal and tissue injury in patients with multiple sclerosis with low cerebral lesion load and no disability. Arch Neurol. 2002;59(10):1565. 12374493 10.1001/archneur.59.10.1565

[82] Frohman EM, Dwyer MG, Frohman T, et al Relationship of optic nerve and brain conventional and non-conventional MRI measures and retinal nerve fiber layer thickness, as assessed by OCT and GDx: A pilot study. J Neurol Sci. 2009;282(1–2):96–105. 19439327 10.1016/j.jns.2009.04.010

[83] Goodkin DE, Rooney WD, Sloan R, et al A serial study of new MS lesions and the white matter from which they arise. Neurology. 1998;51(6):1689–1697. 9855524 10.1212/wnl.51.6.1689

[84] Rovira A, Alonso J, Cucurella G, et al Evolution of multiple sclerosis lesions on serial contrast-enhanced T1-weighted and magnetization-transfer MR images. Am J Neuroradiol. 1999;20(10):1939–1945. 10588122 PMC7657782

[85] Amann M, Sprenger T, Naegelin Y, et al Comparison between balanced steady-state free precession and standard spoiled gradient echo magnetization transfer ratio imaging in multiple sclerosis: Methodical and clinical considerations. Neuroimage. 2015;108:87–94. 25536494 10.1016/j.neuroimage.2014.12.045

[86] Fooladi M, Riyahi Alam N, Sharini H, Firouznia K, Shakiba M, Harirchian MH. Multiparametric qMTI assessment and monitoring of normal appearing white matter and classified T1 hypointense lesions in relapsing-remitting multiple sclerosis. IRBM. 2020;30:151–160.

[87] Ropele S, Strasser-Fuchs S, Augustin M, et al A comparison of magnetization transfer ratio, magnetization transfer rate, and the native relaxation time of water protons related to relapsing-remitting multiple sclerosis. Am J Neuroradiol. 2000;21(10):1885–1891. 11110542 PMC7974275

[88] Van Obberghen E, McHinda S, le Troter A, et al Evaluation of the sensitivity of inhomogeneous magnetization transfer (ihMT) MRI for multiple sclerosis. Am J Neuroradiol. 2018;39(4):634–641. 29472299 10.3174/ajnr.A5563 PMC7410781

[89] Gracien RM, Jurcoane A, Wagner M, et al Multimodal quantitative MRI assessment of cortical damage in relapsing-remitting multiple sclerosis. J Magn Reson Imaging. 2016;44(6):1600–1607. 27153293 10.1002/jmri.25297

[90] Reitz SC, Hof S-M, Fleischer V, et al Multi-parametric quantitative MRI of normal appearing white matter in multiple sclerosis, and the effect of disease activity on T2. Brain Imaging Behav. 2017;11(3):744–753. 27138529 10.1007/s11682-016-9550-5

[91] Arnold DL, Gold R, Kappos L, et al Magnetization transfer ratio in the delayed-release dimethyl fumarate DEFINE study. J Neurol. 2014;261(12):2429–2437. 25270680 10.1007/s00415-014-7504-7 PMC4242981

[92] Miller DH, Fox RJ, Phillips JT, et al Effects of delayed-release dimethyl fumarate on MRI measures in the phase 3 CONFIRM study. Neurology. 2015;84(11):1145–1152. 25681448 10.1212/WNL.0000000000001360 PMC4371413

[93] Giacomini PS, Levesque IR, Ribeiro L, et al Measuring demyelination and remyelination in acute multiple sclerosis lesion voxels. Arch Neurol. 2009;66(3):375–381. 19273757 10.1001/archneurol.2008.578

[94] Cercignani M, Basile B, Spanò B, et al Investigation of quantitative magnetisation transfer parameters of lesions and normal appearing white matter in multiple sclerosis. NMR Biomed. 2009;22(6):646–653. 19322806 10.1002/nbm.1379

[95] Reich DS, White R, Cortese ICM, et al Sample-size calculations for short-term proof-of-concept studies of tissue protection and repair in multiple sclerosis lesions via conventional clinical imaging. Mult Scler. 2015;21(13):1693–1704. 25662351 10.1177/1352458515569098 PMC4527958

[96] Fazekas F, Ropele S, Enzinger C, Seifert T, Strasser-Fuchs S. Quantitative magnetization transfer imaging of pre-lesional white-matter changes in multiple sclerosis. Mult Scler. 2002;8(6):479–484. 12474987 10.1191/1352458502ms860oa

[97] O’Muircheartaigh J, Vavasour I, Ljungberg E, et al Quantitative neuroimaging measures of myelin in the healthy brain and in multiple sclerosis. Hum Brain Mapp. 2019;40(7):2104–2116. 30648315 10.1002/hbm.24510 PMC6590140

[98] van den Elskamp IJ, Knol DL, Vrenken H, et al Lesional magnetization transfer ratio: A feasible outcome for remyelinating treatment trials in multiple sclerosis. Mult Scler. 2010;16(6):660–669. 20350960 10.1177/1352458510364630

[99] Arnold DL, Calabresi PA, Kieseier BC, et al Peginterferon beta-1a improves MRI measures and increases the proportion of patients with no evidence of disease activity in relapsing-remitting multiple sclerosis: 2-year results from the ADVANCE randomized controlled trial. BMC Neurol. 2017;17(1):29. 28183276 10.1186/s12883-017-0799-0 PMC5301356

[100] Filippi M, Rocca MA, Pagani E, et al Placebo-controlled trial of oral laquinimod in multiple sclerosis: MRI evidence of an effect on brain tissue damage. J Neurol Neurosurg Psychiatry. 2014;85(8):851–858. 24029546 10.1136/jnnp-2013-306132

[101] Al-Radaideh A, Athamneh I, Alabadi H, Hbahbih M. Deep gray matter changes in relapsing-remitting multiple sclerosis detected by multi-parametric, high-resolution magnetic resonance imaging (MRI). Eur Radiol. 2021;31:706–715. 32851443 10.1007/s00330-020-07199-5

[102] Weinstock-Guttman B, Zivadinov R, Tamaño-Blanco M, et al Immune cell BDNF secretion is associated with white matter volume in multiple sclerosis. J Neuroimmunol. 2007;188(1–2):167–174. 17602759 10.1016/j.jneuroim.2007.06.003

[103] Zhou LQ, Zhu YM, Grimaud J, Hermier M, Rovaris M, Filippi M. A new method for analyzing histograms of brain magnetization transfer ratios: Comparison with existing techniques. Am J Neuroradiol. 2004;25(7):1234–1241. 15313716 PMC7976536

[104] Cronin MJ, Xu J, Bagnato F, Gochberg DF, Gore JC, Dortch RD. Rapid whole-brain quantitative magnetization transfer imaging using 3D selective inversion recovery sequences. Magn Reson Imaging. 2020;68:66–74. 32004710 10.1016/j.mri.2020.01.014 PMC8609909

[105] Dortch RD, Li K, Gochberg DF, et al Quantitative magnetization transfer imaging in human brain at 3 T via selective inversion recovery. Magn Reson Med. 2011;66(5):1346–1352. 21608030 10.1002/mrm.22928 PMC3285505

[106] Dortch RD, Bagnato F, Gochberg DF, Gore JC, Smith SA. Optimization of selective inversion recovery magnetization transfer imaging for macromolecular content mapping in the human brain. Magn Reson Med. 2018;80:1824–1835. 29573356 10.1002/mrm.27174 PMC6107392

[107] Fatemidokht A, Harirchian MH, Faghihzadeh E, Tafakhori A, Oghabian MA. Assessment of the characteristics of different kinds of MS lesions using multi-parametric MRI. Arch Neurosci. 2020;7(4):e102911.

[108] Fooladi M, Sharini H, Masjoodi S, Khodamoradi E. A novel classification method using effective neural network and quantitative magnetization transfer imaging of brain white matter in relapsing remitting multiple sclerosis. J Biomed Phys Eng. 2018;8(4):409–422. 30568931 PMC6280112

[109] Ge Y, Grossman RI, Udupa JK, Babb JS, Kolson DL, McGowan JC. Magnetization transfer ratio histogram analysis of gray matter in relapsing-remitting multiple sclerosis. Am J Neuroradiol. 2001;22(3):470–475. 11237968 PMC7976833

[110] Guo AC, Jewells VL, Provenzale JM. Analysis of normal-appearing white matter in multiple sclerosis: Comparison of diffusion tensor MR imaging and magnetization transfer imaging. Am J Neuroradiol. 2001;22(10):1893–1900. 11733323 PMC7973836

[111] Kamagata K, Zalesky A, Yokoyama K, et al MR g-ratio-weighted connectome analysis in patients with multiple sclerosis. Sci Rep. 2019;9:13. 31534143 10.1038/s41598-019-50025-2 PMC6751178

[112] Karampekios S, Papanikolaou N, Papadaki E, et al Quantification of magnetization transfer rate and native T1 relaxation time of the brain: Correlation with magnetization transfer ratio measurements in patients with multiple sclerosis. Neuroradiology. 2005;47(3):189–196. 15711987 10.1007/s00234-005-1344-1

[113] Ostuni JL, Richert ND, Lewis BK, Frank JA. Characterization of differences between multiple sclerosis and normal brain: A global magnetization transfer application. Am J Neuroradiol. 1999;20(3):501–507. 10219419 PMC7056079

[114] Saccenti L, Hagiwara A, Andica C, et al Myelin measurement using quantitative magnetic resonance imaging: A correlation study comparing various imaging techniques in patients with multiple sclerosis. Cells. 2020;9(2):393. 10.3390/cells9020393 PMC7072333 32046340

[115] Siemonsen S, Young KL, Bester M, et al Chronic T2 lesions in multiple sclerosis are heterogeneous regarding phase MR imaging. Clin Neuroradiol. 2016;26(4):457–464. 25895017 10.1007/s00062-015-0389-8

[116] Sled JG, Pike GB. Quantitative imaging of magnetization transfer exchange and relaxation properties in vivo using MRI. Magn Reson Med. 2001;46(5):923–931. 11675644 10.1002/mrm.1278

[117] Smith SA, Farrell JAD, Jones CK, Reich DS, Calabresi PA, van Zijl PCM. Pulsed magnetization transfer imaging with body coil transmission at 3 Tesla: Feasibility and application. Magn Reson Med. 2006;56(4):866–875. 16964602 10.1002/mrm.21035

[118] Yarnykh VL. Fast macromolecular proton fraction mapping from a single off-resonance magnetization transfer measurement. Magn Reson Med. 2012;68(1):166–178. 22190042 10.1002/mrm.23224 PMC3311766

[119] Zhang L, Wen B, Chen T, et al A comparison study of inhomogeneous magnetization transfer (ihMT) and magnetization transfer (MT) in multiple sclerosis based on whole brain acquisition at 3.0 T. Magn Reson Imaging. 2020;70:43–49. 32224092 10.1016/j.mri.2020.03.010

[120] Rocca MA, Mastronardo G, Rodegher M, Comi G, Filippi M. Long-term changes of magnetization transfer-derived measures from patients with relapsing-remitting and secondary progressive multiple sclerosis. Am J Neuroradiol. 1999;20(5):821–827. 10369352 PMC7056141

[121] Duhamel G, Prevost VH, Cayre M, et al Validating the sensitivity of inhomogeneous magnetization transfer (ihMT) MRI to myelin with fluorescence microscopy. Neuroimage. 2019;199:289–303. 31141736 10.1016/j.neuroimage.2019.05.061

[122] Field AS, Samsonov A, Alexander AL, Mossahebi P, Duncan ID. Conventional and quantitative MRI in a novel feline model of demyelination and endogenous remyelination. J Magn Reson Imaging. 2019;49(5):1304–1311. 30302903 10.1002/jmri.26300 PMC6519168

[123] Alsop DC, Detre JA, Golay X, et al Recommended implementation of arterial spin-labeled perfusion MRI for clinical applications: A consensus of the ISMRM perfusion study group and the European consortium for ASL in dementia. Magn Reson Med. 2015;73(1):102–116. 24715426 10.1002/mrm.25197 PMC4190138

[124] Thrippleton MJ, Backes WH, Sourbron S, et al Quantifying blood-brain barrier leakage in small vessel disease: Review and consensus recommendations. Alzheimers Dement. 2019;15(6):840–858. 31031101 10.1016/j.jalz.2019.01.013 PMC6565805

[125] Wilson M, Andronesi O, Barker PB, et al Methodological consensus on clinical proton MRS of the brain: Review and recommendations. Magn Reson Med. 2019;82(2):527–550. 30919510 10.1002/mrm.27742 PMC7179569

[126] Abdel-Fahim R, Mistry N, Mougin O, et al Improved detection of focal cortical lesions using 7 T magnetisation transfer imaging in patients with multiple sclerosis. Mult Scler Relat Disord. 2014;3(2):258–265. 25878014 10.1016/j.msard.2013.10.004

[127] Adusumilli G, Trinkaus K, Sun P, et al Intensity ratio to improve black hole assessment in multiple sclerosis. Mult Scler Relat Disord. 2018;19:140–147. 29223871 10.1016/j.msard.2017.11.020 PMC5803396

[128] Al-Radaideh A, Mougin OE, Lim S-Y, Chou I-J, Constantinescu CS, Gowland P. Histogram analysis of quantitative *T*_1_ and MT maps from ultrahigh field MRI in clinically isolated syndrome and relapsing-remitting multiple sclerosis. NMR Biomed. 2015;28(11):1374–1382. 26346925 10.1002/nbm.3385

[129] Amann M, Papadopoulou A, Andelova M, et al Magnetization transfer ratio in lesions rather than normal-appearing brain relates to disability in patients with multiple sclerosis. J Neurol. 2015;262(8):1909–1917. 26041614 10.1007/s00415-015-7793-5

[130] Audoin B, Au Duong MV, Ranjeva J-P, et al Magnetic resonance study of the influence of tissue damage and cortical reorganization on PASAT performance at the earliest stage of multiple sclerosis. Hum Brain Mapp. 2005;24(3):216–228. 15543553 10.1002/hbm.20083 PMC6871730

[131] Audoin B, Ranjeva J-P, Au Duong MV, et al Voxel-based analysis of MTR images: A method to locate gray matter abnormalities in patients at the earliest stage of multiple sclerosis. J Magn Reson Imaging. 2004;20(5):765–771. 15503338 10.1002/jmri.20178

[132] Bagnato F, Franco G, Ye F, et al Selective inversion recovery quantitative magnetization transfer imaging: Toward a 3 T clinical application in multiple sclerosis. Mult Scler. 2020;26(4):457–467. 30907234 10.1177/1352458519833018 PMC7528886

[133] Bieniek M, Altmann DR, Davies GR, et al Cord atrophy separates early primary progressive and relapsing remitting multiple sclerosis. J Neurol Neurosurg Psychiatry. 2006;77(9):1036–1039. 16793860 10.1136/jnnp.2006.094748 PMC2077733

[134] Brown RA, Narayanan S, Arnold DL. Imaging of repeated episodes of demyelination and remyelination in multiple sclerosis. Neuroimage Clin. 2014;6:20–25. 25610760 10.1016/j.nicl.2014.06.009 PMC4299955

[135] Brown RA, Narayanan S, Stikov N, et al MTR recovery in brain lesions in the BECOME study of glatiramer acetate vs interferon β-1b. Neurology. 2016;87(9):905–911. 27473139 10.1212/WNL.0000000000003043 PMC5035157

[136] Brochet B, Deloire MSA, Bonnet M, et al Should SDMT substitute for PASAT in MSFC? A 5-year longitudinal study. Mult Scler. 2008;14(9):1242–1249. 18653737 10.1177/1352458508094398

[137] Campbell Z, Sahm D, Donohue K, et al Characterizing contrast-enhancing and re-enhancing lesions in multiple sclerosis. Neurology. 2012;78(19):1493–1499. 22539575 10.1212/WNL.0b013e3182553bd2 PMC3345616

[138] Chu SG, Shen TZ, Chen XR. Value of magnetization transfer imaging in judging microchanges lesions in normal-appearing white matter of multiple sclerosis. Zhonghua yi xue za zhi. 2004;84(14):1181–1185. 15387980

[139] Datta G, Colasanti A, Rabiner EA, et al Neuroinflammation and its relationship to changes in brain volume and white matter lesions in multiple sclerosis. Brain. 2017;140(11):2927–2938. 29053775 10.1093/brain/awx228

[140] Davie CA, Silver NC, Barker GJ, et al Does the extent of axonal loss and demyelination from chronic lesions in multiple sclerosis correlate with the clinical subgroup? J Neurol Neurosurg Psychiatry. 1999;67(6):710–715. 10567484 10.1136/jnnp.67.6.710 PMC1736689

[141] Davies GR, Tozer DJ, Cercignani M, et al Estimation of the macromolecular proton fraction and bound pool T2 in multiple sclerosis. Mult Scler. 2004;10(6):607–613. 15584482 10.1191/1352458504ms1105oa

[142] de Jong BA, Huizinga TWJ, Bollen ELEM, et al Production of IL-1β and IL-1Ra as risk factors for susceptibility and progression of relapse-onset multiple sclerosis. J Neuroimmunol. 2002;126(1-2):172–179. 12020968 10.1016/s0165-5728(02)00056-5

[143] De Stefano N, Battaglini M, Stromillo ML, et al Brain damage as detected by magnetization transfer imaging is less pronounced in benign than in early relapsing multiple sclerosis. Brain. 2006;129(Pt 8):2008–2016. 16815879 10.1093/brain/awl152

[144] De Stefano N, Stromillo ML, Rossi F, et al Improving the characterization of radiologically isolated syndrome suggestive of multiple sclerosis. PLoS One. 2011;6(4):e19452. 21559385 10.1371/journal.pone.0019452 PMC3084867

[145] Dehmeshki J, Barker GJ, Tofts PS. Classification of disease subgroup and correlation with disease severity using magnetic resonance imaging whole-brain histograms: Application to magnetization transfer ratios and multiple sclerosis. IEEE Trans Med Imaging. 2002;21(4):320–331. 12022620 10.1109/TMI.2002.1000256

[146] Dehmeshki J, Ruto AC, Arridge S, Silver NC, Miller DH, Tofts PS. Analysis of MTR histograms in multiple sclerosis using principal components and multiple discriminant analysis. Magn Reson Med. 2001;46(3):600–609. 11550255 10.1002/mrm.1233

[147] Dehmeshki J, Silver NC, Leary SM, Tofts PS, Thompson AJ, Miller DH. Magnetisation transfer ratio histogram analysis of primary progressive and other multiple sclerosis subgroups. J Neurol Sci. 2001;185(1):11–17. 11266685 10.1016/s0022-510x(01)00447-6

[148] Deloire MSA, Salort E, Bonnet M, et al Cognitive impairment as marker of diffuse brain abnormalities in early relapsing remitting multiple sclerosis. J Neurol Neurosurg Psychiatry. 2005;76(4):519–526. 15774439 10.1136/jnnp.2004.045872 PMC1739602

[149] Derakhshan M, Caramanos Z, Narayanan S, Arnold DL, Louis Collins D. Surface-based analysis reveals regions of reduced cortical magnetization transfer ratio in patients with multiple sclerosis: A proposed method for imaging subpial demyelination. Hum Brain Mapp. 2014;35(7):3402–3413. 24356893 10.1002/hbm.22410 PMC6869281

[150] Di Perri C, Battaglini M, Stromillo ML, et al Voxel-based assessment of differences in damage and distribution of white matter lesions between patients with primary progressive and relapsing-remitting multiple sclerosis. Arch Neurol. 2008;65(2):236–243. 18268194 10.1001/archneurol.2007.51

[151] Dousset V, Grossman RI, Ramer KN, et al Experimental allergic encephalomyelitis and multiple sclerosis: Lesion characterization with magnetization transfer imaging. Radiology. 1992;182(2):483–491. 1732968 10.1148/radiology.182.2.1732968

[152] Duong M-VA, Audoin B, Boulanouar K, et al Altered functional connectivity related to white matter changes inside the working memory network at the very early stage of MS. J Cerebr Blood F Met. 2005;25(10):1245–1253. 10.1038/sj.jcbfm.9600122 15843789

[153] Faiss JH, Dähne D, Baum K, et al Reduced magnetisation transfer ratio in cognitively impaired patients at the very early stage of multiple sclerosis: A prospective, multicenter, cross-sectional study. BMJ Open. 2014;4(4):e004409. 10.1136/bmjopen-2013-004409 PMC3987712 24722197

[154] Fernando KTM, Tozer DJ, Miszkiel KA, et al Magnetization transfer histograms in clinically isolated syndromes suggestive of multiple sclerosis. Brain. 2005;128(Pt 12):2911–2925. 16219673 10.1093/brain/awh654

[155] Filippi M, Campi A, Dousset V, et al A magnetization transfer imaging study of normal-appearing white matter in multiple sclerosis. Neurology. 1995;45(3 Pt 1):478–482. 7898700 10.1212/wnl.45.3.478

[156] Filippi M, Iannucci G, Tortorella C, et al Comparison of MS clinical phenotypes using conventional and magnetization transfer MRI. Neurology. 1999;52(3):588–594. 10025793 10.1212/wnl.52.3.588

[157] Filippi M, Inglese M, Rovaris M, et al Magnetization transfer imaging to monitor the evolution of MS: A 1-year follow-up study. Neurology. 2000;55(7):940–946. 11061248 10.1212/wnl.55.7.940

[158] Filippi M, Preziosa P, Copetti M, et al Gray matter damage predicts the accumulation of disability 13 years later in MS. Neurology. 2013;81(20):1759–1767. 24122185 10.1212/01.wnl.0000435551.90824.d0

[159] Fisniku LK, Altmann DR, Cercignani M, et al Magnetization transfer ratio abnormalities reflect clinically relevant grey matter damage in multiple sclerosis. Mult Scler. 2009;15(6):668–677. 19435751 10.1177/1352458509103715 PMC3040974

[160] Gallo A, Rovaris M, Benedetti B, et al A brain magnetization transfer MRI study with a clinical follow up of about four years in patients with clinically isolated syndromes suggestive of multiple sclerosis. J Neurol. 2007;254(1):78–83. 17508141 10.1007/s00415-006-0283-z

[161] Ge Y, Grossman RI, Udupa JK, Babb JS, Mannon LJ, McGowan JC. Magnetization transfer ratio histogram analysis of normal-appearing gray matter and normal-appearing white matter in multiple sclerosis. J Comput Assist Tomogr. 2002;26(1):62–68. 11801905 10.1097/00004728-200201000-00009

[162] Giorgio A, Portaccio E, Stromillo ML, et al Cortical functional reorganization and its relationship with brain structural damage in patients with benign multiple sclerosis. Mult Scler. 2010;16(11):1326–1334. 20670979 10.1177/1352458510377333

[163] Gracien R-M, Reitz SC, Hof S-M, et al Assessment of cortical damage in early multiple sclerosis with quantitative T_2_ relaxometry. NMR Biomed. 2016;29(4):444–450. 26820580 10.1002/nbm.3486

[164] Harrison DM, Ratchford JN, Timonium L, Farrell SK, Calabresi PA, Reich DS. Whole brain and tract-specific diffusion tensor and magnetization transfer imaging values correlate with quantified brain atrophy. Neurology. 2010;74(9):A235–A236.

[165] Harrison DM, Caffo BS, Shiee N, et al Longitudinal changes in diffusion tensor-based quantitative MRI in multiple sclerosis. Neurology. 2011;76(2):179–186. 21220722 10.1212/WNL.0b013e318206ca61 PMC3030233

[166] Harrison DM, Shiee N, Bazin P-L, et al Tract-specific quantitative MRI better correlates with disability than conventional MRI in multiple sclerosis. J Neurol. 2013;260(2):397–406. 22886062 10.1007/s00415-012-6638-8 PMC3753185

[167] Hiehle JF Jr, Lenkinski RE, Grossman RI, et al Correlation of spectroscopy and magnetization transfer imaging in the evaluation of demyelinating lesions and normal appearing white matter in multiple sclerosis. Magn Reson Med. 1994;32(3):285–293. 7984060 10.1002/mrm.1910320303

[168] Iannucci G, Tortorella C, Rovaris M, Sormani MP, Comi G, Filippi M. Prognostic value of MR and magnetization transfer imaging findings in patients with clinically isolated syndromes suggestive of multiple sclerosis at presentation. Am J Neuroradiol. 2000;21(6):1034–1038. 10871009 PMC7973902

[169] Jakimovski D, Ramanathan M, Weinstock-Guttman B, et al Higher EBV response is associated with more severe gray matter and lesion pathology in relapsing multiple sclerosis patients: A case-controlled magnetization transfer ratio study. Mult Scler. 2020;26(3):322–332. 30755085 10.1177/1352458519828667 PMC6692251

[170] Jurcoane A, Wagner M, Schmidt C, et al Within-lesion differences in quantitative MRI parameters predict contrast enhancement in multiple sclerosis. J Magn Reson Imaging. 2013;38(6):1454–1461. 23554005 10.1002/jmri.24107

[171] Kalkers NF, Hintzen RQ, Van Waesberghe JHTM, et al Magnetization transfer histogram parameters reflect all dimensions of MS pathology, including atrophy. J Neurol Sci. 2001;184(2):155–162. 11239950 10.1016/s0022-510x(01)00431-2

[172] Kalkers NF, Vrenken H, Uitdehaag BMJ, Polman CH, Barkhof F. Brain atrophy in multiple sclerosis: Impact of lesions and of damage of whole brain tissue. Mult Scler. 2002;8(5):410–414. 12356208 10.1191/1352458502ms833oa

[173] Khalil M, Enzinger C, Langkammer C, et al Cognitive impairment in relation to MRI metrics in patients with clinically isolated syndrome. Mult Scler. 2011;17(2):173–180. 20956399 10.1177/1352458510384009

[174] Laule C, Vavasour IM, Leung E, et al Pathological basis of diffusely abnormal white matter: Insights from magnetic resonance imaging and histology. Mult Scler. 2011;17(2):144–150. 20965961 10.1177/1352458510384008

[175] Laule C, Vavasour IM, Whittall KP, et al Evolution of focal and diffuse magnetisation transfer abnormalities in multiple sclerosis. J Neurol. 2003;250(8):924–931. 12928910 10.1007/s00415-003-1115-z

[176] Lipp I, Jones DK, Bells S, et al Comparing MRI metrics to quantify white matter microstructural damage in multiple sclerosis. Hum Brain Mapp. 2019;40(10):2917–2932. 30891838 10.1002/hbm.24568 PMC6563497

[177] Lipp I, Parker GD, Tallantyre EC, et al Tractography in the presence of multiple sclerosis lesions. Neuroimage. 2020;209:116471. 31877372 10.1016/j.neuroimage.2019.116471 PMC7613131

[178] Liu Z, Pardini M, Yaldizli O, et al Magnetization transfer ratio measures in normal-appearing white matter show periventricular gradient abnormalities in multiple sclerosis. Brain. 2015;138(5):1239–1246. 25823475 10.1093/brain/awv065 PMC5963416

[179] Loevner LA, Grossman RI, Cohen JA, Lexa FJ, Kessler D, Kolson DL. Microscopic disease in normal-appearing white matter on conventional MR images in patients with multiple sclerosis: Assessment with magnetization- transfer measurements. Radiology. 1995;196(2):511–515. 7617869 10.1148/radiology.196.2.7617869

[180] Loevner LA, Grossman RI, McGowan JC, Ramer KN, Cohen JA. Characterization of multiple sclerosis plaques with T1-weighted MR and quantitative magnetization transfer. Am J Neuroradiol. 1995;16(7):1473–1479. 7484636 PMC8338054

[181] Lommers E, Guillemin C, Reuter G, et al Voxel-Based quantitative MRI reveals spatial patterns of grey matter alteration in multiple sclerosis. Hum Brain Mapp. 2021;42:1003–1012. 33155763 10.1002/hbm.25274 PMC7856642

[182] Lommers E, Simon J, Reuter G, et al Multiparameter MRI quantification of microstructural tissue alterations in multiple sclerosis. NeuroImage Clin. 2019;23:101879. 31176293 10.1016/j.nicl.2019.101879 PMC6555891

[183] Mallik S, Muhlert N, Samson RS, et al Regional patterns of grey matter atrophy and magnetisation transfer ratio abnormalities in multiple sclerosis clinical subgroups: A voxel-based analysis study. Multi Scler J. 2015;21(4):423–432. 10.1177/1352458514546513 PMC4390521 25145689

[184] Miki Y, Grossman RI, Udupa JK, et al Differences between relapsing-remitting and chronic progressive multiple sclerosis as determined with quantitative MR imaging. Radiology. 1999;210(3):769–774. 10207480 10.1148/radiology.210.3.r99mr44769

[185] Mistry N, Abdel-Fahim R, Mougin O, Tench C, Gowland P, Evangelou N. Cortical lesion load correlates with diffuse injury of multiple sclerosis normal appearing white matter. Mult Scler. 2014;20(2):227–233. 23858017 10.1177/1352458513496344

[186] Nantes JC, Proulx S, Zhong J, et al GABA and glutamate levels correlate with MTR and clinical disability: Insights from multiple sclerosis. Neuroimage. 2017;157:705–715. 28131894 10.1016/j.neuroimage.2017.01.033

[187] Nantes JC, Zhong J, Holmes SA, Narayanan S, Lapierre Y, Koski L. Cortical damage and disability in multiple sclerosis: Relation to intracortical inhibition and facilitation. Brain Stimul. 2016;9(4):566–573. 27053387 10.1016/j.brs.2016.01.003

[188] Oh J, Sotirchos ES, Saidha S, et al Relationships between quantitative spinal cord MRI and retinal layers in multiple sclerosis. Neurology. 2015;84(7):720–728. 25609766 10.1212/WNL.0000000000001257 PMC4336102

[189] Ozturk A, Smith SA, Gordon-Lipkin EM, et al MRI of the corpus callosum in multiple sclerosis: Association with disability. Mult Scler. 2010;16(2):166–177. 20142309 10.1177/1352458509353649 PMC2820126

[190] Papanikolaou N, Papadaki E, Karampekios S, et al T2 relaxation time analysis in patients with multiple sclerosis: Correlation with magnetization transfer ratio. Eur Radiol. 2004;14(1):115–122. 14600774 10.1007/s00330-003-1946-0

[191] Pardini M, Yaldizli O, Sethi V, et al Motor network efficiency and disability in multiple sclerosis. Neurology. 2015;85(13):1115–1122. 26320199 10.1212/WNL.0000000000001970 PMC4603887

[192] Penny SA, Summers MM, Swanton JK, Cipolotti L, Miller DH, Ron MA. Changing associations between cognitive impairment and imaging in multiple sclerosis as the disease progresses. J Neuropsychiatry Clin Neurosci. 2013;25(2):134–140. 23686031 10.1176/appi.neuropsych.11090218

[193] Phillips MD, Grossman RI, Miki Y, et al Comparison of T2 lesion volume and magnetization transfer ratio histogram analysis and of atrophy and measures of lesion burden in patients with multiple sclerosis. Am J Neuroradiol. 1998;19(6):1055–1060. 9672011 PMC8338648

[194] Pike GB, de Stefano N, Narayanan S, Francis GS, Antel JP, Arnold DL. Combined magnetization transfer and proton spectroscopic imaging in the assessment of pathologic brain lesions in multiple sclerosis. Am J Neuroradiol. 1999;20(5):829–837. 10369353 PMC7056133

[195] Pike GB, De Stefano N, Narayanan S, et al Multiple sclerosis: Magnetization transfer MR imaging of white matter before lesion appearance on T2-weighted images. Radiology. 2000;215(3):824–830. 10831705 10.1148/radiology.215.3.r00jn02824

[196] Pinter D, Khalil M, Pichler A, et al Predictive value of different conventional and non-conventional MRI-parameters for specific domains of cognitive function in multiple sclerosis. Neuroimage Clin. 2015;7:715–720. 25844323 10.1016/j.nicl.2015.02.023 PMC4375639

[197] Ramani A, Dalton C, Miller DH, Tofts PS, Barker GJ. Precise estimate of fundamental in-vivo MT parameters in human brain in clinically feasible times. Magn Reson Imaging. 2002;20(10):721–731. 12591568 10.1016/s0730-725x(02)00598-2

[198] Ranjeva J-P, Franconi J-M, Manelfe C, Berry I. Magnetization transfer with echo planar imaging. Magn Reson Mater Phys Biol Med. 1997;5(4):259–265. 10.1007/BF02595043 9440826

[199] Raz E, Pantano P. Diffusion tensor imaging in multiple sclerosis: Longitudinal changes. Fut Neurol. 2011;6(3):335–338.

[200] Reich DS, Ozturk A, Calabresi PA, Mori S. Automated vs. conventional tractography in multiple sclerosis: Variability and correlation with disability. Neuroimage. 2010;49(4):3047–3056. 19944769 10.1016/j.neuroimage.2009.11.043 PMC2843834

[201] Reich DS, Smith SA, Gordon-Lipkin EM, et al Damage to the optic radiation in multiple sclerosis is associated with retinal injury and visual disability. Arch Neurol. 2009;66(8):998–1006. 19667222 10.1001/archneurol.2009.107 PMC2784485

[202] Reich DS, Smith SA, Zackowski KM, et al Multiparametric magnetic resonance imaging analysis of the corticospinal tract in multiple sclerosis. Neuroimage. 2007;38(2):271–279. 17870615 10.1016/j.neuroimage.2007.07.049 PMC2082136

[203] Reich DS, Zackowski KM, Gordon-Lipkin EM, et al Corticospinal tract abnormalities are associated with weakness in multiple sclerosis. Am J Neuroradiol. 2008;29(2):333–339. 17974617 10.3174/ajnr.A0788 PMC2802714

[204] Rocca MA, Mesaros S, Pagani E, Sormani MP, Comi G, Filippi M. Thalamic damage and long-term progression of disability in multiple sclerosis. Radiology. 2010;257(2):463–469. 20724544 10.1148/radiol.10100326

[205] Roostaei T, Sadaghiani S, Mashhadi R, et al Convergent effects of a functional C3 variant on brain atrophy, demyelination, and cognitive impairment in multiple sclerosis. Mult Scler. 2019;25:532–540. 29485352 10.1177/1352458518760715

[206] Rovaris M, Agosta F, Sormani MP, et al Conventional and magnetization transfer MRI predictors of clinical multiple sclerosis evolution: A medium-term follow-up study. Brain. 2003;126(Pt 10):2323–2332. 12937086 10.1093/brain/awg232

[207] Rovaris M, Bozzali M, Rodegher M, Tortorella C, Comi G, Filippi M. Brain MRI correlates of magnetization transfer imaging metrics in patients with multiple sclerosis. J Neurol Sci. 1999;166(1):58–63. 10465501 10.1016/s0022-510x(99)00113-6

[208] Rovaris M, Bozzali M, Santuccio G, et al Relative contributions of brain and cervical cord pathology to multiple sclerosis disability: A study with magnetisation transfer ratio histogram analysis. J Neurol Neurosurg Psychiatry. 2000;69(6):723–727. 11080222 10.1136/jnnp.69.6.723 PMC1737158

[209] Rovaris M, Filippi M, Falautano M, et al Relation between MR abnormalities and patterns of cognitive impairment in multiple sclerosis. Neurology. 1998;50(6):1601–1608. 9633700 10.1212/wnl.50.6.1601

[210] Rovaris M, Holtmannspötter M, Rocca MA, et al Contribution of cervical cord MRI and brain magnetization transfer imaging to the assessment of individual patients with multiple sclerosis: A preliminary study. Mult Scler. 2002;8(1):52–58. 11936489 10.1191/1352458502ms772oa

[211] Samson RS, Cardoso MJ, Muhlert N, et al Investigation of outer cortical magnetisation transfer ratio abnormalities in multiple sclerosis clinical subgroups. Mult Scler. 2014;20(10):1322–1330. 24552746 10.1177/1352458514522537

[212] Samson RS, Muhlert N, Sethi V, et al Sulcal and gyral crown cortical grey matter involvement in multiple sclerosis: A magnetisation transfer ratio study. Mult Scler Relat Disord. 2013;2(3):204–212. 25877727 10.1016/j.msard.2013.01.001

[213] Sharma J, Zivadinov R, Jaisani Z, et al A magnetization transfer MRI study of deep gray matter involvement in multiple sclerosis. J Neuroimaging. 2006;16(4):302–310. 17032378 10.1111/j.1552-6569.2006.00054.x

[214] Silver N, Lai M, Symms M, Barker G, McDonald I, Miller D. Serial gadolinium-enhanced and magnetization transfer imaging to investigate the relationship between the duration of blood-brain barrier disruption and extent of demyelination in new multiple sclerosis lesions. J Neurol. 1999;246(8):728–730. 10460455 10.1007/s004150050442

[215] Tipirneni A, Weinstock-Guttman B, Ramanathan M, et al MRI characteristics of familial and sporadic multiple sclerosis patients. Mult Scler. 2013;19(9):1145–1152. 23232600 10.1177/1352458512469697

[216] Tjoa CW, Benedict RHB, Dwyer MG, Carone DA, Zivadinov R. Regional specificity of magnetization transfer imaging in multiple sclerosis. J Neuroimaging. 2008;18(2):130–136. 18315593 10.1111/j.1552-6569.2007.00198.x

[217] Tortorella C, Viti B, Bozzali M, et al A magnetization transfer histogram study of normal-appearing brain tissue in MS. Neurology. 2000;54(1):186–193. 10636146 10.1212/wnl.54.1.186

[218] Tozer D, Ramani A, Barker GJ, Davies GR, Miller DH, Tofts PS. Quantitative magnetization transfer mapping of bound protons in multiple sclerosis. Magn Reson Med. 2003;50(1):83–91. 12815682 10.1002/mrm.10514

[219] Tozer DJ, Davies GR, Altmann DR, Miller DH, Tofts PS. Correlation of apparent myelin measures obtained in multiple sclerosis patients and controls from magnetization transfer and multicompartmental T2 analysis. Magn Reson Med. 2005;53(6):1415–1422. 15906291 10.1002/mrm.20479

[220] Tozer DJ, Marongiu G, Swanton JK, Thompson AJ, Miller DH. Texture analysis of magnetization transfer maps from patients with clinically isolated syndrome and multiple sclerosis. J Magn Reson Imaging. 2009;30(3):506–513. 19711400 10.1002/jmri.21885

[221] Traboulsee A, Dehmeshki J, Peters KR, et al Disability in multiple sclerosis is related to normal appearing brain tissue MTR histogram abnormalities. Mult Scler. 2003;9(6):566–573. 14664468 10.1191/1352458503ms958oa

[222] van Buchem MA, Grossman RI, Armstrong C, et al Correlation of volumetric magnetization transfer imaging with clinical data in MS. Neurology. 1998;50(6):1609–1617. 9633701 10.1212/wnl.50.6.1609

[223] van Buchem MA, Udupa JK, McGowan JC, et al Global volumetric estimation of disease burden in multiple sclerosis based on magnetization transfer imaging. Am J Neuroradiol. 1997;18(7):1287–1290. 9282856 PMC8338026

[224] van Waesberghe JHTM, Castelijns JA, Lazeron RHC, Lycklama a Nijeholt GJ, Barkhof F. Magnetization transfer contrast (MTC) and long repetition time spin-echo MR imaging in multiple sclerosis. Magn Reson imaging. 1998;16(4):351–358. 9665545 10.1016/s0730-725x(97)00310-x

[225] Van Buchem MA, McGowan JC, Kolson DL, Polansky M, Grossman RI. Quantitative volumetric magnetization transfer analysis in multiple sclerosis: Estimation of macroscopic and microscopic disease burden. Magn Reson Med. 1996;36(4):632–636. 8892218 10.1002/mrm.1910360420

[226] Vavasour IM, Laule C, Li DKB, Traboulsee AL, MacKay AL. Is the magnetization transfer ratio a marker for myelin in multiple sclerosis? J Magn Reson Imaging. 2011;33(3):710–718. 10.1002/jmri.22441 21563257

[227] Vavasour IM, Li DKB, Laule C, Traboulsee AL, Moore GRW, Mackay AL. Multi-parametric MR assessment of T(1) black holes in multiple sclerosis: Evidence that myelin loss is not greater in hypointense versus isointense T(1) lesions. J Neurol. 2007;254(12):1653–1659. 17934875 10.1007/s00415-007-0604-x

[228] Vavasour IM, Whittall KP, MacKay AL, Li DKB, Vorobeychik G, Paty DW. A comparison between magnetization transfer ratios and myelin water percentages in normals and multiple sclerosis patients. Magn Reson Med. 1998;40(5):763–768. 9797161 10.1002/mrm.1910400518

[229] Vrenken H, Geurts JJ, Knol DL, et al Normal-appearing white matter changes vary with distance to lesions in multiple sclerosis. Am J Neuroradiol. 2006;27(9):2005–2011. 17032884 PMC7977884

[230] Vrenken H, Pouwels PJW, Ropele S, et al Magnetization transfer ratio measurement in multiple sclerosis normal-appearing brain tissue: Limited differences with controls but relationships with clinical and MR measures of disease. Mult Scler. 2007;13(6):708–716. 17613597 10.1177/1352458506075521

[231] Wang Y, Sun P, Wang Q, et al Differentiation and quantification of inflammation, demyelination and axon injury or loss in multiple sclerosis. Brain. 2015;138(Pt 5):1223–1238. 25724201 10.1093/brain/awv046 PMC4407189

[232] Weinstock-Guttman B, Benedict RHB, Tamano-Blanco M, et al The rs2030324 SNP of brain-derived neurotrophic factor (BDNF) is associated with visual cognitive processing in multiple sclerosis. Pathophysiology. 2011;18(1):43–52. 20547043 10.1016/j.pathophys.2010.04.005

[233] Wu GF, Schwartz ED, Lei T, et al Relation of vision to global and regional brain MRI in multiple sclerosis. Neurology. 2007;69(23):2128–2135. 17881718 10.1212/01.wnl.0000278387.15090.5a

[234] Yaldizli O, Pardini M, Sethi V, et al Characteristics of lesional and extra-lesional cortical grey matter in relapsing-remitting and secondary progressive multiple sclerosis: A magnetisation transfer and diffusion tensor imaging study. Mult Scler. 2016;22(2):150–159. 26014608 10.1177/1352458515586085

[235] Yaldizli O, Sethi V, Pardini M, et al HLA-DRB*1501 associations with magnetic resonance imaging measures of grey matter pathology in multiple sclerosis. Mult Scler Relat Disord. 2016;7:47–52. 27237756 10.1016/j.msard.2016.03.003

[236] Yaldizli O, Sethi V, Pardini M, et al Response to the commentary of Yates RL and DeLuca GC on the study: HLA-DRB1*1501 associations with magnetic resonance imaging measures of grey matter pathology in multiple sclerosis. Mult Scler Relat Disord. 2018;19:168–170. 29409599 10.1016/j.msard.2016.08.006

[237] Yarnykh VL, Bowen JD, Samsonov A, et al Fast whole-brain three-dimensional macromolecular proton fraction mapping in multiple sclerosis. Radiology. 2015;274(1):210–220. 25208343 10.1148/radiol.14140528 PMC4314118

[238] Zheng Y, Lee J-C, Rudick R, Fisher E. Long-term magnetization transfer ratio evolution in multiple sclerosis white matter lesions. J Neuroimaging. 2018;28(2):191–198. 29076591 10.1111/jon.12480

[239] Zhong J, Nantes JC, Holmes SA, Gallant S, Narayanan S, Koski L. Abnormal functional connectivity and cortical integrity influence dominant hand motor disability in multiple sclerosis: A multimodal analysis. Hum Brain Mapp. 2016;37(12):4262–4275. 27381089 10.1002/hbm.23307 PMC6867582

[240] Zivadinov R, Raj B, Ramanathan M, et al Autoimmune comorbidities are associated with brain injury in multiple sclerosis. Am J Neuroradiol. 2016;37(6):1010–1016. 26892983 10.3174/ajnr.A4681 PMC7963547

[241] Anik Y, Demirci A, Efendi H, Bulut SSD, Celebi I, Komsuoglu S. Evaluation of normal appearing white matter in multiple sclerosis: Comparison of diffusion magnetic resonance, magnetization transfer imaging and multivoxel magnetic resonance spectroscopy findings with expanded disability status scale. Clin Neuroradiol. 2011;21(4):207–215. 21847666 10.1007/s00062-011-0091-4

[242] Battiston M, Schneider T, Grussu F, et al Fast bound pool fraction mapping via steady-state magnetization transfer saturation using single-shot EPI. Magn Reson Med. 2019;82(3):1025–1040. 31081239 10.1002/mrm.27792

[243] Bomboi G, Ikonomidou VN, Pellegrini S, et al Quality and quantity of diffuse and focal white matter disease and cognitive disability of patients with multiple sclerosis. J Neuroimaging. 2011;21(2):e57–e63. 20626570 10.1111/j.1552-6569.2010.00488.x

[244] Campi A, Filippi M, Comi G, Scotti G, Gerevini S, Dousset V. Magnetisation transfer ratios of contrast-enhancing and nonenhancing lesions in multiple sclerosis. Neuroradiology. 1996;38(2):115–119. 8692418 10.1007/BF00604792

[245] Coombs BD, Best A, Brown MS, et al Multiple sclerosis pathology in the normal and abnormal appearing white matter of the corpus callosum by diffusion tensor imaging. Multi Scler. 2004;10:392–397. 10.1191/1352458504ms1053oa 15327035

[246] Dworkin JD, Sweeney EM, Schindler MK, Chahin S, Reich DS, Shinohara RT. PREVAIL: Predicting recovery through estimation and visualization of active and incident lesions. Neuroimage Clin. 2016;12:293–299. 27551666 10.1016/j.nicl.2016.07.015 PMC4983640

[247] Dwyer M, Bergsland N, Hussein S, Durfee J, Wack D, Zivadinov R. A sensitive, noise-resistant method for identifying focal demyelination and remyelination in patients with multiple sclerosis via voxel-wise changes in magnetization transfer ratio. J Neurol Sci. 2009;282(1–2):86–95. 19386319 10.1016/j.jns.2009.03.016

[248] Filippi M, Bozzali M, Comi G. Magnetization transfer and diffusion tensor MR imaging of basal ganglia from patients with multiple sclerosis. J Neurol Sci. 2001;183(1):69–72. 11166797 10.1016/s0022-510x(00)00471-8

[249] Filippi M, Campi A, Martinelli V, Pereira C, Scotti G, Comi G. Transitional progressive multiple sclerosis: MRI and MTI findings. Acta Neurol Scand. 1995;92(2):178–182. 7484070 10.1111/j.1600-0404.1995.tb01036.x

[250] Filippi M, Rocca MA, Mastronardo G, Comi G. Lesion load measurements in multiple sclerosis: The effect of incorporating magnetization transfer contrast in fast-FLAIR sequence. Magn Reson Imaging. 1999;17(3):459–461. 10195590 10.1016/s0730-725x(98)00182-9

[251] Filippi M, Rocca MA, Pagani E, et al European study on intravenous immunoglobulin in multiple sclerosis: Results of magnetization transfer magnetic resonance imaging analysis. Arch Neurol. 2004;61(9):1409–1412. 15364687 10.1001/archneur.61.9.1409

[252] Filippi M, Tortorella C, Rovaris M, et al Changes in the normal appearing brain tissue and cognitive impairment in multiple sclerosis. J Neurol Neurosurg Psychiatry. 2000;68(2):157–161. 10644780 10.1136/jnnp.68.2.157 PMC1736794

[253] Fox RJ, Fisher E, Tkach J, Lee J-C, Cohen JA, Rudick RA. Brain atrophy and magnetization transfer ratio following methylprednisolone in multiple sclerosis: Short-term changes and long-term implications. Mult Scler. 2005;11(2):140–145. 15794385 10.1191/1352458505ms1142oa

[254] Fox RJ, Kivisakk P, Fisher E, et al Multiple sclerosis: Chemokine receptor expression on circulating lymphocytes in correlation with radiographic measures of tissue injury. Mult Scler. 2008;14(8):1036–1043. 18701575 10.1177/1352458508092261

[255] Furby J, Hayton T, Altmann D, et al Different white matter lesion characteristics correlate with distinct grey matter abnormalities on magnetic resonance imaging in secondary progressive multiple sclerosis. Mult Scler. 2009;15(6):687–694. 19435748 10.1177/1352458509103176

[256] Gass A, Davie CA, Barker GJ, McDonald WI, Miller DH. Demonstration of plaque development in multiple sclerosis using magnetisation transfer imaging and short echo time proton spectroscopy. Nervenarzt. 1997;68(12):996–1001. 9465344 10.1007/s001150050229

[257] Grimaud J, Barker GJ, Wang L, et al Correlation of magnetic resonance imaging parameters with clinical disability in multiple sclerosis: A preliminary study. J Neurol. 1999;246(10):961–967. 10552247 10.1007/s004150050491

[258] Hayton T, Furby J, Smith KJ, et al Clinical and imaging correlates of the multiple sclerosis impact scale in secondary progressive multiple sclerosis. J Neurol. 2012;259(2):237–245. 21863390 10.1007/s00415-011-6151-5

[259] Hayton T, Furby J, Smith KJ, et al Longitudinal changes in magnetisation transfer ratio in secondary progressive multiple sclerosis: Data from a randomised placebo controlled trial of lamotrigine. J Neurol. 2012;259(3):505–514. 21904901 10.1007/s00415-011-6212-9

[260] Hazra A, Reich BJ, Reich DS, Shinohara RT, Staicu A-M. A spatio-temporal model for longitudinal image-on-image regression. Stat Biosci. 2019;11(1):22–46. 31156722 PMC6537615

[261] Iannucci G, Minicucci L, Rodegher M, Sormani MP, Comi G, Filippi M. Correlations between clinical and MRI involvement in multiple sclerosis: Assessment using T1, T2 and MT histograms. J Neurol Sci. 1999;171(2):121–129. 10581378 10.1016/s0022-510x(99)00259-2

[262] Inglese M, van Waesberghe JHTM, Rovaris M, et al The effect of interferon beta-1b on quantities derived from MT MRI in secondary progressive MS. Neurology. 2003;60(5):853–860. 12629246 10.1212/01.wnl.0000049929.27032.29

[263] Koenig KA, Sakaie KE, Lowe MJ, et al Hippocampal volume is related to cognitive decline and fornicial diffusion measures in multiple sclerosis. Magn Reson Imaging. 2014;32(4):354–358. 24512796 10.1016/j.mri.2013.12.012 PMC4025957

[264] Laule C, Vavasour IM, Kolind SH, et al Long T2 water in multiple sclerosis: What else can we learn from multi-echo T2 relaxation? J Neurol. 2007;254(11):1579–1587. 17762945 10.1007/s00415-007-0595-7

[265] Lema A, Bishop C, Malik O, et al A comparison of magnetization transfer methods to assess brain and cervical cord microstructure in multiple sclerosis. J Neuroimaging. 2017;27(2):221–226. 27491693 10.1111/jon.12377

[266] Levesque I, Sled JG, Narayanan S, et al The role of edema and demyelination in chronic T1 black holes: A quantitative magnetization transfer study. J Magn Reson Imaging. 2005;21(2):103–110. 15666408 10.1002/jmri.20231

[267] Mainero C, De Stefano N, Iannucci G, et al Correlates of MS disability assessed in vivo using aggregates of MR quantities. Neurology. 2001;56(10):1331–1334. 11376183 10.1212/wnl.56.10.1331

[268] Maranzano J, Dadar M, Rudko DA, et al Comparison of multiple sclerosis cortical lesion types detected by multicontrast 3 T and 7 T MRI. Am J Neuroradiol. 2019;40(7):1162–1169. 31221631 10.3174/ajnr.A6099 PMC7048547

[269] Maranzano J, Dadar M, Zhernovaia M, Arnold DL, Collins DL, Narayanan S. Automated separation of diffusely abnormal white matter from focal white matter lesions on MRI in multiple sclerosis. Neuroimage. 2020;213:116690. 32119987 10.1016/j.neuroimage.2020.116690

[270] Narayanan S, Francis SJ, Sled JG, et al Axonal injury in the cerebral normal-appearing white matter of patients with multiple sclerosis is related to concurrent demyelination in lesions but not to concurrent demyelination in normal-appearing white matter. Neuroimage. 2006;29(2):637–642. 16126413 10.1016/j.neuroimage.2005.07.017

[271] Newbould RD, Nicholas R, Thomas CL, et al Age independently affects myelin integrity as detected by magnetization transfer magnetic resonance imaging in multiple sclerosis. Neuroimage Clin. 2014;4:641–648. 24936415 10.1016/j.nicl.2014.02.004 PMC4053639

[272] Otaduy MCG, Callegaro D, Bacheschi LA, Leite CC. Correlation of magnetization transfer and diffusion magnetic resonance imaging in multiple sclerosis. Mult Scler. 2006;12(6):754–759. 17263003 10.1177/1352458506070824

[273] Pardini M, Sudre CH, Prados F, et al Relationship of grey and white matter abnormalities with distance from the surface of the brain in multiple sclerosis. J Neurol Neurosurg Psychiatry. 2016;87(11):1212–1217. 27601434 10.1136/jnnp-2016-313979

[274] Riva M, Ikonomidou VN, Ostuni JJ, et al Tissue-specific imaging is a robust methodology to differentiate in vivo T1 black holes with advanced multiple sclerosis-induced damage. Am J Neuroradiol. 2009;30(7):1394–1401. 19406765 10.3174/ajnr.A1573 PMC5613286

[275] Roostaei T, Sadaghiani S, Mashhadi R, et al Convergent effects of a functional C3 variant on brain atrophy, demyelination, and cognitive impairment in multiple sclerosis. Mult Scler. 2019;25(4):532–540. 29485352 10.1177/1352458518760715

[276] Rovaris M, Filippi M, Minicucci L, et al Cortical/subcortical disease burden and cognitive impairment in patients with multiple sclerosis. AJNR Am J Neuroradiol. 2000;21(2):402–408. 10696031 PMC7975362

[277] Rudko DA, Derakhshan M, Maranzano J, Nakamura K, Arnold DL, Narayanan S. Delineation of cortical pathology in multiple sclerosis using multi-surface magnetization transfer ratio imaging. Neuroimage Clin. 2016;12:858–868. 27872808 10.1016/j.nicl.2016.10.010 PMC5107650

[278] Santos AC, Narayanan S, de Stefano N, et al Magnetization transfer can predict clinical evolution in patients with multiple sclerosis. J Neurol. 2002;249(6):662–668. 12111296 10.1007/s00415-002-0686-4

[279] Siger-Zajdel M, Selmaj K. Magnetisation transfer ratio analysis of normal appearing white matter in patients with familial and sporadic multiple sclerosis. J Neurol Neurosurg Psychiatry. 2001;71(6):752–756. 11723195 10.1136/jnnp.71.6.752 PMC1737649

[280] Silver NC, Lai M, Symms MR, Barker GJ, McDonald WI, Miller DH. Serial magnetization transfer imaging to characterize the early evolution of new MS lesions. Neurology. 1998;51(3):758–764. 9748023 10.1212/wnl.51.3.758

[281] Summers MM, Fisniku LK, Anderson VM, Miller DH, Cipolotti L, Ron MA. Cognitive impairment in relapsing—remitting multiple sclerosis can be predicted by imaging performed several years earlier. Mult Scler. 2008;14(2):197–204. 17986503 10.1177/1352458507082353

[282] van Waesberghe JHT, Castelijns JA, Scheltens P, et al Comparison of four potential MR parameters for severe tissue destruction in multiple sclerosis lesions. Magn Reson Imaging. 1997;15(2):155–162. 9106143 10.1016/s0730-725x(96)00340-2

[283] van Waesberghe JH, van Buchem MA, Filippi M, et al MR outcome parameters in multiple sclerosis: Comparison of surface-based thresholding segmentation and magnetization transfer ratio histographic analysis in relation to disability (a preliminary note). Am J Neuroradiol. 1998;19(10):1857–1862. 9874537 PMC8337743

[284] vanWaesberghe JHTM, Castelijns JA, Roser W, et al Single-dose gadolinium with magnetization transfer versus triple-dose gadolinium in the MR detection of multiple sclerosis lesions. Am J Neuroradiol. 1997;18(7):1279–1285. 9282855 PMC8338043

[285] van Waesberghe JH, van Walderveen MA, Castelijns JA, et al Patterns of lesion development in multiple sclerosis: Longitudinal observations with T1-weighted spin-echo and magnetization transfer MR. Am J Neuroradiol. 1998;19(4):675–683. 9576653 PMC8337386

[286] Weinstock-Guttman B, Ramanathan M, Hashmi K, et al Increased tissue damage and lesion volumes in African Americans with multiple sclerosis. Neurology. 2010;74(7):538–544. 20089944 10.1212/WNL.0b013e3181cff6fb

[287] Zivadinov R, Dwyer MG, Hussein S, et al Voxel-wise magnetization transfer imaging study of effects of natalizumab and IFNβ-1a in multiple sclerosis. Mult Scler. 2012;18(8):1125–1134. 22194217 10.1177/1352458511433304

