# Supplementary material for: Quantitative magnetization transfer imaging in relapsing-remitting multiple sclerosis: a systematic review and meta-analysis
Source: Brain Commun. 2022 Apr 4;4(2):fcac088. doi: 10.1093/braincomms/fcac088 (PMC9149789; doi:10.1093/braincomms/fcac088)
Supplement: fcac088_Supplementary_Data [file fcac088_Supplementary_Data.docx]

**Supplementary Materials**

|  |  | ***Pages*** |
| --- | --- | --- |
| **Supplementary Results** |  | 2-4 |
| **Supplementary Tables** |  | 5-63 |
| Supplementary Table 1 | Mixed MS subtype studies | 5-11 |
| Supplementary Table 2 | Relapse-onset MS studies | 12-15 |
| Supplementary Table 3 | Included studies: Sample characteristics | 16-32 |
| Supplementary Table 4 | Included studies: Disease duration, clinical disability, MT metrics & brain regions | 33-48 |
| Supplementary Table 5 | Included studies: DMT usage | 49 |
| Supplementary Table 6 | Longitudinal change in MTR across all brain sub-regions | 50 |
| Supplementary Table 7 | Longitudinal change in MTR in normal-appearing brain tissue | 51 |
| Supplementary Table 8 | Longitudinal change in MTR in normal-appearing white matter | 52 |
| Supplementary Table 9 | Longitudinal change in MTR in lesions | 53 |
| Supplementary Table 10 | Included studies: Risk of bias | 54-63 |
| **Supplementary Figures** |  | 64-69 |
| Supplementary Figure 1 | Meta-analysis: brain sub-regions | 64 |
| Supplementary Figure 2 | Longitudinal evolution of MTR | 65 |
| Supplementary Figure 3 | Longitudinal evolution of MTR in different lesion types | 66 |
| Supplementary Figure 4 | Funnel plots: RRMS vs healthy control, MTR | 67 |
| Supplementary Figure 5 | Funnel plots: MTR and clinical disability | 68 |
| Supplementary Figure 6 | Funnel plots: RRMS vs healthy controls, compartmental models | 69 |

**Supplementary Results**

MTI Acquisition Protocol Parameters

**Magnetic field strength**

MTI was mainly performed at 1.5T^11,36-40,42-48,50,57-59,63,65,70-80,82-88,93,95,96,98,100,102,103,108-110,112,113,116^ (k=50, Figure 2A). Recent studies were acquired at 7T,^64^ 3T (k=29),^35,41,49,51-56,62,66-69,89,90,94,97,101,104-107,111,114,115,117-119^ both 3T and 1.5T,^99^ or 4T.^60^ Field strength was occasionally unreported (k=4), although 1.5T may be assumed in such cases due to publication dates or multi-centre approaches.^61,81,91,92^

**MTI Sequence**

**Pulse sequence**

In the majority of MTI protocols (k=60), gradient echo (GRE) was used, either as a 2D (k=13),^36,43-48,50,63,80,84,98,103^ 3D (k=27),^11,35,41,49,61,62,64,65,68,73,76,78,79,82,83,86,89,94,97,99,101,107-109,111,112,114,117-119^ both 2D and 3D (k=1),^85^ or unspecified (k=16)^51-56,58,66,67,77,81,90,93,100,115,116^ GRE sequence. Eight studies used 2D spin echo (SE, k=8)^37-40,42,57,88,95^ and one study employed both SE and GRE.^104^ The pulse sequence was not described in six studies.^59,69,72,75,91,92^

**Image contrast: TR, TE and excitation flip angle**

Proton density (PD)-weighting (with and without an MT pulse) was typically used for MTI. Nevertheless, some studies adopted T1-weighting^49,67,70,71,95^ or T2*-weighting.^51-56^ The TR and TE varied accordingly with the intended weighting and sequence type. For example, TR ranged from 2.67 ms for a MT-sensitized balanced steady-state free precession sequence (bSSFP) at 1.5T^85^ to 3000 ms for a 2D pulsed inhomogeneous MT HASTE (Half-fourier Acquisition Single-shot Turbo spin-Echo),^88^ with a median TR of 65.50 ms (k=74/86). The median TE was 11.70 ms (k=73, range: 1.23 ms^51-56^ to 90 ms^37-40,57^). Some studies did not, however, report TR (k=12)^58,60,64,72,75,86,91,92,96,104-106^ or TE (k=13).^58,60,64,72,75,77,86,91-93,96,114,116^

The excitation flip angle ranged from 3° for a 3D FLASH (fast low-angle shot) acquisition^112^ to 90° for 3D selective inversion recovery (SIR)-turbo SE & 3D SIR-EPI (echo planar imaging),^104^ T1-weighted 2D SE^95^ and PD-weighted MT sequences,^69^ with a median of 15° (k=55). Many studies (k=31) did not report the excitation flip angle.^37-40,42,51-55,57,58,60,62,64,70-72,74,75,81,88,91,92,96,102,105-107,110,113^

**Voxel Size, Slice Thickness and Number of Slices**

The median in-plane voxel size was 1.0mm by 1.0mm (k=73 & k=66/86 respectively, range: 0.7mm^36^ to 2.2mm^104,105,117^). The median slice thickness was 4.0 mm (k=73/86, range: 1mm^35,41^ to 9mm^88^ ^11,35-43,46-57,59,61-71,73,74,76-78,80-86,88-90,93,95-111,113-118^). The median number of slices acquired was 28 (k=61, range: 1^77,78,93,105,106,116^ to 192^114^ ^35,37-44,46,48-50,52-57,59,60,63-68,70,71,74,76-78,80-82,85-90,93,96,97,100,102-108,111,113-119^) with resulting coverage of 126mm (median, k=55, range: 5mm^78,105,106^ to 280mm^82^).

**MT Pulse Characteristics**

**Radiofrequency Pulse Frequency**

The vast majority of studies achieved selective saturation of the `bound’ pool with a radiofrequency pulse at an offset or multiple offsets from the water proton frequency (Figure 2B). When a single offset was used (k=59/86), the median frequency was 1500 Hz (range: 600 Hz^71^ to 7000 Hz^88^). When multiple offset frequencies were considered to permit quantitative model construction or due to inter-centre variability, the range was wider (100 Hz to 80 kHz, k=7).^77,78,93,94,100,116,118^

Alternative approaches included SIR with a low-power on-resonance pulse^104,106^ and FastPACE two-point T1 mapping with a 1-2-1 binomial on-resonance pulse.^87,96^ SIR aims to invert the `free’ water while leaving the `bound’ pool relatively unaffected. Quantitative SIR MTI parameters are estimated from the bi-exponential recovery of the `free’ water signal, sampled at various inversion times. Two studies used on-resonance pulse MTI but did not report further details^65,81^ and fourteen studies did not report the offset frequency.^42,49,55,56,64,72,75,79,91,92,95,99,101,105^

**Flip angle of MT Pulse**

Amongst studies that reported the MT pulse as a flip angle, the median angle was 500° (k=34) with range 200°^53^ to 1430°.^37,39,40,57^ When only pulse peak amplitude was reported, the range was 3.4μT^45,46,48^ to 23.6μT,^87^ with a median of 7μT (k=15). Four studies used multiple pulse energies^77,93,116,118^ and a number of studies (k=33) did not report the pulse flip angle or power.^36,39,42,49,55,56,59-62,64,65,69,71,72,74,75,79,82,84,91,92,94,95,99-102,104-107,113^

**Shape of MT Pulse**

The radiofrequency pulse shape was generally Gaussian (k=28),^11,35,41,43-48,50,61,63,65,67,68,73,80,83-85,89,94,103,111,112,114,115^ although Sinc (k=6),^66,76,78,79,109,110^ Sinc-Gaussian (k=5),^62,97,117,118^ Fermi (k=4),^86,107,108,119^ 1-2-1 binomial (k=2),^87,96^ and hyperbolic secant pulses (k=1)^60^ were also used. Forty studies did not specify pulse shape.

**Pulse Duration**

The radiofrequency pulse duration was related to the type of MT sequence and ranged from 0.08 ms for MT-sensitized bSSFP^85^ to 700 ms for 2D-pulsed-ihMT HASTE sequence.^88^ Median MT pulse duration was 10.15 ms (k=61).

Supplementary Tables

***Supplementary Table 1: Overview of studies which were excluded from the final selection due to mixed multiple sclerosis (MS) subtypes.*** *CIS: clinically-isolated syndrome; MT: magnetisation transfer; MTR: MT ratio; MWF: myelin water fraction; PPMS: primary progressive MS; PSR: pool size ratio; qMT: quantitative MT; RRMS: relapsing-remitting MS; SPMS: secondary progressive MS.*Included in meta-analyses*

| **Citation** | **Mixed Analyses** | **Subgroup Analyses** | **RRMS (n)** | **SPMS (n)** | **PPMS (n)** | **Other (n)** | **Healthy (n)** | **MT Metric** |
| --- | --- | --- | --- | --- | --- | --- | --- | --- |
| Abdel-Fahim et al., (2014)^126^ | • | - | 16 | 1 | 1 | - | 9 | MTR |
| Adusumilli et al., (2018)^127^ | • | - | 13 | 10 | 15 | - | - | MTR; IR |
| *Agosta et al., (2006)^5^ | - | • | 34 | 19 | - | 20 (CIS) | 16 | MTR |
| Al-Radaideh et al., (2015)^128^ | - | • | 11 | - | - | 17 (CIS) | 22 | MTR (-/+) |
| Amann et al., (2015)^129^ | • | - | 59 | 12 | - | - | - | MTR |
| Audoin et al., (2005)^130^ | • | - | - | - | - | 18 (CIS) | 18 | MTR |
| Audoin et al., (2004)^131^ | • | - | - | - | - | 18 (CIS) | 18 | MTR |
| Bagnato et al., (2020)^132^ | • | - | 11 | 3 | - | 4 (CIS) | 9 | PSR; R1_f_ |
| Bieniek et al., (2006)^133^ | - | • | 37 | - | 43 | - | 58 | MTR |
| Brown et al., (2014)^134^ | - | • | 18 | 88 | - | - | - | MTR |
| Brown et al., (2016)^135^ | • | - | 75 | | | | - | Fat sat. MTR |
| Brochet et al., (2008)^136^ | - | • | 56 (46) | 0 (8) | - | - | 56 | MTR |
| Campbell et al., (2012)^137^ | • | - | 77 | 8 | 3 | - | - | MTR |
| Chu et al., (2004)^138^ | • | - | 41 | | | | 21 | MTR |
| Datta et al., (2017)^139^ | - | • | 14 | 7 | - | - | - | MTR |
| Davie et al., (1999)^140^ | • | - | 3 | 10 | | 5 (benign) | - | MTR |
| *Davies et al., (2004)^141^ | - | • | 22 | 6 | 4 | 1 (benign) | 27 | *f*; T2_b_ |
| de Jong et al., (2002)^142^ | • | - | 18 | 32 | 25 | - | 129 | MTR |
| *De Stefano et al., (2006)^143^ | - | • | 50 | - | - | 50 (benign) | 10 | MTR |
| *De Stefano et al., (2011)^144^ | - | • | 20 | - | - | 19 (RIS) | 20 | MTR |
| Dehmeshki et al., (2002)^145^ | - | • | 10 | 16 | 46 | 11 (benign) | 39 | MTR |
| *Dehmeshki, Ruto et al., (2001)^146^ | - | • | 11 | 11 | 10 | 10 (benign) | 9 | MTR |
| *Dehmeshki, Silver et al., (2001)^147^ | - | • | 10 | 16 | 46 | 11 (benign) | 39 | MTR |
| *Deloire et al., (2005) ^148^ | - | • | 44 | - | - | - | 44 | MTR |
| *Derakhshan et al., (2014)^149^ | - | • | 12 | 12 | - | - | 12 | MTR |
| *Di Perri et al., (2008)^150^ | - | • | 36 | - | 24 | - | 23 | MTR |
| Dousset et al., (1992)^151^ |  | • | 10 | 5 | | - | 5 | MTR |
| Duoung et al., (2005)^152^ | • | - | - | - | - | 18 (CIS) | 18 | MTR |
| Faiss et al., (2014)^153^ | • | - | 11 | - | - | 36 (CIS) | - | MTR |
| Fernando et al., (2005)^154^ | • | - | - | - | - | 100 (CIS) | 50 | MTR |
| *Filippi et al., (1995)^155^ | - | • | 20 | - | - | 7 (chronic progressive) | 10 | MTR |
| *Filippi et al., (1999)^156^ | - | • | 7 | 7 | - | - | 5 | MTR |
| Filippi et al., (2000)^157^ | - | • | 39 | 19 | 9 | 9 (benign); 20 (CIS) | 20 | MTR |
| Filippi et al., (2013)^158^ | • | - | 34 (32) | 19 (16) | - | 20 (19) [CIS] | - | MTR |
| *Fisniku et al., (2009)^159^ | - | • | 31 | 10 | - | 28 (CIS) | 19 | MTR |
| Gallo et al. (2007)^160^ | • | - | - | - | - | 43 (CIS) | 22 | MTR |
| *Ge et al. (2002)^161^ | - | • | 16 | 11 | - | - | 16 | MTR |
| Giorgio et al. (2010)^162^ | - | • | 10 | - | - | 25 (benign) | 10 | MTR |
| Gracien et al. (2016)^163^ | • | - | - | - | - | 12 (CIS & RRMS) | 12 | MTR |
| *Harrison et al., (2010)^164^ | - | • | 66 | 30 | 21 | - | 26 | MTR |
| Harrison et al., (2011)^165^ | - | • | 40 | 24 | 14 | - | - | MTR |
| Harrison et al., (2013)^166^ | - | • | 66 | 30 | 21 | - | 26 | MTR |
| *Hiehle Jr et al., (1994)^167^ | - | • | 10 | 1 | | - | - | MTR |
| Iannucci et al., (2000)^168^ | • | - | - | - | - | 24 (CIS) | 20 | MTR |
| *Jakimovski et al., (2020)^169^ | - | • | 69 | 32 | - | - | 41 | MTR |
| *Jurcoane et al., (2013)^170^ | - | • | 17 | 12 | 3 | - | 17 | MTR |
| *Kalkers et al., (2001)^171^ | - | • | 16 | 26 | 26 | 11 (benign) | 23 | MTR |
| Kalkers et al., (2002)^172^ | - | • | 22 | 32 | 32 | - | - | MTR |
| *Khalil et al., (2011)^173^ | - | • | 80 | - | - | 44 (CIS) | - | MTR |
| Laule et al., (2011)^174^ | • | - | 38 | 13 | 1 | 1 (benign) | - | MTR; (MWF) |
| Laule et al., (2003)^175^ | • | - | 7 | 1 | 1 | - |  | MTR |
| Lipp et al., (2019)^176^ | • | - | 83 | - | 22 | 27 | - | MTR; (MWF) |
| Lipp et al., (2020)^177^ | • | - | 105 [29] | - | 26 [0] | - | 19 | MTR |
| *Liu et al., (2015)^178^ | - | • | 43 | 28 | - | - | 38 | MTR |
| *Loevner, Grossman, Cohen et al., (1995)^179^ | - | • | 14 | 9 | - | - | 9 | MTR |
| Loevner, Grossman, McGowan et al., (1995)^180^ | • | - | 13 | 4 | - | - | - | MTR |
| Lommers et al., (2020)^181^ | • | - | 14 | 7 | 14 | - | 36 | MTsat |
| *Lommers et al., (2019)^182^ | - | • | 15 | 21 | | - | 36 | MTsat |
| *Mallik et al., (2015)^183^ | - | • | 51 | 28 | 19 | - | 29 | MTR |
| *Miki et al., (1999)^184^ | - | • | 26 | 12 | | - | - | MTR |
| Mistry et al., (2014)^185^ | • | - | 16 | 2 | 1 | - | - | MTR |
| Nantes et al., (2017)^186^ | • | - | 27 | 14 | 6 | 21 | - | MTR |
| Nantes et al., (2016)^187^ | - | • | 22 | 8 | 6 | - | 18 | MTR |
| Oh et al., (2015)^188^ | - | • | 66 | 36 | | - | 11 | MTR |
| Ozturk et al., (2010)^189^ | • | - | 35 | 20 | 14 | 29 | - | MTR |
| Papanikolaou et al., (2004)^190^ | • | - | 8 | 3 | 2 | - | - | MTR |
| *Pardini et al., (2015)^191^ | - | • | 44 | 27 | - | - | 22 | MTR |
| Penny et al., (2013)^192^ | • | - | 32 | 2 | - | 19 (CIS); 8 | - | MTR |
| Phillips et al., (1998)^193^ | • | - | 20 | 10 | | - | 8 | MTR |
| *Pike et al., (1999)^194^ | - | • | 11 | 14 | 5 | - | - | MTR |
| Pike et al., (2000)^195^ | - | • | 11 | 14 | 5 | - | 12 | MTR |
| Pinter et al., (2015)^196^ | • | - | 47 | 5 | - | 17 (CIS) | - | MTR |
| Ramani et al., (2002)^197^ | • | - | 3 | - | - | 3 (benign) | 5 | qMT inc. *f*,T2_b_ |
| Ranjeva et al., (1997)^198^ | - | • | 2 | - | 1 | - | 3 | MTR |
| Raz et al., (2011)^199^ | • | - | 40 | 24 | 14 | - | - | MTR |
| Reich et al., (2010)^200^ | • | - | 50 | 24 | 14 | - | 27 | MTR |
| Reich et al., (2009)^201^ | - | • | 52 | 24 | 14 | - | 29 | MTR |
| Reich et al., (2007)^202^ | • | - | 43 | 22 | 10 | - | 29 | MTR |
| Reich et al., (2008)^203^ | - | • | 26 | 13 | 8 | - | 29 | MTR |
| *Rocca et al., (1999)^120^ | - | • | 7 | 7 | - | - | 5 | MTR |
| Rocca et al., (2010)^204^ | - | • | 34 | 19 | - | 20 (CIS) | 13 | MTR |
| Roostaei et al., (2018)^205^ | - | • | 161 | | - | - | - | MTR |
| Rovaris et al., (2003)^206^ | - | • | 34 | 19 | - | 20 (CIS) | 16 | MTR |
| Rovaris et al., (1999) ^207^ | • | - | 25 | 17 | - | - | - | MTR |
| *Rovaris et al., (2000) ^208^ | - | • | 40 | 28 | 9 | - | - | MTR |
| Rovaris et al., (1998) ^209^ | • | - | 10 | 15 | 5 | - | - | MTR |
| Rovaris et al., (2002) ^210^ | - | • | 39 | - | 25 | - | 20 | MTR |
| *Samson et al., (2014)^211^ | - | • | 44 | 25 | 19 | - | 35 | MTR |
| *Samson et al., (2013)^212^ | - | • | 31 | 14 | 16 | - | 32 | MTR |
| *Sharma et al., (2006)^213^ | - | • | 50 | 10 | - | - | 20 | MTR |
| Silver et al., (1999)^214^ | • | - | 1 | 2 | - | - | - | MTR |
| Tipirneni et al., (2013)^215^ | • | - | 477 | 222 | 30 | 29 (CIS) | - | MTR |
| Tjoa et al., (2008)^216^ | - | • | 28 | 17 | - | - | 19 | MTR |
| *Tortorella et al., (2000)^217^ | - | • | 33 | 20 | 13 | 11 (benign) | 20 | MTR |
| Tozer et al., (2003)^218^ | • | - | 14 | 2 | 2 | 2 (benign) | 7 | MTR; qMT inc. *f*, T2_b_ |
| Tozer et al., (2005)^219^ | • | - | 10 | 5 | 4 | - | 9 | qMT inc. T2_b_, *f* |
| Tozer et al., (2009)^220^ | • | - | 32 | - | 3 | 38 (CIS) | 23 | MTR |
| *Traboulsee et al., (2003)^221^ | - | • | 70 | 25 | - | - | 63 | MTR |
| van Buchem et al., (1998)^222^ | • | - | 28 | 16 | | - | - | MTR |
| van Buchem et al., (1997)^223^ | • | - | 6 | 5 | | - | 11 | MTR |
| van Waesberghe et al., (1998)^224^ | • | - | 8 | 11 | 1 | - | 5 | SNR & CNR with/  without MT |
| van Buchem et al., (1996) ^225^ | - | • | 11 | 4 | | - | - | MTR |
| Vavasour et al., (2011)^226^ | • | - | 5 | 1 | 1 | - | 7 | MTR |
| Vavasour et al., (2007)^227^ | • | - | 35 | 19 | 12 | - | 23 | MTR |
| *Vavasour et al., (1998)^228^ | - | • | 5 | 4 | - | - | 10 | MTR; MWF |
| *Vrenken et al., (2006)^229^ | - | • | 34 | 18 | 11 | - | 22 | MTR |
| *Vrenken et al., (2007)^230^ | - | • | 35 | 19 | 12 | - | 23 | MTR |
| Wang et al., (2015)^231^ | • | - | 3 | 2 | 1 | - | 5 | MTR |
| Weinstock-Guttman et al., (2011)^232^ | • | - | 145 | | | - | - | MTR |
| Wu et al., (2007)^233^ | • | - | 42 | 3 | | - | - | MTR |
| *Yaldizli, Pardini et al., (2016)^234^ | - | • | 46 | 26 | - | - | 36 | MTR |
| Yaldizli, Sethi et al., (2016)^235^ | • | - | 30 | 30 | 25 | - | 36 | MTR |
| Yaldizli et al., (2018)^236^ | • | - | 30 | 30 | 25 | - | - | MTR |
| *Yarnykh et al., (2015)^237^ | - | • | 18 | 12 | - | - | 14 | MTR; R1; MPF |
| Zheng et al., (2018)^238^ | - | • | 32 | 17 | - | 10 (CIS) | 14 | MTR |
| Zhong et al., (2016)^239^ | • | - | 27 | 12 | 4 | - | 20 | MTR |
| Zivadinov et al., (2016)^240^ | • | - | 554 | 227 | 34 | - | - | MTR |

*Supplementary Table 2: Overview of studies excluded from final analysis as relapse-onset multiple sclerosis (MS), not solely relapsing-remitting MS. MT: magnetisation transfer; MTR: MT ratio; MTsat: MTsat; MWF: myelin water fraction; PSR: pool size ratio; qMT: quantitative MT; RRMS: relapsing-remitting MS; SPMS: secondary progressive MS.*

| **Citation** | **Subgroup MTI Analyses** | **SPMS only** | **RRMS (n)** | **SPMS (n)** | **Healthy (n)** | **MT Technique** |
| --- | --- | --- | --- | --- | --- | --- |
| Anik et al. (2011)^241^ | - | - | 27 | 3 | 30 | MTR |
| Battiston et al. (2019)^242^ | - | • | - | 1 | 6 | qMT inc. T2_b_, PSR |
| Bomboi et al. (2011)^243^ | - | - | 20 | 4 | 24 | MTR |
| Campi et al. (1996)^244^ | • | - | 21 | 8 | 10 | MTR |
| Coombs et al. (2004)^245^ | - | - | 8 | 2 | 12 [8] | MTR |
| Dworkin et al. (2016)^246^ | -- | - | 27 | 5 | - | MTR |
| Dwyer et al. (2009)^247^ | • | - | 19 | 11 | 15 | MTR |
| Filippi et al. (2001)^248^ | -- | - | 17 | 14 | 14 | MTR |
| Filippi et al. (1995)^249^ | - | • | - | 1 | - | MTR |
| Filippi et al. (1999)^250^ | - | - | 15 | 5 | - | MTR; CNR |
| Filippi et al. (2004)^251^ | - | • | - | 72 | - | MTR |
| Filippi et al. (2000)^252^ | - | - | 8 | 11 | 20 | MTR |
| Fox et al. (2005)^253^ | • | - | 13 | 6 | - | MTR |
| Fox et al. (2008)^254^ | - | - | 36 | 19 | - | MTR |
| Furby et al. (2009)^255^ | - | • | - | 117 | - | MTR |
| Gass et al. (1997)^256^ | - | • | - | 1 | - | MTR |
| Grimaud et al. (1999)^257^ | - | - | 9 | 6 | - | MTR |
| Hayton et al. (2012)^258^ | - | • | - | 118 | - | MTR |
| Hayton et al. (2012)^259^ | - | • | - | 117 | - | MTR |
| Hazra et al. (2019)^260^ | - | - | 46 | | - | MTR |
| Iannucci et al. (1999)^261^ | - | - | 44 | 28 | 20 | MTR |
| Inglese et al. (2003)^262^ | - | • | - | 82 | - | MTR |
| Koenig et al. (2014)^263^ | - | - | 45 | 7 | 20 | MTR |
| Laule et al. (2007)^264^ | - | - | 14 (1 benign) | 5 | - | MTR; (MWF) |
| Lema et al. (2017)^265^ | - | - | 128 | 6 | - | MTR; MTsat |
| Levesque et al. (2005)^266^ | - | - | 5 | 5 | - | F; MTR; PD_f_ |
| Mainero et al. (2001)^267^ | - | - | 21 | 2 | - | MTR |
| Maranzano et al. (2019)^268^ | - | - | 10 | 10 | - | MTR |
| Maranzano et al. (2020)^269^ | - | - | 10/13/NA | 10/7/67 | - | MTR |
| Narayanan et al. (2006)^270^ | - | - | 4 | 4 | 5 | F |
| Newbould et al. (2014)^271^ | - | - | 36 | 2 | 11 | MTR |
| Otaduy et al. (2006)^272^ | - | - | 11 | 3 | - | MTR |
| Pardini et al. (2016)^273^ | - | - | 41 | 26 | 30 | MTR |
| Riva et al. (2009)^274^ | - | - | 9 | 7 | - | MTR |
| Roostaei et al. (2018)^275^ | - |  | 161 | | - | MTR |
| Rovaris et al. (2000)^276^ | - | - | 8 | 14 | - | MTR |
| Rudko et al. (2016)^277^ | - | - | 21 | 4 | 12 | MTR |
| Santos et al. (2002)^278^ | - | - | 8 | 10 | 12 | MTR |
| Siger-Zajdel et al. (2001)^279^ | - | - | 12 | 8 | 10 | MTR |
| Silver et al. (1998)^280^ | - | - | 1 | 2 | - | MTR |
| Summers et al. (2008)^281^ | - | - | 30 (29) | - (1) | - | MTR |
| Van Waesberghe et al. (1997)^282^ | - | - | 5 | 5 | - | MTR |
| Van Waesberghe et al. (1998)^283^ | - | - | 8 | 33 | - | MTR |
| Van Waesberghe et al. (1997)^284^ | - | - | 16 | 5 | - | MT with contrast ratio |
| Van Waesberghe et al. (1998)^285^ | - | - | 8 | 3 | - | MTR |
| Weinstock-Guttman et al. (2010)^286^ | - | - | 211 | 74 | - | MTR |
| Zivadinov et al. (2012)^287^ | - | - | 73 | 30 | 22 | MTR |

**Supplementary Table 3: Study sample characteristics (n=86) for relapsing-remitting multiple sclerosis patients and controls.** Square brackets indicate data for recruited participants which may differ from participants in final analysis. RRMS: relapsing-remitting multiple sclerosis; F:M: female-to-male; DMTs: disease-modifying therapies; wks: weeks; mths: months; yrs: years; IfN: interferon; PegIfN: pegylated interferon; i.v.: intraveneous; s.c.: subcutaneous. ♣ median age, mean not reported.

| **Citation** | **Location of Study Centre(s)** | **N** | | **Sex ratio**  **(F:M)** | | **Age**  **(mean, in yrs)** | | **DMTs & Steroids** |
| --- | --- | --- | --- | --- | --- | --- | --- | --- |
|  |  | **RRMS** | **HC** | **RRMS** | **HC** | **RRMS** | **HC** |  |
| Al-Radaideh, Athamneh, et al. (2020) | Zarqa, Jordan | 30 | 30 | 1.31 | 1.14 | 31.27 | 32.35 | No corticosteroid treatment for 4 wks preceeding study |
| Amann, Sprenger, et al. (2015) | Basel | 27 [31] | - | 1.58 | - | 54.4 | - | All unknown DMTs; no corticosteroid 3mths prior |
| Arnold, Gold, et al. (2017) | International | 392 [540] | - | [3.39] | - | [38.4] | - | Delayed-release dimethyl fumarate (i.e. BG-12, 240mg, 3 OR 2 times daily) OR placebo |
| Arnold, Calabresi, et al. (2014) | International | 858 [1512] | - | [2.43] | - | [36.5] | - | PegIfN-β1a (s.c., 125µg every 2wks) OR PegIfN-β1a (s.c., 125µg every 4wks) OR placebo (then treat as above at 48wks) |
| Audoin, Davies, et al. (2007) | London /  Marseille | 38 | 45 | 2.8 | 1.37 | 36.3 | 34 | Unknown |
| Bellmann-Strobl, Stiepani, et al. (2009) | Berlin | 6 [17] | 17 | [1.43] | 1.43 | 33 | 30.5 | IfN-β1a (s.c., 22µg, 3 times a week) |
| Bernitsas, Kopinsky, et al. (2020) | Detroit, MI | 44 [50] | 27 | 1.75 | 2.38 | 40.8 | 37.2 | daily .5mg fingolimod with 30 day washout period for IfN-β / glatiramer acetate; steroid-free for at least 1 month. No monoclonal antibodies (e.g. alemtuzumab, natalizumab, rituximab, daclizumab) |
| Bonnier, Roche, et al. (2014) | Lausanne | 36 | 18 | 2.0 | 1.0 | 34.8 | 33 | 30 IfN-β or fingolimod for 3+ months |
| Bonnier, Roche, et al. (2015) | Lausanne | 36 | 18 | 2.0 | 1.0 | 34.8 | 33 | 30 IfN-β or fingolimod for 3+ months |
| Bonnier, Marechal, et al. (2017) | Lausanne | 23 | 9 | 1.88 | 1.25 | 35.7 | 34.3 | 20 IfN-β or fingolimod for 3+ mths (22 at 2yrs); no corticosteroids 3mths prior |
| Bonnier, Fischi-Gomez, et al. (2019) | Lausanne | 15 | 32 | 2.0 | 1.5 | 32 | 39.56 | No corticosteroid therapy for at least 3 mth prior. High dosage of either IfN-β or fingolimod |
| Catalaa, Grossman, et al. (2000) | Pennsylvania | 23 | 25 | 6.33 | - | - | - | No immunosuppressant/  immunomodulatory drugs at time of MRI, except for corticosteroids for exacerbations |
| Cercignani, Iannucci, et al. (2000) | Milan | 35 | 24 | 1.33 | 1.67 | 28 | 29 | No immunomodulatory/ immunosuppressantdrugs for 1yr prior and during study; no steroid for 3mths prior |
| Cercignani, Basile, et al. (2009) | Rome | 13 | 14 | 1.60 | 0.75 | 39.4 | 34.9 | 9 IfN-β; 4 glatiramer acetate; no steroids 3 mth prior |
| Codella, Rocca, et al. (2002) | Milan | 28 | 30 | 2.11 | 1.25 | 37.6/39.1 | 38.6 | Untreated |
| Colasanti, Guo, et al. (2014) | London | 11 | 11 | 10.0 | 0.83 | 45.1 | 45.7 | 2 natalizumab; 6 IfN-β; 3 none |
| Cronin, Xu, et al. (2020) | Nashville | 2 | 8 | - | 0.14 | - | 29.9 | Unknown |
| Davies, Ramani, et al. (2003) | London | 5 | 5 | 1.50 | 1.50 | 40 | 38 | Unknown |
| Davies, Ramio-Torrenta, et al. (2004) | London | 38 | 35 | 2.80 | 1.19 | 36.3 | 38.5 | No DMT at time of imaging |
| Davies, Altmann, Hadjiprocopis et al. (2005) | London | 21 [23] | 14 [19] | [4.75] | - | 37 | 34 | Untreated at baseline; 7 on IfN-β by 1yr |
| Davies, Altmann, Rashid, et al. (2005) | London | 22 [23] | 10 [19] | [4.75] | [1.1] | 37 | 34 | Untreated at baseline; 7 on IfN-β by 1yr |
| Deloire, Ruet, et al. (2011) | Bordeaux | 44 | 56 | 3.40 | 1.80 | 44 | 38.2 | 95.6% DMTs |
| De Stefano, Narayanan, et al. (2002) | Montreal/  Siena | 57 [60] | 35 | 2.16 | 1.48 | 35 | 35 | None for 1 mth prior |
| Dortch, Li, et al. (2011) | Nashville | 2 | 9 | 1.00 | 2.00 | - | - | Unknown |
| Dortch, Bagnato, et al. (2018) | Nashville | 1 | 4 | - | 1.00 | 37 | - | Unknown |
| Ernst, Chang, et al. (1998) | Torrance, CA | 1 | - | - | - | 20 | - | IfN-β1b, 0.25mg every other day; 3 day course of methylprednisolone (1 g/d, i.v.) |
| Fatemidokht, Harirchian, et al. (2020) | Tehran | 18 | - | 0.50 | - | 37 ^♣^ | - | Unknown |
| Fazekas, Ropele, et al. (2002) | Graz | 12 | - | 2.00 | - | - | - | Oral IfN-β1a OR placebo |
| Filippi, Rocca, et al. (1998) | Milan | 10 | - | 2.33 | - | 30.4 | - | Methylprednisolone for relapses but >10 days prior to MRI; no other immunomodulatory or immunosuppressants |
| Filippi, Rocca, et al. (1999) | Milan | 10 | - | 2.33 | - | 30.4 | - | Methylprednisolone for relapses but >10 days prior to MRI; no other immunomodulatory or immunosuppressants |
| Filippi, Rocca, et al. (2014) | International | 63 [92] | - | - | - | - | - | 38 (31 at 24mths) laquinomod; 37 (32 at 24 mths) placebo |
| Fooladi, Sharini, et al. (2018) | Tehran | 30 | 30 | 1.73 | 2.33 | 30.2 | 30 | Unknown |
| Fooladi, Riyahi Alam, et al. (2020) | Tehran | 12 | 12 | 2.00 | 3.00 | 31 | 29.5 | Immunomodulatory therapy |
| Fritz, Keller, et al. (2017) | Baltimore | 29 [30] | 29 | 1.42 | 2.50 | 48.69 | 50.76 | No corticosteroids for 30 days prior to testing |
| Frohman, Dwyer, et al. (2009) | Buffalo/  Dallas | 12 | 4 | 1.40 | 1.00 | 40.6 | 35.3 | None for 1 month prior |
| Ge, Grossman, et al. (2001) | Philadelphia | 18 | 18 | 5.00 | 2.00 | 34.1 | 33.9 | Unknown |
| Ge, Grossman, et al. (2003) | New York | 22 | - | 2.67 | - | 35.2 | - | No immunomodulatory treatment previously but short course of steroids for relapses |
| Giacomini, Levesque, et al. (2009) | Montreal | 6 | - | - | - | 43.7 | - | 1 untreated; 1 natalizumab; 2 glatiramer acetate; 1 IfN-βa (s.c.); 1 IfN-βb |
| Goodkin, Rooney, et al. (1998) | San Francisco | 11 [22] | 11 | 1.00 | 0.83 | 34.3 | 38.1 | No immunomodulatory therapy or immunosuppressantdrugs; MRI >14 days after steroids |
| Gracien, Jurcoane, et al. (2016) | Frankfurt | 22 | 10 | 4.50 | 4.00 | 34.7 | 34.2 | 3 natalizumab; 1 fingolimod; 1 dimethyl fumarate; 1 glatiramer acetate; 14 untreated; 2 unknown |
| Griffin, Chard, et al. (2002) | London | 22 | 11 | 2.14 | 1.75 | 36.6 | 37 | No DMTs; no steroids for 1 mth prior |
| Guo, Jewells, et al. (2001) | Durham | 12 | - | 2.00 | - | 39 | - | Unknown |
| Helms, Dathe, et al. (2008) | Gottingen | 1 | 7 | - | - | 27 | - | Unknown |
| Iannucci, Rovaris, et al. (2001) | Milan | 34 | 15 | 1.62 | 1.50 | 34.8 | 34 | Unknown |
| Kamagata, Zalesky, et al. (2019) | Tokyo | 14 | 14 | - | 14.0 | 42.8 | 43.2 | Unknown |
| Karampekios, Papanikolaou et al. (2005) | Crete | 12 | 5 | 1.40 | 1.50 | 27.2 | 27.6 | Unknown |
| Kita, Goodkin, et al. (2000) | San Francisco | 8 [22] | - | 1.00 | - | 38 | - | IfN-β1a; no methylprednisolone 14 days prior to MRI |
| Kuhle, Barro, et al. (2016) | Lausanne | 19 [31] | 18 | [1.82] | 1.25 | 32 | 31 | Untreated at baseline; at follow-up, 11 IfN-β1a; 1 IfN-β1b; 4 glatiramer acetate; 6 fingolimod |
| Levesque, Giacomini, et al. (2010) | Montreal | 5 | 5 | - | 0.67 | 42.6 | - | 1 glatiramer acetate; 4 untreated |
| Lin, Tench, et al. (2008) | Nottingham | 36 | 13 | 2.60 | 1.60 | 37.5 | 34 | Unknown |
| Mangia, Carpenter, et al. (2014) | Minneapolis | 9 | 7 | 3.50 | 2.50 | 38 | 37 | Unknown |
| McKeithan, Lyttle, et al. (2019) | Nashville | 19 | 37 | 3.75 | 2.08 | 38 | 32 | Unknown |
| Mesaros, Rocca, et al. (2010) | International | 42 | - | 1.80 | - | 36.7 | - | Placebo |
| Miller, Fox, et al. (2015) | International | 555 [758] | - | 1.71 | - | 37.5 | - | Delayed-release dimethyl fumarate (240mg 2 or 3 times daily) OR glatiramer acetate (20mg, daily) OR placebo |
| Muhlert, Atzori, et al. (2014) | London | 14 [18] | 17 | 0.50 | 0.55 | 43.5 | 39.7 | No corticosteroids fro 4 wks prior; treatment unknown |
| O’Muircheart-aigh, Vavasour, et al. (2019) | Vancouver | 24 [56] | 38 | 1.67 | 1.92 | 37 | 35 | Ocrelizumab or IfN-β1a |
| Oreja-Guevara, Charil, et al. (2006) | Milan | 22 | - | 2.14 | - | 36.6 | - | Untreated |
| Ostuni, Richert, et al. (1999) | Bethesda | 9 | 5 | 3.50 | 0.67 | 37 | 37 | Unknown |
| Patel, Grossman, et al. (1999) | Philadelphia | 20 | - | 4.00 | - | 37 | - | 8 steroid treatment ~1mth for exacerbation; 3 received IfN-α |
| Preziosa, Pagani, et al. (2020) | Milan | 52 [104] | - | 1.36 | - | 36.85 | - | 24 fingolimod; 28 natalizumab |
| Reich, White, et al. (2015) | Bethesda | 6 [12] | - | 0.20 | - | 35 | - | IfN-β; 2 glatiramer acetate; 8 untreated |
| Reitz, Hof, et al. (2017) | Frankfurt | 9 | 12 | 2.00 | 0.71 | - | - | 3 IfN-β1a; 1 glatiramer acetate; 2 natalizumab; 1 fingolimod; 2 dimethyl fumarate |
| Richert, Ostuni, et al. (1998) | Bethesda | 8 | 5 | 1.00 | 1.00 | 37 | 37.2 | IfN-β1b 8mU, s.c., every other day |
| Richert, Ostuni, et al. (2001) | Bethesda | 4 | - | 1.00 | - | 29 | - | IfN-β1a; methylprednidolone (corticosteroid i.v.) |
| Rocca, Falini, et al. (2002) | Milan | 14 | 15 | 1.33 | 1.50 | 37.6 | 38.6 | No steroid for 6 mths prior; never treated with immunomodulatory drugs or immunosuppressants |
| Romascano, Meskaldji, et al. (2015) | Lausanne | 28 | 16 | 1.80 | 1.29 | 34.32 | 33.06 | 28 IfN-β or fingolimod for 3+ months |
| Ropele, Strasser-Fuchs, et al. (2000) | Graz | 9 | 8 | 2.00 | 1.00 | 38 | - | 5 on long-term immunomodulatory therapy |
| Rovira, Alonso, et al. (1999) | Barcelona | 11 | 6 | 0.22 | - | 32 | - | No prior cytotoxic or immunomodulatory therapy; no corticosteroids during study |
| Rudick, Lee, et al. (2006) | Cleveland | 30 | - | - | - | 36.3 | - | Initially placebo or IfN-β1a; DMTs changed over time but also untreated periods |
| Saccenti, Hagiwara, et al. (2020) | Tokyo | 21 [37] | - | 9.50 | - | 37.9 | - | Unknown |
| Schwartz, Tagge, et al. (2019) | Oregon  (7 sites) | 1 | 0 | - | - | 45 | - | Dimethyl fumarate |
| Siemonsen, Young, et al. (2016) | Hamburg | 23 | - | 1.30 | - | 41 | - | Unknown |
| Sled, Pike (2001) | Montreal/  Siena | 1 | 2 | - | - | - | - | Unknown |
| Smith, Farrell, et al. (2006) | Baltimore | 5 | 9 | - | - | - | 30 | Unknown |
| Thaler, Faizy, et al. (2018) | Hamburg | 35 | - | 1.69 | - | 37 | - | Dimethyl fumarate |
| Van den Elskamp, Knol, et al. (2010) | International | 24 [32] | - | 1.67 | - | 32.3 | - | Oral IfN-β1a OR placebo |
| Van Obberghen, McHinda, et al. (2018) | Marseille | 25 | 20 | 4.00 | 2.33 | 41.5 | 40.5 | 10 DMTs |
| Weinstock-Guttman, Zivadinov, et al. (2007) | Buffalo | 39 [52] | - | 4.57 | - | 49.6 | - | Unknown (maybe IfN-β1a), no steroid for 4wks prior |
| Yarnykh (2012) | Seattle | 2 | 2 | - | 2.00 | 42.5 | 47 | Unknown |
| Yiannakas, Tozer, et al. (2013) | London | 10 | - | 2.33 | - | 53 | - | Unknown |
| Zhang, Wen, et al. (2020) | Baoji, Shaanxi | 18 | 16 | 2.00 | 1.67 | 31.2 | 30.4 | Unknown |
| Zhou, Zhu, et al. (2004) | Lyon/Chatres/  Milan | 10 | 10 | 1.50 | 1.33 | 38.5 | 31.4 | No steroid treatment for 3 mths prior |
| Zivadinov, De Masi, et al. (2001) | Trieste | 63 | 30 | 2.15 | - | 35.4 | - | No immunomodulatory drugs/steroids 3 mths prior |
| Zivadinov, Hussein, et al. (2011) | Buffalo | 13 [19] | 16 | 2.80 | 15.0 | 42.3 | 44.2 | Glatiramer acetate (20mg/day sc) |
| Zivadinov, Ramanathan, et al. (2012) | Buffalo | 47 | - | 3.27 | - | 45.5 | - | IfN-β1a (i.m., 30µg) |
| Zivadinov, Dwyer, et al. (2014) | Buffalo | 21 | 15 | 2.00 | 1.14 | 39.9 | 36.7 | IfN-β1a (s.c., 44µg) |

**Supplementary Table 4: *Overview of disease duration and clinical disability in relapsing-remitting multiple sclerosis cohorts with magnetisation transfer imaging.*** *mean; ♣ median. MT: magnetisation transfer; EDSS: Expanded Disability Status Scale score; GM: grey matter; WM: white matter; NAWM: normal-appearing WM; NAGM: normal-appearing GM; WB: whole brain; NAWB: normal-appearing WB; MT: magnetisation transfer; MTR: ratio; ihMTR: inhomogeneous MTR; qihMT: quantitative inhomogeneous MT; MWF: myelin water fraction; F: the relative size of the macromolecular pool relative to the free pool; f: macromolecular proton fraction; T2A: transverse relaxation rate of the free pool (inverse of T1f); kf: (forward) exchange rate from free to bound pool; MMC: the relative macromolecular content, similar to F; T1sat: T1 relaxation time under an MT pulse; SD: standard deviation.

| **Citation** | **Disease Duration (yrs)** | **EDSS (at baseline)** | **MT Metric** | **Brain Region** | **Significant association with EDSS reported?** | | | |
| --- | --- | --- | --- | --- | --- | --- | --- | --- |
|  | Mean (SD) [range] | Median* (SD) [range] |  |  | Lesions | GM | WM | Other |
| Al-Radaideh, Athamneh, et al. (2020) | 3.8 (1.2)  [0.1-14.9] | 2.7* (2.2)  [0-6.5] | MTR | GM, Lesions, Other | 🗶 | - | - | 🗶 |
| Amann, Sprenger, et al. (2015) | 20.8  [4-48] | 3  [0-7] | MTR | NAWM, NAGM, Lesions, Other | ✓ | 🗶 | 🗶 | - |
| Arnold, Gold, et al. (2017) | 5.5 | 2.4* | MTR | NAWB, Lesions | - | - | - | - |
| Arnold, Calabresi, et al. (2014) | - | 2.5* (1.2) | MTR | WB, NAWB | - | - | - | - |
| Audoin, Davies, et al. (2007) | 1.9  [0.5-3.7] | 1.5  [0-3] | MTR | GM | - | 🗶 | - | - |
| Bellmann-Strobl, Stiepani, et al. (2009) | - | - | MTR | NAWM, Lesions | - | 🗶 | - | - |
| Bernitsas, Kopinsky, et al. (2020) | 9.3 (6.3) | 1.8 [1-4] | MTR | NAWM, Lesions | - | - | - | - |
| Bonnier, Roche, et al. (2014) | 2.3 (1.5) | 1.6* (0.3)  [1-2] | MTR | NAWM, NAGM, Lesions, Other | - | - | - | - |
| Bonnier, Roche, et al. (2015) | 2.3 (1.5) | 1.6* (0.3)  [1-2] | MTR | NAWM, GM, Lesions, Other | - | - | - | - |
| Bonnier, Marechal, et al. (2017) | [?-5] | 1.5, 1.6* (0.3) | MTR | NAWM, GM, Lesions, Other | - | - | - | - |
| Bonnier, Fischi-Gomez, et al. (2019) | 2.8 (1.9)  [?-5] | 1.5, 1.6* (0.3) [1-2] | MTR | NAWM, GM, Lesions | - | - | - | - |
| Catalaa, Grossman, et al. (2000) | - | - | MTR | NAWM | - | - | 🗶 | - |
| Cercignani, Iannucci, et al. (2000) | 3.5♣  [1-8] | 1.5  [1-3] | MTR | WB, NAWM, Lesions, Other | 🗶 | - | 🗶 | - |
| Cercignani, Basile, et al. (2009) | 13♣  [1-32] | 2.5  [1-4.5] | MTR, R1f, F, f, T2A, T2B | NAWM, Lesions | ✓/🗶 | - | ✓/🗶 | - |
| Codella, Rocca, et al. (2002) | 8♣ / 6♣  [1-22] /  [3-40] | 1  [0-1] | MTR | GM | - | - | - | - |
| Colasanti, Guo, et al. (2014) | 11.2 (6.9)  [1.5-20] | 4, 4.1* (1.7)  [2-7] | MTR | NAWM, Lesions, Other ROIs | - | - | - | - |
| Cronin, Xu, et al. (2020) | - | - | R1f | NAWB | - | - | - | - |
| Davies, Ramani, et al. (2003) | 11  [1.5-25] | 2  [2-2.5] | MTR, f, T1f, T2B | NAWM, GM, Lesions | - | - | - | - |
| Davies, Ramio-Torrenta, et al. (2004) | 1.9  [0.5-3.7] | 1.5  [0-3] | MTR | NAWM, GM | - | ✓ | 🗶 | - |
| Davies, Altmann, Hadjiprocopis et al. (2005) | 1.9♣  [0.5-3.7] | 1  [0-3] | MTR | NAWM, GM | - | 🗶 | 🗶 | - |
| Davies, Altmann, Rashid, et al. (2005) | 1.9  [0.5-3.7] | 1  [0-3] | MTR | Other ROIs | - | - | - | ✓/🗶 |
| Deloire, Ruet, et al. (2011) | 2 (2.3) | 2  [0-5.5] | MTR | NAWB, Lesions | - | - | - | - |
| De Stefano, Narayanan, et al. (2002) | 2.0 (2.3) | 2  [0-5.5] | MTR | NAWM | - | - | - | - |
| Dortch, Li, et al. (2011) | - | - | R1f, Sf, M0f, F, kf | NAWM, GM, Lesions, Other ROIs | - | - | - | - |
| Dortch, Bagnato, et al. (2018) | - | - | R1f, Sf, M0f, F, kf | NAWM, GM, Lesions, Other ROIs | - | - | - | - |
| Ernst, Chang, et al. (1998) | - | 3.5 | MTR | NAWM, Lesions, Other ROIs | - | - | - | - |
| Fatemidokht, Harirchian, et al. (2020) | [2-15] | [0-5] | MTR | Lesions | - | - | - | - |
| Fazekas, Ropele, et al. (2002) | - | 2.2*  [1.5-3.5] | Kf, T1f | NAWM, Lesions | - | - | - | - |
| Filippi, Rocca, et al. (1998) | 4.4  [2-7] | 2  [1.5-2.5] | MTR | NAWM, Lesions | - | - | - | - |
| Filippi, Rocca, et al. (1999) | 4.4  [2-7] | 2  [1.5-2.5] | MTR | NAWM, Lesions | - | - | - | - |
| Filippi, Rocca, et al. (2014) | - | - | MTR | NAWB, NAWM, GM, Lesions | - | - | - | - |
| Fooladi, Sharini, et al. (2018) | 5 | 2* | MTR, T1sat, Ksat=MTR/  T1sat | NAWM | - | - | - | - |
| Fooladi, Riyahi Alam, et al. (2020) | 4.8 | 2* | MTR, T1sat, Ksat=MTR/  T1sat | NAWM, Lesions | - | - | - | - |
| Fritz, Keller, et al. (2017) | 11.9 (8.7) | 4  [1-6.5] | MTR | Other ROIs | - | - | - | 🗶 |
| Frohman, Dwyer, et al. (2009) | 7.9 (5.4) | 2.8* (1.1) | MTR | NAWB, NAWM, NAGM, Lesions | - | - | - | - |
| Ge, Grossman, et al. (2001) | 5.3  [1-15] | [0-6.5] | MTR | NAGM | - | ✓/🗶 | - | - |
| Ge, Grossman, et al. (2003) | 4.2  [0.5-10] | 2.6  [1.5] | MTR | NAWM, Lesions | - | - | - | - |
| Giacomini, Levesque, et al. (2009) | 5.3 (5.8) [1-15] | 2, 2.6* (1.2) [1-4] | MTR, F, MMC | NAWM, Lesions | - | - | - | - |
| Goodkin, Rooney, et al. (1998) | 1 | 1.4* | MTR | NAWM, Lesions | - | - | - | - |
| Gracien, Jurcoane, et al. (2016) | 4 (6.5)  [0-25] | 1.4* (0.9)  [0-3] | MTR | NAWM, GM | - | 🗶 | - | - |
| Griffin, Chard, et al. (2002) | 2  [0.6-3] | 1  [0-2.5] | MTR | NAWM, NAGM, Other ROIs | - | - | - | - |
| Guo, Jewells, et al. (2001) | [2-11] | - | MTR | NAWM, Lesions | - | - | - | - |
| Helms, Dathe, et al. (2008) | - | - | MTsat | WB (WM, GM, Lesion) | - | - | - | - |
| Iannucci, Rovaris, et al. (2001) | 6.5♣  [1-20] | 1.5  [0-4.5] | MTR | NAWB | - | - | - | - |
| Kamagata, Zalesky, et al. (2019) | 9.6 (6)  [3-22] | 0.9* (1.1)  [0-3] | MTsat | Other ROIs | - | - | - | - |
| Karampekios, Papanikolaou et al. (2005) | [1-8] | [1-3.5] | MTR, kf, T1f | NAWM, Lesions | - | - | - | - |
| Kita, Goodkin, et al. (2000) | 1 | 1.4 | MTR | Lesions | - | - | - | - |
| Kuhle, Barro, et al. (2016) | 0.5  [0.3-3] | 2*  [1.5-2.5] | MTR | NAWM, NAGM | - | - | - | - |
| Levesque, Giacomini, et al. (2010) | - | [1-4] | R1f, F, kf, T2A, T2B (& MWF) | NAWM, Lesions | - | - | - | - |
| Lin, Tench, et al. (2008) | 7.9 (6) | 3* (1.3) | MTR | Other ROIs | - | - | - | - |
| Mangia, Carpenter, et al. (2014) | 10 (5) | 2.9* (1.2) | MTR | NAWM, NAGM | 🗶 | 🗶 | 🗶 | - |
| McKeithan, Lyttle, et al. (2019) | - | 1.5  [0-6] | F, kf | WM, NAGM | - | - | - | - |
| Mesaros, Rocca, et al. (2010) | 7.2♣  [1.2-27.4] | 2  [1-5] | MTR | NAWB, Lesions | - | - | - | - |
| Miller, Fox, et al. (2015) | - | 2.5* | MTR | WB | - | - | - | - |
| Muhlert, Atzori, et al. (2014) | - | 3.3*  [1-6.5] | MTR | Other ROIs | - | - | - | - |
| O’Muircheart-aigh, Vavasour, et al. (2019) | [?-10] | 2 [0-4] | MTR (& MWF) | NAWM, NAGM, Lesions, Other ROIs | - | - | - | - |
| Oreja-Guevara, Charil, et al. (2006) | 10.4  [1-23] | 1.3  [0-3.5] | MTR | NAWM, GM | 🗶 | ✓ | ✓/🗶 | - |
| Ostuni, Richert, et al. (1999) | [1-9] | [1-8] | MTR | WB | - | - | - | - |
| Patel, Grossman, et al. (1999) | - | - | MTR | WB | - | - | - | - |
| Preziosa, Pagani, et al. (2020) | 9.9 (6.5) | 2 | MTR | Lesions | 🗶 | - | - | - |
| Reich, White, et al. (2015) | 4.2 (3.2) [0.3-8] | 1.5, 1.6* (0.7) [1-2.5] | MTR | Lesions | - | - | - | - |
| Reitz, Hof, et al. (2017) | 1♣  [1-2.5] | 1  [0-2.5] | MTR | NAWM, Lesions | - | - | - | - |
| Richert, Ostuni, et al. (1998) | 3.3 (2.9)  [0.5-8] | 3.5, 3.4* (1.8) [1-6] | MTR | NAWM, GM, Lesions | - | - | - | - |
| Richert, Ostuni, et al. (2001) | 0.2  [0.2-0.3] | 1.5  [1-3.5] | MTR | NAWM, Lesions | - | - | - | - |
| Rocca, Falini, et al. (2002) | 9.5♣  [2-22] | 0  [0-1] | MTR | NAWB, Lesions | - | - | - | - |
| Romascano, Meskaldji, et al. (2015) | 2.7 (1.8) | 1.6* (0.2) | MTR | Other ROIs | - | - | - | - |
| Ropele, Strasser-Fuchs, et al. (2000) | - | [1-5] | MTR, kf, T1f | NAWM, Lesions | - | - | - | - |
| Rovira, Alonso, et al. (1999) | 4.7  [1-14] | 2*  [0-6] | MTR | NAWM, Lesions | - | - | - | - |
| Rudick, Lee, et al. (2006) | 6.1 (5.8) | 2.2* (0.8) | MTR | NAWB, Lesions | - | - | - | - |
| Saccenti, Hagiwara, et al. (2020) | 8.7 (6.5) | 1*  [0-2] | MTsat | NAWM, Lesions | - | - | - | - |
| Schwartz, Tagge, et al. (2019) | 13 | - | MTR | NAWM, GM, Lesions | - | - | - | - |
| Siemonsen, Young, et al. (2016) | 5 (4)  [1-13] | 2  [0-4] | MTR | NAWM, Lesions | 🗶 | - | 🗶 | - |
| Sled, Pike (2001) | - | - | R1f, F, kf, T2A, T2B | NAGM, Lesions, Other ROIs | - | - | - | - |
| Smith, Farrell, et al. (2006) | - | - | MTR | NAWM | - | - | - | - |
| Thaler, Faizy, et al. (2018) | 7 (6.7) | 2.1 (1.5) | MTR | NAWM, Lesions | - | - | - | - |
| Van den Elskamp, Knol, et al. (2010) | 5.8 (6.3) | 2 | MTR | NAWM, Lesions | - | - | - | - |
| Van Obberghen, McHinda, et al. (2018) | 10  [1-22] | 1.7*  [0-6.5] | MTR, ihMTR | NAWM, Lesions, Other ROIs | ✓/🗶 | - | - | ✓/🗶 |
| Weinstock-Guttman, Zivadinov, et al. (2007) | 13.3 (7.8) | 2.5 | MTR | NAWB, NAWM, NAGM, Lesions | - | - | - | - |
| Yarnykh (2012) | - | - | R1f, R(=k(1-f)/f), f, T2A, T2B | WM (unspecified if NAWM), GM, Lesions, Other ROIs | - | - | - | - |
| Yiannakas, Tozer, et al. (2013) | - | 1.5  [0-2.5] | MTR | Lesions | - | - | - | - |
| Zhang, Wen, et al. (2020) | - | - | MTR, ihMTR qihMT (dual) | NAWM, Lesions, Other ROIs | ✓ | - | 🗶 | - |
| Zhou, Zhu, et al. (2004) | 6.5  [1-15] | 2.2*  [1.5-4] | MTR | WB | - | - | - | - |
| Zivadinov, De Masi, et al. (2001) | 5.8 (3.3)  [1-10] | 1.5 (1.7)  [0-5] | MTR | NAWB, Lesions | - | - | - | ✓ |
| Zivadinov, Hussein, et al. (2011) | 9.5 (8.3)  [?-20] | 2.3* (1.5)  [0.5-6.5] | MTR | NAWB, Lesions | - | - | - | - |
| Zivadinov, Ramanathan, et al. (2012) | 12.4/13.1 (9.3/11.1) | 2/2.5, 2.3*/2.4*,  (0.9/1.3) | MTR | NAWB, NAWM, NAGM, Lesions | - | - | - | - |
| Zivadinov, Dwyer, et al. (2014) | 6.6 (5.7) [0-20] | 2.5  [1-5.5] | MTR | NAWB, Lesions | - | - | - | - |

***Supplementary Table 5: Overview of usage of disease-modifying therapies (DMTs) across studies.***

| **Number of DMTs** |  | **n** | **%** | **Citation** |  |
| --- | --- | --- | --- | --- | --- |
| **0** | **No DMTs** | 16 | 18.60 | ^37,40,43-46,48,50,57,65,78,79,81-84^ |  |
|  | **Placebo only** | 1 | 1.16 | ^80^ |  |
| **1** | **1 (or untreated)** | 4 | 4.65 | ^38,39,76,77^ |  |
|  | **1 (all treated)** | 11 | 12.79 | ^58,66-75^ |  |
| **>1** | **No placebo** | 14 | 16.28 | ^35,49,51-56,89,90,93-95,97^ |  |
|  | **With placebo** | 7 | 8.14 | ^61,91,92,96,98-100^ |  |
| **Unknown** | **Unspecified** | 5 | 5.81 | ^59,85-88^ |  |
| **Data missing** | | 28 | 32.56 | ^11,36,41,42,47,60,62-64,101-119^ | |
|  | | | | | |

***Supplementary Table 6: Linear mixed model fit results examining longitudinal change in mean MTR across brain sub-regions***

| **Linear Mixed Model Fit by Maximum Likelihood (*lmer* package in RStudio)** | | | | | | | | | |
| --- | --- | --- | --- | --- | --- | --- | --- | --- | --- |
| **Random effects:** | | | | | | | | | |
|  | | | | | **Variance** | | | **SD** | |
| Study/Subgroup | | (intercept) | | | 5.64 | | | 2.38 | |
| Study | | (intercept) | | | 36.04 | | | 6.00 | |
| Residual | |  | | | 2.65 | | | 1.63 | |
| **Fixed effects:** | | | | | | | | | |
| ***Predictors*** | ***Estimates*** | | ***CI*** | | | ***df*** | ***t-value*** | | ***p*** |
| (intercept) | 45.81 | | 42.06 | 49.58 | | 15.07 | 25.49 | | 0.000*** |
| Time (in years) | 0.12 | | -0.56 | 0.80 | | 126.28 | 0.35 | | 0.724 |
| Cerebellum | 2.31 | | -0.65 | 5.26 | | 125.53 | 1.54 | | 0.125 |
| Internal Capsule | 0.42 | | -2.54 | 3.37 | | 125.53 | 0.28 | | 0.780 |
| Lesions | -10.82 | | -11.79 | -9.84 | | 128.92 | -21.90 | | 0.000*** |
| NABT | -5.73 | | -7.54 | -3.93 | | 132.13 | -6.28 | | 0.000*** |
| NAGM | -7.97 | | -10.80 | -5.16 | | 132.73 | -5.61 | | 0.000*** |
| Pons | 2.35 | | -0.61 | 5.30 | | 125.53 | 1.57 | | 0.119 |
| Thalamus | -8.49 | | -22.80 | 5.80 | | 16.43 | -1.24 | | 0.233 |
| Whole Brain | -6.71 | | -9.08 | -4.36 | | 129.97 | -5.62 | | 0.000*** |
| Time*Cerebellum | -1.56 | | -18.82 | 15.70 | | 123.60 | -0.18 | | 0.858 |
| Time*Internal Capsule | 0.96 | | -16.30 | 18.22 | | 123.60 | 0.11 | | 0.913 |
| Time*Lesions | 0.04 | | -0.76 | 0.85 | | 128.31 | 0.10 | | 0.917 |
| Time*NABT | -0.10 | | -1.06 | 0.87 | | 124.51 | -0.20 | | 0.845 |
| Time*NAGM | -0.20 | | -1.47 | 1.07 | | 124.38 | -0.31 | | 0.754 |
| Time*Pons | -1.68 | | -18.94 | 15.58 | | 123.60 | -0.19 | | 0.848 |
| Time*Thalamus | -0.47 | | -2.84 | 1.90 | | 123.82 | -0.39 | | 0.695 |
| Time*Whole Brain | -0.13 | | -1.56 | 1.30 | | 124.21 | -0.18 | | 0.857 |
|  | | | | | | | | | |
| Number of observations | 154 | |  |  | |  |  | |  |
| Number of Studies/Subgroups | 29 | |  |  | |  |  | |  |
| Number of Studies | 14 | |  |  | |  |  | |  |
| Number of Subjects | 563 | |  |  | |  |  | |  |
|  | | | | | | | | | |
| AIC | BIC | | loglik | deviance | | df. resid |  | |  |
| 731 | 795 | | -344 | 689 | | 133 |  | |  |

*Supplementary Table 7: Linear mixed-model regression results for longitudinal change in mean MTR in normal-appearing brain tissue.*

| **Linear Mixed Model Fit by Maximum Likelihood (*lmer* package in RStudio)** | | | | | | | | | |
| --- | --- | --- | --- | --- | --- | --- | --- | --- | --- |
| **Random effects:** | | | | | | | | | |
|  | | | | | **Variance** | | | **SD** | |
| Study/Subgroup | | (intercept) | | | 0.076 | | | 0.28 | |
| Study | | (intercept) | | | 38.71 | | | 6.22 | |
| Residual | |  | | | 0.040 | | | 0.20 | |
| **Fixed effects:** | | | | | | | | | |
| ***Predictors*** | ***Estimates*** | | ***CI*** | | | ***df*** | ***t-value*** | | ***p*** |
| (intercept) | 37.55 | | 30.85 | 44.25 | | 5.01 | 13.48 | | 0.000*** |
| Time (in years) | -0.12 | | -0.21 | -0.02 | | 14.70 | -2.65 | | 0.019 |
|  | | | | | | | | | |
| Number of observations | 23 | |  |  | |  |  | |  |
| Number of Studies/Subgroups | 8 | |  |  | |  |  | |  |
| Number of Studies | 5 | |  |  | |  |  | |  |
| Number of Subjects | 278 | |  |  | |  |  | |  |
|  | | | | | | | | | |
| AIC | BIC | | loglik | deviance | | df. resid |  | |  |
| 47.8 | 53.5 | | -18.9 | 37.8 | | 18 |  | |  |

*Supplementary Table 8: Results for a linear mixed regression model assessing change in normal-appearing white matter over time.*

| **Linear Mixed Model Fit by Maximum Likelihood (*lmer* package in RStudio)** | | | | | | | | | |
| --- | --- | --- | --- | --- | --- | --- | --- | --- | --- |
| **Random effects:** | | | | | | | | | |
|  | | | | | **Variance** | | | **SD** | |
| Study/Subgroup | | (intercept) | | | 0.64 | | | 0.80 | |
| Study | | (intercept) | | | 59.14 | | | 7.69 | |
| Residual | |  | | | 0.15 | | | 0.39 | |
| **Fixed effects:** | | | | | | | | | |
| ***Predictors*** | ***Estimates*** | | ***CI*** | | | ***df*** | ***t-value*** | | ***p*** |
| (intercept) | 46.46 | | 40.39 | 52.53 | | 7.99 | 17.00 | | 0.000*** |
| Time (in years) | 0.037 | | -0.14 | 0.22 | | 33.97 | 0.41 | | 0.68 |
|  | | | | | | | | | |
| Number of observations | 45 | |  |  | |  |  | |  |
| Number of Studies/Subgroups | 11 | |  |  | |  |  | |  |
| Number of Studies | 8 | |  |  | |  |  | |  |
| Number of Subjects | 100 | |  |  | |  |  | |  |
|  | | | | | | | | | |
| AIC | BIC | | loglik | deviance | | df. resid |  | |  |
| 121.0 | 130.1 | | -55.5 | 111.0 | | 40 |  | |  |
|  |  | |  |  | |  |  | |  |

*Supplementary Table 9: Linear mixed-model results for change in lesion MTR over time.*

| **Linear Mixed Model Fit by Maximum Likelihood (*lmer* package in RStudio)** | | | | | | | | | |
| --- | --- | --- | --- | --- | --- | --- | --- | --- | --- |
| **Random effects:** | | | | | | | | | |
|  | | | | | **Variance** | | | **SD** | |
| Study/Subgroup | | (intercept) | | | 11.75 | | | 3.43 | |
| Study | | (intercept) | | | 34.55 | | | 5.88 | |
| Residual | |  | | | 5.64 | | | 2.38 | |
| **Fixed effects:** | | | | | | | | | |
| ***Predictors*** | ***Estimates*** | | ***CI*** | | | ***df*** | ***t-value*** | | ***p*** |
| (intercept) | 35.21 | | 31.34 | 39.97 | | 11.11 | 17.70 | | 0.000*** |
| Time (in years) | 0.255 | | -0.52 | 1.02 | | 35.13 | 0.67 | | 0.51 |
|  | | | | | | | | | |
| Number of observations | 55 | |  |  | |  |  | |  |
| Number of Studies/Subgroups | 21 | |  |  | |  |  | |  |
| Number of Studies | 11 | |  |  | |  |  | |  |
| Number of Subjects | 223 | |  |  | |  |  | |  |
|  | | | | | | | | | |
| AIC | BIC | | loglik | deviance | | df. resid |  | |  |
| 317 | 327 | | -154 | 307 | | 50 |  | |  |
|  |  | |  |  | |  |  | |  |

***Supplementary Table 10: Risk of bias assessment for studies (k=86) included in systematic review.*** *JBI: Joanna Briggs Institute; MTI: magnetization transfer imaging; NA: not applicable; RRMS: relapsing-remitting multiple sclerosis. Additional notes are added where a given study did not strictly meet one JBI experimental design.*

| **Risk of Bias** | | | | | | | | | | | | | | | | | | | | | | | | | | | | | | | | | | | | | | | | | | | | | | | | | | | | | |
| --- | --- | --- | --- | --- | --- | --- | --- | --- | --- | --- | --- | --- | --- | --- | --- | --- | --- | --- | --- | --- | --- | --- | --- | --- | --- | --- | --- | --- | --- | --- | --- | --- | --- | --- | --- | --- | --- | --- | --- | --- | --- | --- | --- | --- | --- | --- | --- | --- | --- | --- | --- | --- | --- |
| **Study type** | Analytic, observational, case-control studies (prospective or retrospective) | | | | | | | | | | | | | | | | | | | | | | | | | | | | | | | | | | | | | | | | | | | | | | | | | | | | |
| **k** | 53 (61.6%) | | | | | | | | | | | | | | | | | | | | | | | | | | | | | | | | | | | | | | | | | | | | | | | | | | | | |
| **Measure** | JBI Critical Appraisal Checklist for Case Control Studies | | | | | | | | | | | | | | | | | | | | | | | | | | | | | | | | | | | | | | | | | | | | | | | | | | | | |
| **Question Key** | **Q1** | Were the groups comparable other than the presence of disease in cases or absence of disease in controls? (i.e. control group representative of source population that produced the cases) | | | | | | | | | | | | | | | | | | | | | | | | | | | | | | | | | | | | | | | | | | | | | | | | | | | |
|  | **Q2** | Were cases and controls matched appropriately? (i.e. clear definition of source population) | | | | | | | | | | | | | | | | | | | | | | | | | | | | | | | | | | | | | | | | | | | | | | | | | | | |
|  | **Q3** | Were the same criteria used for identification of cases and controls? | | | | | | | | | | | | | | | | | | | | | | | | | | | | | | | | | | | | | | | | | | | | | | | | | | | |
|  | **Q4** | Was exposure (i.e. typically RRMS diagnosis) measured in a standard, valid and reliable way? | | | | | | | | | | | | | | | | | | | | | | | | | | | | | | | | | | | | | | | | | | | | | | | | | | | |
|  | **Q5** | Was exposure measured in the same way for cases and controls? | | | | | | | | | | | | | | | | | | | | | | | | | | | | | | | | | | | | | | | | | | | | | | | | | | | |
|  | **Q6** | Were confounding factors identified? | | | | | | | | | | | | | | | | | | | | | | | | | | | | | | | | | | | | | | | | | | | | | | | | | | | |
|  | **Q7** | Were strategies to deal with confounding factors stated? | | | | | | | | | | | | | | | | | | | | | | | | | | | | | | | | | | | | | | | | | | | | | | | | | | | |
|  | **Q8** | Were outcomes (i.e. typically MTI measure) assessed in a standard, valid and reliable way for cases and controls? | | | | | | | | | | | | | | | | | | | | | | | | | | | | | | | | | | | | | | | | | | | | | | | | | | | |
|  | **Q9** | Was the exposure period of interest long enough to be meaningful? | | | | | | | | | | | | | | | | | | | | | | | | | | | | | | | | | | | | | | | | | | | | | | | | | | | |
|  | **Q10** | Was appropriate statistical analysis (including correction for multiple comparisons where necessary) used? | | | | | | | | | | | | | | | | | | | | | | | | | | | | | | | | | | | | | | | | | | | | | | | | | | | |
|  | | | | | | | | | | | | | | | | | | | | | | | | | | | | | | | | | | | | | | | | | | | | | | | | | | | | | |
| **Citation** | | **Q1** | | | | | **Q2** | | | | | **Q3** | | | | | **Q4** | | | | | **Q5** | | | | **Q6** | | | | **Q7** | | | | | **Q8** | | | | | **Q9** | | | | | **Q10** | | | | **Overall appraisal** | | | **Additional notes** | |
| Al-Radaideh et al. (2020)^101^ | | **✓** | | | | | **✓** | | | | | — | | | | | **✓** | | | | | **✓** | | | | 🗶 | | | | **✓** | | | | | **✓** | | | | | 🗶 | | | | | 🗶 | | | | Good | | |  | |
| Audoin et al. (2007)^57^ | | **✓** | | | | | — | | | | | — | | | | | — | | | | | **✓** | | | | **✓** | | | | **✓** | | | | | 🗶 | | | | | 🗶 | | | | | — | | | | Ok | | |  | |
| Bellmann-Strobl et al. (2009)^58^ | | 🗶 | | | | | — | | | | | **✓** | | | | | **✓** | | | | | **✓** | | | | 🗶 | | | | **✓** | | | | | **✓** | | | | | **✓** | | | | | — | | | | Poor | | |  | |
| Bernitsas et al. (2020)^66^ | | 🗶 | | | | | — | | | | | — | | | | | **✓** | | | | | 🗶 | | | | **✓** | | | | **✓** | | | | | 🗶 | | | | | **✓** | | | | | **✓** | | | | Ok | | |  | |
| Bonnier et al. (2014) ^51^ | | **✓** | | | | | — | | | | | — | | | | | **✓** | | | | | **✓** | | | | **✓** | | | | **✓** | | | | | **✓** | | | | | **✓** | | | | | **✓** | | | | Excellent | | |  | |
| Bonnier et al. (2015)^52^ | | **✓** | | | | | — | | | | | — | | | | | **✓** | | | | | **✓** | | | | **✓** | | | | **✓** | | | | | 🗶 | | | | | 🗶 | | | | | **✓** | | | | Good | | |  | |
| Bonnier et al. (2017)^53^ | | 🗶 | | | | | — | | | | | — | | | | | **✓** | | | | | **✓** | | | | **✓** | | | | **✓** | | | | | **✓** | | | | | **✓** | | | | | **✓** | | | | Good | | |  | |
| Bonnier et al. (2019)^54^ | | **✓** | | | | | 🗶 | | | | | — | | | | | **✓** | | | | | **✓** | | | | 🗶 | | | | 🗶 | | | | | 🗶 | | | | | **✓** | | | | | — | | | | Ok | | |  | |
| Catalaa et al. (2000)^78^ | | 🗶 | | | | | — | | | | | **✓** | | | | | **✓** | | | | | 🗶 | | | | **✓** | | | | 🗶 | | | | | 🗶 | | | | | **✓** | | | | | **✓** | | | | Ok | | |  | |
| Cercignani et al. (2000)^43^ | | **✓** | | | | | — | | | | | **✓** | | | | | **✓** | | | | | **✓** | | | | **✓** | | | | 🗶 | | | | | 🗶 | | | | | **✓** | | | | | **✓** | | | | Ok | | |  | |
| Cercignani et al. (2009)^94^ | | **✓** | | | | | — | | | | | — | | | | | **✓** | | | | | **✓** | | | | **✓** | | | | **✓** | | | | | **✓** | | | | | **✓** | | | | | **✓** | | | | Excellent | | |  | |
| Codella et al. (2002)^44^ | | **✓** | | | | | — | | | | | **✓** | | | | | **✓** | | | | | **✓** | | | | **✓** | | | | **✓** | | | | | **✓** | | | | | **✓** | | | | | **✓** | | | | Good | | |  | |
| Colasanti et al. (2014)^35^ | | **✓** | | | | | **✓** | | | | | **✓** | | | | | **✓** | | | | | **✓** | | | | **✓** | | | | **✓** | | | | | **✓** | | | | | **✓** | | | | | **✓** | | | | Good | | |  | |
| Cronin et al. (2020)^104^ | | — | | | | | — | | | | | — | | | | | — | | | | | **✓** | | | | 🗶 | | | | 🗶 | | | | | 🗶 | | | | | **✓** | | | | | 🗶 | | | | Ok | | | Proof of principle for method | |
| Davies et al. (2003)^36^ | | **✓** | | | | | — | | | | | — | | | | | **✓** | | | | | — | | | | 🗶 | | | | 🗶 | | | | | 🗶 | | | | | **✓** | | | | | **✓** | | | | Ok | | |  | |
| Davies et al. (2004)^37^ | | **✓** | | | | | — | | | | | — | | | | | **✓** | | | | | **✓** | | | | **✓** | | | | **✓** | | | | | **✓** | | | | | **✓** | | | | | **✓** | | | | Excellent | | |  | |
| Davies et al. (2005)^38^ | | 🗶 | | | | | — | | | | | — | | | | | **✓** | | | | | **✓** | | | | **✓** | | | | **✓** | | | | | **✓** | | | | | **✓** | | | | | **✓** | | | | Good | | |  | |
| Davies et al. (2005)^39^ | | 🗶 | | | | | — | | | | | — | | | | | **✓** | | | | | **✓** | | | | **✓** | | | | **✓** | | | | | 🗶 | | | | | **✓** | | | | | 🗶 | | | | Good | | |  | |
| De Stefano et al. (2002)^81^ | | **✓** | | | | | **✓** | | | | | — | | | | | **✓** | | | | | — | | | | **✓** | | | | 🗶 | | | | | 🗶 | | | | | **✓** | | | | | **✓** | | | | Ok | | |  | |
| Dortch et al. (2011)^105^ | | 🗶 | | | | | — | | | | | — | | | | | — | | | | | **✓** | | | | 🗶 | | | | 🗶 | | | | | 🗶 | | | | | — | | | | | 🗶 | | | | Poor | | | Proof of principle for method | |
| Dortch et al. (2018)^106^ | | 🗶 | | | | | — | | | | | — | | | | | — | | | | | **✓** | | | | 🗶 | | | | 🗶 | | | | | **✓** | | | | | — | | | | | **✓** | | | | Ok | | | Proof of principle for method | |
| Fooladi et al. (2018)^108^ | | **✓** | | | | | — | | | | | — | | | | | **✓** | | | | | **✓** | | | | 🗶 | | | | 🗶 | | | | | 🗶 | | | | | **✓** | | | | | 🗶 | | | | Ok | | |  | |
| Fooladi et al. (2020) ^86^ | | **✓** | | | | | — | | | | | **✓** | | | | | **✓** | | | | | **✓** | | | | 🗶 | | | | 🗶 | | | | | **✓** | | | | | **✓** | | | | | **✓** | | | | Good | | |  | |
| Fritz et al. (2017)^62^ | | **✓** | | | | | **✓** | | | | | — | | | | | **✓** | | | | | **✓** | | | | **✓** | | | | **✓** | | | | | **✓** | | | | | **✓** | | | | | **✓** | | | | Excellent | | |  | |
| Frohman et al. (2009)^82^ | | 🗶 | | | | | **✓** | | | | | — | | | | | — | | | | | **✓** | | | | **✓** | | | | **✓** | | | | | 🗶 | | | | | **✓** | | | | | — | | | | OK | | |  | |
| Ge et al. (2001)^109^ | | **✓** | | | | | — | | | | | — | | | | | **✓** | | | | | **✓** | | | | **✓** | | | | **✓** | | | | | **✓** | | | | | **✓** | | | | | **✓** | | | | Good | | |  | |
| Goodkin et al. (1998)^83^ | | **✓** | | | | | — | | | | | — | | | | | **✓** | | | | | **✓** | | | | 🗶 | | | | 🗶 | | | | | **✓** | | | | | **✓** | | | | | **✓** | | | | Good | | |  | |
| Gracien et al. (2016)^89^ | | **✓** | | | | | — | | | | | **✓** | | | | | **✓** | | | | | **✓** | | | | **✓** | | | | 🗶 | | | | | **✓** | | | | | **✓** | | | | | **✓** | | | | Good | | |  | |
| Griffin et al. (2002)^40^ | | **✓** | | | | | — | | | | | — | | | | | **✓** | | | | | **✓** | | | | **✓** | | | | **✓** | | | | | 🗶 | | | | | **✓** | | | | | **✓** | | | | Good | | |  | |
| Helms et al. (2008)^11^ | | **✓** | | | | | — | | | | | — | | | | | **✓** | | | | | **✓** | | | | 🗶 | | | | 🗶 | | | | | 🗶 | | | | | — | | | | | **✓** | | | | Poor | | |  | |
| Iannucci et al. (2001)^47^ | | **✓** | | | | | — | | | | | — | | | | | **✓** | | | | | **✓** | | | | 🗶 | | | | 🗶 | | | | | 🗶 | | | | | **✓** | | | | | **✓** | | | | Ok | | |  | |
| Kamagata et al. (2019)^111^ | | **✓** | | | | | — | | | | | **✓** | | | | | **✓** | | | | | **✓** | | | | **✓** | | | | 🗶 | | | | | 🗶 | | | | | **✓** | | | | | **✓** | | | | Good | | |  | |
| Karampekios et al. (2005)^112^ | | **✓** | | | | | — | | | | | **✓** | | | | | **✓** | | | | | **✓** | | | | 🗶 | | | | 🗶 | | | | | **✓** | | | | | **✓** | | | | | 🗶 | | | | Poor | | |  | |
| Kuhle et al. (2016)^55^ | | **✓** | | | | | **✓** | | | | | **✓** | | | | | **✓** | | | | | **✓** | | | | **✓** | | | | **✓** | | | | | 🗶 | | | | | **✓** | | | | | **✓** | | | | Good | | | Also cohort study | |
| Levesque et al. (2010)^77^ | | 🗶 | | | | | — | | | | | — | | | | | — | | | | | **✓** | | | | 🗶 | | | | 🗶 | | | | | 🗶 | | | | | — | | | | | 🗶 | | | | Poor | | |  | |
| Mangia et al. (2014)^60^ | | **✓** | | | | | **✓** | | | | | **✓** | | | | | **✓** | | | | | **✓** | | | | 🗶 | | | | 🗶 | | | | | 🗶 | | | | | **✓** | | | | | 🗶 | | | | Ok | | |  | |
| McKeithan et al. (2019)^64^ | | 🗶 | | | | | — | | | | | — | | | | | **✓** | | | | | 🗶 | | | | **✓** | | | | **✓** | | | | | 🗶 | | | | | — | | | | | 🗶 | | | | Poor | | |  | |
| Muhlert et al. (2014)^41^ | | **✓** | | | | | — | | | | | — | | | | | **✓** | | | | | **✓** | | | | **✓** | | | | **✓** | | | | | 🗶 | | | | | — | | | | | 🗶 | | | | Ok | | |  | |
| O'Muircheartaigh et al. (2019)^97^ | | **✓** | | | | | — | | | | | **✓** | | | | | **✓** | | | | | **✓** | | | | **✓** | | | | **✓** | | | | | 🗶 | | | | | **✓** | | | | | — | | | | Good | | |  | |
| Ostuni et al. (1999)^113^ | | 🗶 | | | | | — | | | | | — | | | | | **✓** | | | | | **✓** | | | | 🗶 | | | | 🗶 | | | | | **✓** | | | | | **✓** | | | | | **✓** | | | | Good | | |  | |
| Reitz et al. (2017)^90^ | | 🗶 | | | | | — | | | | | **✓** | | | | | — | | | | | **✓** | | | | 🗶 | | | | 🗶 | | | | | **✓** | | | | | **✓** | | | | | 🗶 | | | | Poor | | |  | |
| Richert et al. (1998)^70^ | | **✓** | | | | | — | | | | | — | | | | | **✓** | | | | | **✓** | | | | 🗶 | | | | 🗶 | | | | | **✓** | | | | | **✓** | | | | | 🗶 | | | | Ok | | |  | |
| Rocca et al. (2002)^50^ | | **✓** | | | | | — | | | | | **✓** | | | | | **✓** | | | | | **✓** | | | | **✓** | | | | **✓** | | | | | **✓** | | | | | **✓** | | | | | **✓** | | | | Excellent | | |  | |
| Romascano et al. (2015)^56^ | | **✓** | | | | | — | | | | | — | | | | | **✓** | | | | | **✓** | | | | **✓** | | | | **✓** | | | | | 🗶 | | | | | **✓** | | | | | **✓** | | | | Good | | |  | |
| Ropele et al. (2000)^87^ | | 🗶 | | | | | — | | | | | — | | | | | — | | | | | **✓** | | | | 🗶 | | | | 🗶 | | | | | 🗶 | | | | | **✓** | | | | | — | | | | Ok | | |  | |
| Sled and Pike (2001)^116^ | | 🗶 | | | | | — | | | | | — | | | | | — | | | | | **✓** | | | | 🗶 | | | | 🗶 | | | | | **✓** | | | | | — | | | | | **✓** | | | | Poor | | | Proof of principle for method | |
| Smith et al. (2006)^117^ | | — | | | | | — | | | | | — | | | | | — | | | | | **✓** | | | | 🗶 | | | | 🗶 | | | | | **✓** | | | | | — | | | | | 🗶 | | | | Poor | | | Proof of principle for clinical application | |
| Van Obberghen et al. (2018)^88^ | | **✓** | | | | | — | | | | | **✓** | | | | | **✓** | | | | | **✓** | | | | 🗶 | | | | 🗶 | | | | | 🗶 | | | | | **✓** | | | | | **✓** | | | | Good | | |  | |
| Yarnykh (2012)^118^ | | 🗶 | | | | | — | | | | | — | | | | | — | | | | | **✓** | | | | **✓** | | | | 🗶 | | | | | 🗶 | | | | | — | | | | | **✓** | | | | Ok | | | Proof of principle for method | |
| Zhang et al. (2020)^119^ | | **✓** | | | | | — | | | | | **✓** | | | | | **✓** | | | | | **✓** | | | | **✓** | | | | 🗶 | | | | | 🗶 | | | | | — | | | | | 🗶 | | | | Ok | | |  | |
| Zhou et al. (2004)^103^ | | 🗶 | | | | | **✓** | | | | | — | | | | | **✓** | | | | | **✓** | | | | 🗶 | | | | 🗶 | | | | | 🗶 | | | | | **✓** | | | | | 🗶 | | | | Ok | | |  | |
| Zivadinov et al. (2011)^75^ | | **✓** | | | | | — | | | | | — | | | | | **✓** | | | | | **✓** | | | | 🗶 | | | | 🗶 | | | | | **✓** | | | | | **✓** | | | | | **✓** | | | | Good | | |  | |
| Zivadinov et al. (2014)^69^ | | **✓** | | | | | — | | | | | **✓** | | | | | **✓** | | | | | **✓** | | | | **✓** | | | | **✓** | | | | | 🗶 | | | | | — | | | | | **✓** | | | | Ok | | |  | |
|  | | | | | | | | | | | | | | | | | | | | | | | | | | | | | | | | | | | | | | | | | | | | | | | | | | | | | |
| **Study type** | Analytic, experimental, clinical trial | | | | | | | | | | | | | | | | | | | | | | | | | | | | | | | | | | | | | | | | | | | | | | | | | | | | |
| **k** | 4 (4.7%) | | | | | | | | | | | | | | | | | | | | | | | | | | | | | | | | | | | | | | | | | | | | | | | | | | | | |
| **Measure** | JBI Checklist for Randomized Controlled Trials | | | | | | | | | | | | | | | | | | | | | | | | | | | | | | | | | | | | | | | | | | | | | | | | | | | | |
| **Question Key** | **Q1** | Was true randomization used for assignment of participants to treatment groups? | | | | | | | | | | | | | | | | | | | | | | | | | | | | | | | | | | | | | | | | | | | | | | | | | | | |
|  | **Q2** | Was allocation to treatment groups concealed? | | | | | | | | | | | | | | | | | | | | | | | | | | | | | | | | | | | | | | | | | | | | | | | | | | | |
|  | **Q3** | Were treatment groups similar at the baseline? | | | | | | | | | | | | | | | | | | | | | | | | | | | | | | | | | | | | | | | | | | | | | | | | | | | |
|  | **Q4** | Were participants blind to treatment assignment? | | | | | | | | | | | | | | | | | | | | | | | | | | | | | | | | | | | | | | | | | | | | | | | | | | | |
|  | **Q5** | Were those delivering treatment blind to treatment assignment? | | | | | | | | | | | | | | | | | | | | | | | | | | | | | | | | | | | | | | | | | | | | | | | | | | | |
|  | **Q6** | Were outcomes assessors blind to treatment assignment? | | | | | | | | | | | | | | | | | | | | | | | | | | | | | | | | | | | | | | | | | | | | | | | | | | | |
|  | **Q7** | Were treatment groups treated identically other than the intervention of interest? | | | | | | | | | | | | | | | | | | | | | | | | | | | | | | | | | | | | | | | | | | | | | | | | | | | |
|  | **Q8** | Was follow up complete and if not, were differences between groups in terms of their follow up adequately described and analyzed? | | | | | | | | | | | | | | | | | | | | | | | | | | | | | | | | | | | | | | | | | | | | | | | | | | | |
|  | **Q9** | Were participants analyzed in the groups to which they were randomized? | | | | | | | | | | | | | | | | | | | | | | | | | | | | | | | | | | | | | | | | | | | | | | | | | | | |
|  | **Q10** | Were outcomes measured in the same way for treatment groups? | | | | | | | | | | | | | | | | | | | | | | | | | | | | | | | | | | | | | | | | | | | | | | | | | | | |
|  | **Q11** | Were outcomes measured in a reliable way? | | | | | | | | | | | | | | | | | | | | | | | | | | | | | | | | | | | | | | | | | | | | | | | | | | | |
|  | **Q12** | Was appropriate statistical analysis used? | | | | | | | | | | | | | | | | | | | | | | | | | | | | | | | | | | | | | | | | | | | | | | | | | | | |
|  | **Q13** | Was the trial design appropriate, and any deviations from the standard RCT design (individual randomization, parallel groups) accounted for in the conduct and analysis of the trial? | | | | | | | | | | | | | | | | | | | | | | | | | | | | | | | | | | | | | | | | | | | | | | | | | | | |
|  | | | | | | | | | | | | | | | | | | | | | | | | | | | | | | | | | | | | | | | | | | | | | | | | | | | | | |
| **Citation** | **Q1** | | **Q2** | **Q3** | | | | | **Q4** | | | | | **Q5** | | | | **Q6** | | | | | **Q7** | | | | **Q8** | | | | **Q9** | | | | | **Q10** | | | **Q11** | | | | **Q12** | | | | **Q13** | | | | **Overall Appraisal** | **Additional Notes** | |
| Arnold et al. (2014)^91^ | — | | **✓** | **✓** | | | | | **✓** | | | | | **✓** | | | | **✓** | | | | | **✓** | | | | — | | | | 🗶 | | | | | **✓** | | | — | | | | **✓** | | | | **✓** | | | | Good |  | |
| Arnold et al. (2017)^99^ | **✓** | | **✓** | **✓** | | | | | **✓** | | | | | — | | | | **✓** | | | | | **✓** | | | | **✓** | | | | 🗶 | | | | | **✓** | | | — | | | | **✓** | | | | **✓** | | | | Good |  | |
| Filippi et al. (2014)^100^ | **✓** | | **✓** | **✓** | | | | | **✓** | | | | | **✓** | | | | — | | | | | **✓** | | | | 🗶 | | | | 🗶 | | | | | **✓** | | | — | | | | **✓** | | | | **✓** | | | | Ok |  | |
| Miller et al. (2015)^92^ | — | | 🗶 | **✓** | | | | | 🗶 | | | | | 🗶 | | | | **✓** | | | | | 🗶 | | | | **✓** | | | | 🗶 | | | | | **✓** | | | **✓** | | | | **✓** | | | | **✓** | | | | Ok |  | |
|  | | | | | | | | | | | | | | | | | | | | | | | | | | | | | | | | | | | | | | | | | | | | | | | | | | | | | |
| **Study type** | Analytic, observational, cross-sectional | | | | | | | | | | | | | | | | | | | | | | | | | | | | | | | | | | | | | | | | | | | | | | | | | | | | |
| **k** | 11 (12.8%) | | | | | | | | | | | | | | | | | | | | | | | | | | | | | | | | | | | | | | | | | | | | | | | | | | | | |
| **Measure** | JBI Checklist for Analytical Cross-Sectional Studies | | | | | | | | | | | | | | | | | | | | | | | | | | | | | | | | | | | | | | | | | | | | | | | | | | | | |
| **Question Key** | **Q1** | Were the criteria for inclusion in the sample clearly defined? | | | | | | | | | | | | | | | | | | | | | | | | | | | | | | | | | | | | | | | | | | | | | | | | | | | |
|  | **Q2** | Were the study subjects and the setting described in detail? | | | | | | | | | | | | | | | | | | | | | | | | | | | | | | | | | | | | | | | | | | | | | | | | | | | |
|  | **Q3** | Was the exposure measured in a valid and reliable way? | | | | | | | | | | | | | | | | | | | | | | | | | | | | | | | | | | | | | | | | | | | | | | | | | | | |
|  | **Q4** | Were objective, standard criteria used for measurement of the condition? | | | | | | | | | | | | | | | | | | | | | | | | | | | | | | | | | | | | | | | | | | | | | | | | | | | |
|  | **Q5** | Were confounding factors identified? | | | | | | | | | | | | | | | | | | | | | | | | | | | | | | | | | | | | | | | | | | | | | | | | | | | |
|  | **Q6** | Were strategies to deal with confounding factors stated? | | | | | | | | | | | | | | | | | | | | | | | | | | | | | | | | | | | | | | | | | | | | | | | | | | | |
|  | **Q7** | Were the outcomes measured in a valid and reliable way? | | | | | | | | | | | | | | | | | | | | | | | | | | | | | | | | | | | | | | | | | | | | | | | | | | | |
|  | **Q8** | Was appropriate statistical analysis used? | | | | | | | | | | | | | | | | | | | | | | | | | | | | | | | | | | | | | | | | | | | | | | | | | | | |
|  | | | | | | | | | | | | | | | | | | | | | | | | | | | | | | | | | | | | | | | | | | | | | | | | | | | | | |
| **Citation** | | **Q1** | | | | | | **Q2** | | | | | **Q3** | | | | | | **Q4** | | | | | | **Q5** | | | | | | | **Q6** | | | | | **Q7** | | | | | **Q8** | | | | | | **Overall Appraisal** | | | | **Additional Notes** | |
| Amann et al.(2015)^85^ | | **✓** | | | | | | **✓** | | | | | **✓** | | | | | | — | | | | | | **✓** | | | | | | | **✓** | | | | | **✓** | | | | | 🗶 | | | | | | Good | | | |  | |
| Fatemidokht et al. (2020) ^107^ | | **✓** | | | | | | 🗶 | | | | | **✓** | | | | | | **✓** | | | | | | 🗶 | | | | | | | 🗶 | | | | | — | | | | | 🗶 | | | | | | Poor | | | |  | |
| Ge et al. (2003) ^79^ | | **✓** | | | | | | 🗶 | | | | | **✓** | | | | | | 🗶 | | | | | | **✓** | | | | | | | 🗶 | | | | | **✓** | | | | | **✓** | | | | | | Ok | | | |  | |
| Guo et al. (2001)^110^ | | **✓** | | | | | | 🗶 | | | | | 🗶 | | | | | | **✓** | | | | | | 🗶 | | | | | | | 🗶 | | | | | **✓** | | | | | 🗶 | | | | | | Poor | | | |  | |
| Lin et al.(2008)^63^ | | **✓** | | | | | | 🗶 | | | | | **✓** | | | | | | **✓** | | | | | | **✓** | | | | | | | 🗶 | | | | | **✓** | | | | | 🗶 | | | | | | Ok | | | |  | |
| Saccenti et al. (2020)^114^ | | **✓** | | | | | | **✓** | | | | | 🗶 | | | | | | **✓** | | | | | | **✓** | | | | | | | 🗶 | | | | | 🗶 | | | | | 🗶 | | | | | | Poor | | | |  | |
| Siemonsen et al. (2016)^115^ | | **✓** | | | | | | **✓** | | | | | **✓** | | | | | | **✓** | | | | | | 🗶 | | | | | | | 🗶 | | | | | **✓** | | | | | **✓** | | | | | | Good | | | |  | |
| Thaler et al. (2018)^67^ | | **✓** | | | | | | **✓** | | | | | **✓** | | | | | | — | | | | | | 🗶 | | | | | | | 🗶 | | | | | **✓** | | | | | 🗶 | | | | | | Ok | | | |  | |
| Weinstock-Guttman et al. (2007)^102^ | | **✓** | | | | | | 🗶 | | | | | **✓** | | | | | | — | | | | | | **✓** | | | | | | | **✓** | | | | | **✓** | | | | | **✓** | | | | | | Good | | | |  | |
| Yiannakas et al. (2013)^42^ | | **✓** | | | | | | 🗶 | | | | | **✓** | | | | | | — | | | | | | **✓** | | | | | | | 🗶 | | | | | **✓** | | | | | **✓** | | | | | | Good | | | |  | |
| Zivadinov et al. (2001)^65^ | | **✓** | | | | | | **✓** | | | | | **✓** | | | | | | **✓** | | | | | | **✓** | | | | | | | **✓** | | | | | **✓** | | | | | **✓** | | | | | | Excellent | | | |  | |
|  | | | | | | | | | | | | | | | | | | | | | | | | | | | | | | | | | | | | | | | | | | | | | | | | | | | | | |
| **Study type** | Analytic, observational, cohort (prospective or retrospective) | | | | | | | | | | | | | | | | | | | | | | | | | | | | | | | | | | | | | | | | | | | | | | | | | | | | |
| **k** | 8 (9.3%) | | | | | | | | | | | | | | | | | | | | | | | | | | | | | | | | | | | | | | | | | | | | | | | | | | | | |
| **Measure** | JBI Checklist for Cohort Studies | | | | | | | | | | | | | | | | | | | | | | | | | | | | | | | | | | | | | | | | | | | | | | | | | | | | |
| **Question Key** | **Q1** | Were the two groups similar and recruited from the same population? | | | | | | | | | | | | | | | | | | | | | | | | | | | | | | | | | | | | | | | | | | | | | | | | | | | |
|  | **Q2** | Were the exposures measured similarly to assign people to both exposed and unexposed groups? | | | | | | | | | | | | | | | | | | | | | | | | | | | | | | | | | | | | | | | | | | | | | | | | | | | |
|  | **Q3** | Was the exposure measured in a valid and reliable way? | | | | | | | | | | | | | | | | | | | | | | | | | | | | | | | | | | | | | | | | | | | | | | | | | | | |
|  | **Q4** | Were confounding factors identified? | | | | | | | | | | | | | | | | | | | | | | | | | | | | | | | | | | | | | | | | | | | | | | | | | | | |
|  | **Q5** | Were strategies to deal with confounding factors stated? | | | | | | | | | | | | | | | | | | | | | | | | | | | | | | | | | | | | | | | | | | | | | | | | | | | |
|  | **Q6** | Were the groups/participants free of the outcome at the start of the study (or at the moment of exposure)? | | | | | | | | | | | | | | | | | | | | | | | | | | | | | | | | | | | | | | | | | | | | | | | | | | | |
|  | **Q7** | Were the outcomes measured in a valid and reliable way? | | | | | | | | | | | | | | | | | | | | | | | | | | | | | | | | | | | | | | | | | | | | | | | | | | | |
|  | **Q8** | Was the follow up time reported and sufficient to be long enough for outcomes to occur? | | | | | | | | | | | | | | | | | | | | | | | | | | | | | | | | | | | | | | | | | | | | | | | | | | | |
|  | **Q9** | Was follow up complete, and if not, were the reasons to loss to follow up described and explored? | | | | | | | | | | | | | | | | | | | | | | | | | | | | | | | | | | | | | | | | | | | | | | | | | | | |
|  | **Q10** | Were strategies to address incomplete follow up utilized? | | | | | | | | | | | | | | | | | | | | | | | | | | | | | | | | | | | | | | | | | | | | | | | | | | | |
|  | **Q11** | Was appropriate statistical analysis used? | | | | | | | | | | | | | | | | | | | | | | | | | | | | | | | | | | | | | | | | | | | | | | | | | | | |
|  | | | | | | | | | | | | | | | | | | | | | | | | | | | | | | | | | | | | | | | | | | | | | | | | | | | | | |
| **Citation** | | **Q1** | | | **Q2** | | | | | **Q3** | | | | | **Q4** | | | | | **Q5** | | | | **Q6** | | | | **Q7** | | | | | **Q8** | | | | | **Q9** | | | **Q10** | | | | | **Q11** | | | | **Overall Appraisal** | | | **Additional Notes** |
| Deloire et al. (2011)^59^ | | **✓** | | | **✓** | | | | | **✓** | | | | | **✓** | | | | | **✓** | | | | **✓** | | | | **✓** | | | | | **✓** | | | | | 🗶 | | | 🗶 | | | | | 🗶 | | | | Good | | |  |
| Mesaros et al. (2010)^80^ | | NA | | | NA | | | | | **✓** | | | | | **✓** | | | | | **✓** | | | | **✓** | | | | **✓** | | | | | 🗶 | | | | | **✓** | | | NA | | | | | **✓** | | | | Ok | | |  |
| Oreja-Guevara et al. (2006)^48^ | | **✓** | | | **✓** | | | | | **✓** | | | | | 🗶 | | | | | 🗶 | | | | **✓** | | | | **✓** | | | | | 🗶 | | | | | **✓** | | | NA | | | | | 🗶 | | | | Ok | | |  |
| Patel et al. (1999)^76^ | | NA | | | NA | | | | | **✓** | | | | | 🗶 | | | | | 🗶 | | | | **✓** | | | | **✓** | | | | | 🗶 | | | | | **✓** | | | NA | | | | | **✓** | | | | Ok | | |  |
| Preziosa et al. (2020)^49^ | | 🗶 | | | **✓** | | | | | **✓** | | | | | **✓** | | | | | **✓** | | | | 🗶 | | | | — | | | | | **✓** | | | | | **✓** | | | 🗶 | | | | | **✓** | | | | Ok | | |  |
| Rovira et al. (1999)^84^ | | 🗶 | | | **✓** | | | | | **✓** | | | | | 🗶 | | | | | 🗶 | | | | **✓** | | | | — | | | | | **✓** | | | | | **✓** | | | NA | | | | | 🗶 | | | | Ok | | |  |
| Rudick et al. (2006)^61^ | | NA | | | NA | | | | | **✓** | | | | | **✓** | | | | | **✓** | | | | — | | | | **✓** | | | | | **✓** | | | | | **✓** | | | NA | | | | | **✓** | | | | Good | | |  |
| Zivadinov et al. (2012)^74^ | | **✓** | | | **✓** | | | | | — | | | | | **✓** | | | | | **✓** | | | | **✓** | | | | **✓** | | | | | **✓** | | | | | **✓** | | | NA | | | | | **✓** | | | | Excellent | | |  |
|  | | | | | | | | | | | | | | | | | | | | | | | | | | | | | | | | | | | | | | | | | | | | | | | | | | | | | |
| **Study type** | Descriptive, case report | | | | | | | | | | | | | | | | | | | | | | | | | | | | | | | | | | | | | | | | | | | | | | | | | | | | |
| **k** | 1 (1.2%) | | | | | | | | | | | | | | | | | | | | | | | | | | | | | | | | | | | | | | | | | | | | | | | | | | | | |
| **Measure** | JBI Checklist for Case Reports | | | | | | | | | | | | | | | | | | | | | | | | | | | | | | | | | | | | | | | | | | | | | | | | | | | | |
| **Question Key** | **Q1** | Were patient’s demographic characteristics clearly described? | | | | | | | | | | | | | | | | | | | | | | | | | | | | | | | | | | | | | | | | | | | | | | | | | | | |
|  | **Q2** | Was the patient’s history clearly described and presented as a timeline? | | | | | | | | | | | | | | | | | | | | | | | | | | | | | | | | | | | | | | | | | | | | | | | | | | | |
|  | **Q3** | Was the current clinical condition of the patient on presentation clearly described? | | | | | | | | | | | | | | | | | | | | | | | | | | | | | | | | | | | | | | | | | | | | | | | | | | | |
|  | **Q4** | Were diagnostic tests or assessment methods and the results clearly described? | | | | | | | | | | | | | | | | | | | | | | | | | | | | | | | | | | | | | | | | | | | | | | | | | | | |
|  | **Q5** | Was the intervention(s) or treatment procedure(s) clearly described? | | | | | | | | | | | | | | | | | | | | | | | | | | | | | | | | | | | | | | | | | | | | | | | | | | | |
|  | **Q6** | Was the post-intervention clinical condition clearly described? | | | | | | | | | | | | | | | | | | | | | | | | | | | | | | | | | | | | | | | | | | | | | | | | | | | |
|  | **Q7** | Were adverse events (harms) or unanticipated events identified and described? | | | | | | | | | | | | | | | | | | | | | | | | | | | | | | | | | | | | | | | | | | | | | | | | | | | |
|  | **Q8** | Does the case report provide takeaway lessons? | | | | | | | | | | | | | | | | | | | | | | | | | | | | | | | | | | | | | | | | | | | | | | | | | | | |
|  | | | | | | | | | | | | | | | | | | | | | | | | | | | | | | | | | | | | | | | | | | | | | | | | | | | | | |
| **Citation** | | **Q1** | | | | | | **Q2** | | | | | **Q3** | | | | | | **Q4** | | | | | | **Q5** | | | | | | | **Q6** | | | | | **Q7** | | | | | **Q8** | | | | | | **Overall Appraisal** | | | | **Additional Notes** | |
| Ernst et al. (1998)^72^ | | **✓** | | | | | | 🗶 | | | | | **✓** | | | | | | **✓** | | | | | | **✓** | | | | | | | **✓** | | | | | **✓** | | | | | **✓** | | | | | | Good | | | |  | |
|  | | | | | | | | | | | | | | | | | | | | | | | | | | | | | | | | | | | | | | | | | | | | | | | | | | | | | |
| **Study type** | Descriptive, case series (and other related study designs without better classification) | | | | | | | | | | | | | | | | | | | | | | | | | | | | | | | | | | | | | | | | | | | | | | | | | | | | |
| **k** | 9 (10.5%) | | | | | | | | | | | | | | | | | | | | | | | | | | | | | | | | | | | | | | | | | | | | | | | | | | | | |
| **Measure** | JBI Checklist for Case Series | | | | | | | | | | | | | | | | | | | | | | | | | | | | | | | | | | | | | | | | | | | | | | | | | | | | |
| **Question Key** | **Q1** | Were there clear criteria for inclusion in the case series? | | | | | | | | | | | | | | | | | | | | | | | | | | | | | | | | | | | | | | | | | | | | | | | | | | | |
|  | **Q2** | Was the condition measured in a standard, reliable way for all participants included in the case series? | | | | | | | | | | | | | | | | | | | | | | | | | | | | | | | | | | | | | | | | | | | | | | | | | | | |
|  | **Q3** | Were valid methods used for identification of the condition for all participants included in the case series? | | | | | | | | | | | | | | | | | | | | | | | | | | | | | | | | | | | | | | | | | | | | | | | | | | | |
|  | **Q4** | Did the case series have consecutive inclusion of participants? | | | | | | | | | | | | | | | | | | | | | | | | | | | | | | | | | | | | | | | | | | | | | | | | | | | |
|  | **Q5** | Did the case series have complete inclusion of participants? | | | | | | | | | | | | | | | | | | | | | | | | | | | | | | | | | | | | | | | | | | | | | | | | | | | |
|  | **Q6** | Was there clear reporting of the demographics of the participants in the study? | | | | | | | | | | | | | | | | | | | | | | | | | | | | | | | | | | | | | | | | | | | | | | | | | | | |
|  | **Q7** | Was there clear reporting of clinical information of the participants? | | | | | | | | | | | | | | | | | | | | | | | | | | | | | | | | | | | | | | | | | | | | | | | | | | | |
|  | **Q8** | Were the outcomes or follow up results of cases clearly reported? | | | | | | | | | | | | | | | | | | | | | | | | | | | | | | | | | | | | | | | | | | | | | | | | | | | |
|  | **Q9** | Was there clear reporting of the presenting site(s)/clinic(s) demographic information? | | | | | | | | | | | | | | | | | | | | | | | | | | | | | | | | | | | | | | | | | | | | | | | | | | | |
|  | **Q10** | Was statistical analysis appropriate? | | | | | | | | | | | | | | | | | | | | | | | | | | | | | | | | | | | | | | | | | | | | | | | | | | | |
|  | | | | | | | | | | | | | | | | | | | | | | | | | | | | | | | | | | | | | | | | | | | | | | | | | | | | | |
| **Citation** | | **Q1** | | | | **Q2** | | | | | **Q3** | | | | | **Q4** | | | | | **Q5** | | | | **Q6** | | | | **Q7** | | | | | **Q8** | | | | | **Q9** | | | | | **Q10** | | | | **Overall Appraisal** | | | | **Additional Notes** | |
| Fazekas et al. (2002)^96^ | | **✓** | | | | — | | | | | **✓** | | | | | 🗶 | | | | | 🗶 | | | | **✓** | | | | 🗶 | | | | | **✓** | | | | | 🗶 | | | | | **✓** | | | | Poor | | | | Sub-cohort of oral interferon beta-1a trial | |
| Filippi et al. (1998)^45^ | | **✓** | | | | **✓** | | | | | **✓** | | | | | 🗶 | | | | | 🗶 | | | | **✓** | | | | **✓** | | | | | **✓** | | | | | 🗶 | | | | | — | | | | Ok | | | |  | |
| Filippi et al. (1999)^46^ | | **✓** | | | | — | | | | | **✓** | | | | | 🗶 | | | | | 🗶 | | | | **✓** | | | | **✓** | | | | | **✓** | | | | | 🗶 | | | | | **✓** | | | | Good | | | |  | |
| Giacomini et al. (2009)^93^ | | **✓** | | | | — | | | | | — | | | | | 🗶 | | | | | 🗶 | | | | **✓** | | | | **✓** | | | | | **✓** | | | | | 🗶 | | | | | 🗶 | | | | Ok | | | |  | |
| Kita et al. (2000)^73^ | | **✓** | | | | **✓** | | | | | **✓** | | | | | **✓** | | | | | **✓** | | | | 🗶 | | | | 🗶 | | | | | **✓** | | | | | **✓** | | | | | **✓** | | | | Good | | | |  | |
| Reich et al. (2015)^95^ | | **✓** | | | | **✓** | | | | | **✓** | | | | | **✓** | | | | | — | | | | **✓** | | | | **✓** | | | | | **✓** | | | | | 🗶 | | | | | **✓** | | | | Good | | | | Prospective & retrospective cohort for sample size calculation | |
| Richert et al. (2001)^71^ | | **✓** | | | | — | | | | | **✓** | | | | | 🗶 | | | | | 🗶 | | | | **✓** | | | | **✓** | | | | | **✓** | | | | | 🗶 | | | | | **✓** | | | | Ok | | | | Baseline vs. treatment (crossover) study | |
| Schwartz et al. (2019)^68^ | | **✓** | | | | — | | | | | **✓** | | | | | NA | | | | | NA | | | | **✓** | | | | **✓** | | | | | **✓** | | | | | **✓** | | | | | **✓** | | | | Good | | | | Single-subject multisite reliability & repeatability | |
| van den Elskamp et al. (2010)^98^ | | **✓** | | | | — | | | | | — | | | | | 🗶 | | | | | NA | | | | **✓** | | | | **✓** | | | | | **✓** | | | | | **✓** | | | | | **✓** | | | | Good | | | | Sample size calculation | |
|  | | | | | | | | | | | | | | | | | | | | | | | | | | | | | | | | | | | | | | | | | | | | | | | | | | | | | |

Supplementary Figures

*
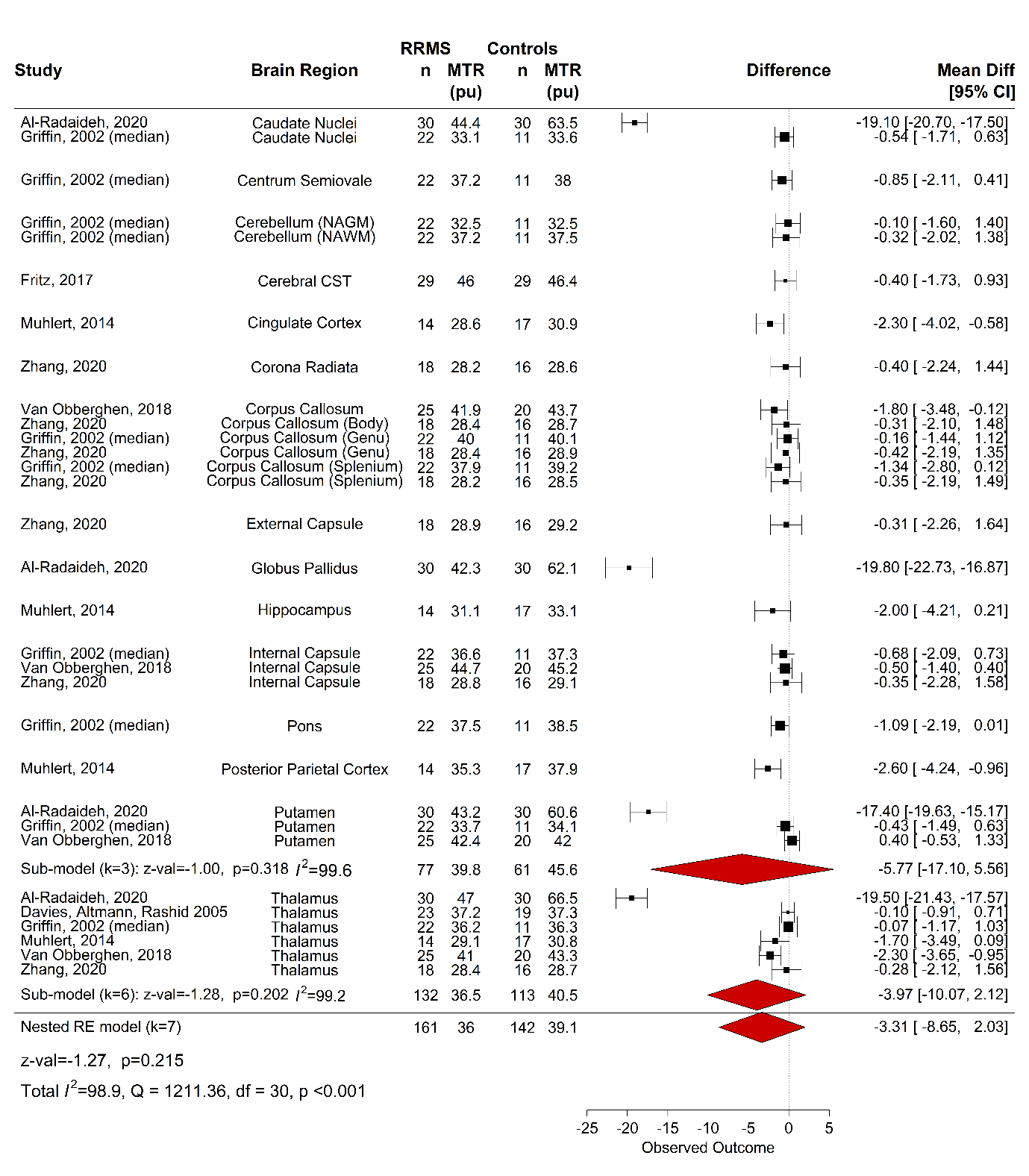
*

**Supplementary Figure 1: *Random-effects meta-analysis to assess the difference in MTR in sub-regions across the brain between patients with relapsing-remitting multiple sclerosis and healthy controls.***  *Study baseline data were used. CST: corticospinal tract; RE: random-effects; mean diff.: absolute mean difference in MTR; 95% CI: 95% confidence interval.*

***Supplementary Figure 2: Predicted longitudinal evolution of magnetisation transfer ratio (MTR)*** *over three years across different brain regions of patients with relapsing-remitting multiple sclerosis, modelled from longitudinal papers with reported means and variance. Marginal mean estimates were calculated from a linear regression model with mean MTR as the dependent variable, timepoint and brain region as fixed effects, and study as a random effect. Further sub-groupings per study were added as an additional nested factor within study (e.g. placebo versus treatment groups, active versus reactive lesions).*


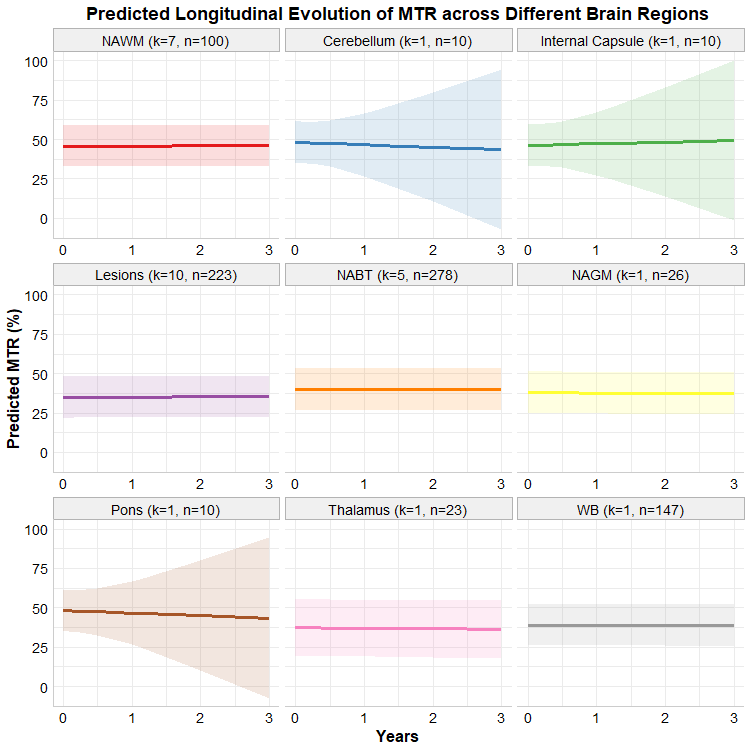


***Supplementary Figure 3: Longitudinal evolution of MTR across different lesion types.*** *APLA: anti-phospholipid antibody status; CEL: contrast-enhancing lesions; Gd: gadolinium. [1a]: CELs (new, active); [1b]: CELs (reactive); [2a]: CELs (standard dose of Gd); [2b] CELs (triple dose of Gd) [3]: CELs; [4a]: nodular-enhancing CELs; [4b]: ring-enhancing CELs; [5a]: isointense; [5b]: hypointense; [6] T1 'black holes'. APLA-pos/neg: anti-phospholipid-positive/negative. Negative months are shown where study classed contrast-enhancement as timepoint zero.*


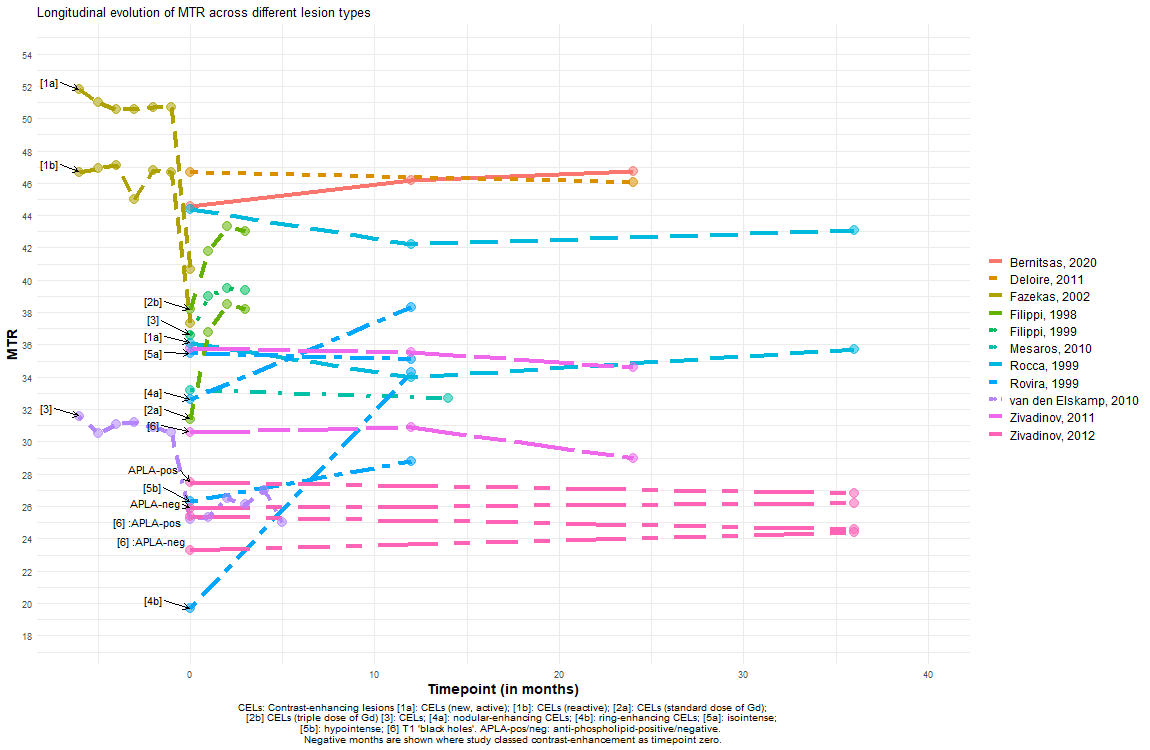


**
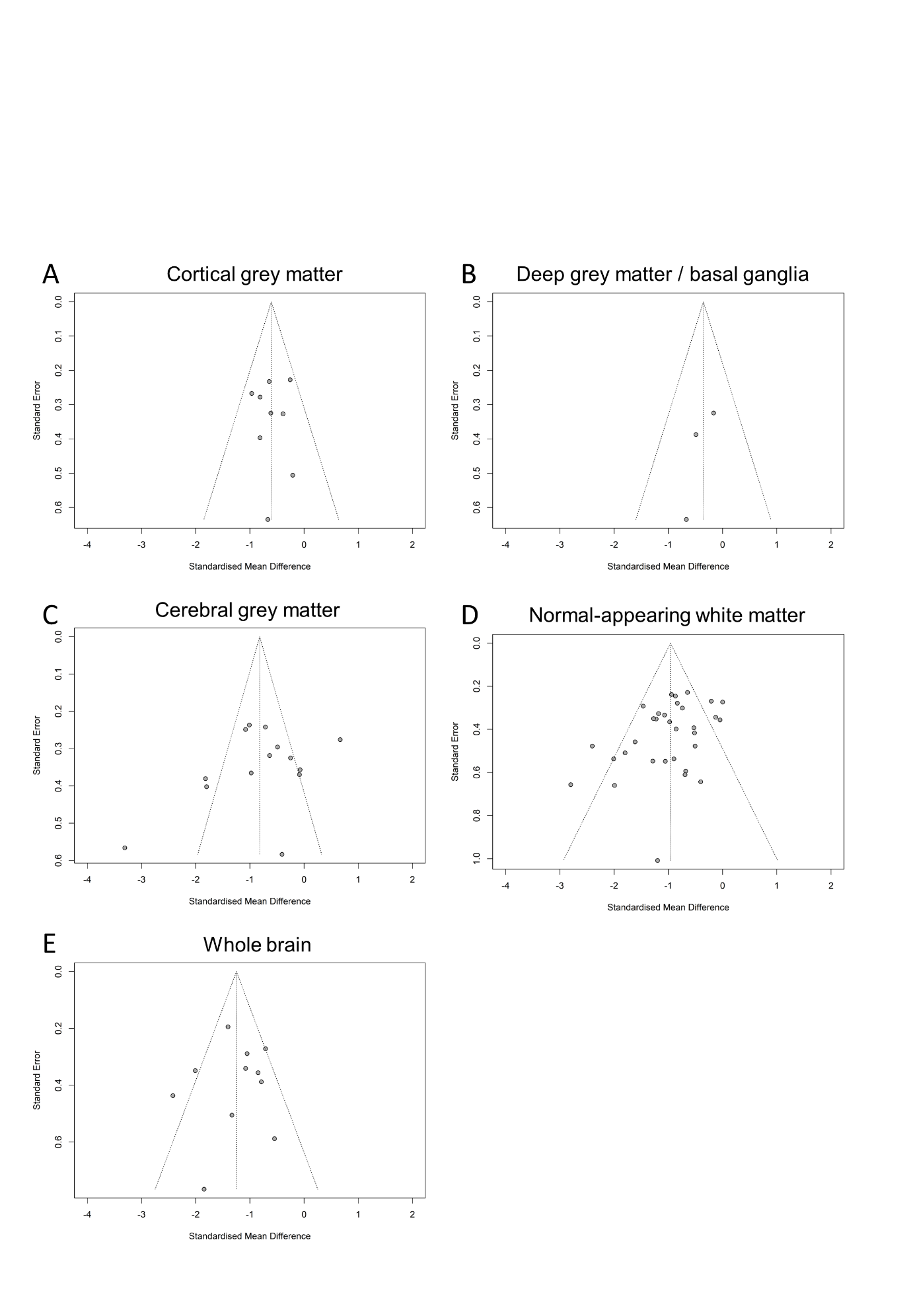
**

***Supplementary Figure 4: Funnel plots show publication/reporting bias for studies which assessed the difference in magnetisation transfer ratio (MTR) between patients with relapsing-remitting multiple sclerosis (RRMS) and healthy controls.*** *A: cortical grey matter; B: deep grey matter/basal ganglia; C: cerebral grey matter; D: normal-appearing white matter; E: whole brain. Vertical line shows random effects model estimated standardised mean difference for each region of interest. Diagonal lines represent pseudo-95% confidence interval. The top-heavy appearance of (D) suggests that smaller, less powerful studies are missing. Similarly, all plots demonstrate a lack of studies with high precision (at triangle peak).*

**
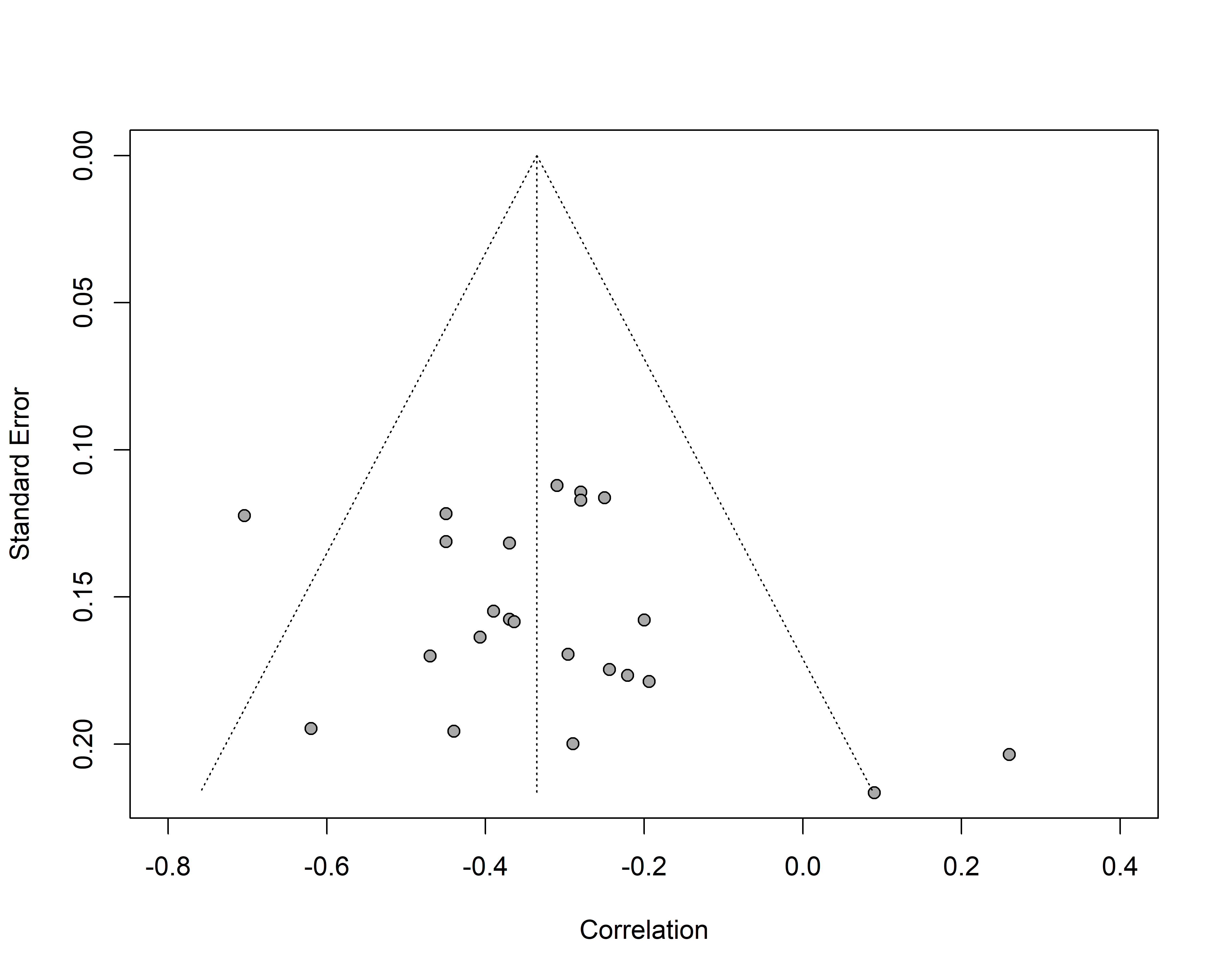
**

***Supplementary Figure 5: Funnel plot show publication/reporting bias for studies which assessed the relationship between magnetisation transfer ratio (MTR) in normal-appearing white matter (NAWM) and clinical disability.*** *Clinical disability was assessed by the Expanded Disability Status Scale (EDSS). Vertical line shows random effects model estimate. Diagonal lines represent pseudo-95% confidence interval. The bottom-heavy appearance suggests a bias towards publication and/or reporting of small, less powerful studies.*

***Supplementary Figure 6: Funnel plot shows publication/reporting bias for studies which used compartmental models to compare patients with RRMS and healthy controls.*** *Vertical line shows random effects model estimate. Diagonal lines represent pseudo-95% confidence interval. The top-heavy appearance suggests relatively high precision across studies, although there are no studies at the peak with very high precision.* **
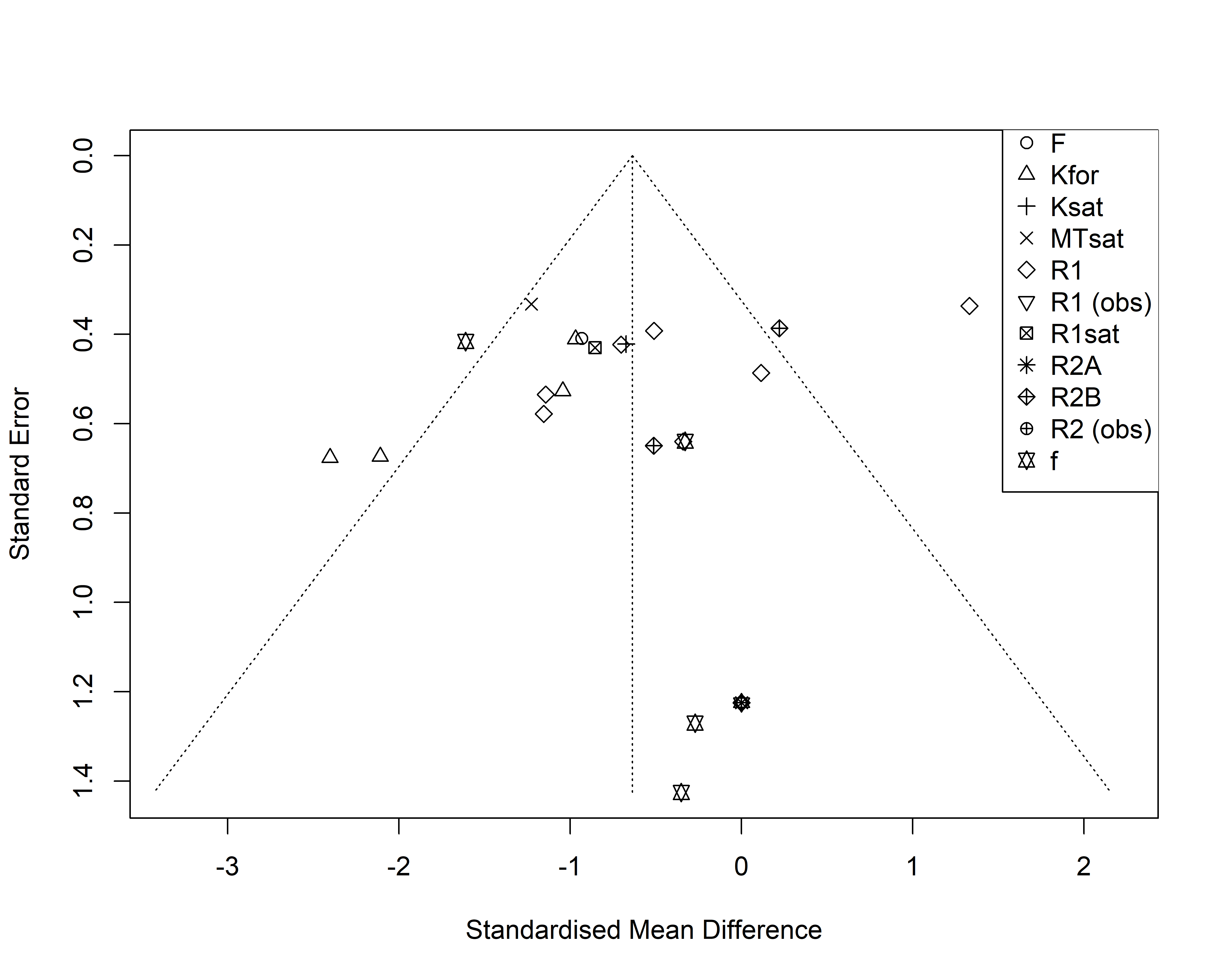
**
